# A randomized controlled trial of self‐help cognitive behavioural therapy for depression in adults with pulmonary hypertension

**DOI:** 10.1111/bjhp.12800

**Published:** 2025-06-12

**Authors:** Abbie S. L. Stark, Gregg H. Rawlings, James D. Gregory, Iain Armstrong, Melanie Simmonds‐Buckley, Andrew R. Thompson

**Affiliations:** ^1^ Cardiff and Vale University Health Board and Cardiff University Cardiff UK; ^2^ Clinical and Applied Psychology Unit University of Sheffield Sheffield UK; ^3^ Sheffield Pulmonary Vascular Disease Unit Royal Hallamshire Hospital, Sheffield Teaching Hospitals NHS Foundation Trust Sheffield UK; ^4^ Rotherham Doncaster and South Humber NHS Foundation Trust Doncaster UK

**Keywords:** low mood, psychological intervention, psychological therapy, pulmonary arterial hypertension, qualitative

## Abstract

**Objectives:**

Pulmonary hypertension (PH) is a progressive, life‐reducing group of conditions associated with an elevated risk of depression. To meet this clinical need, we developed an unguided self‐help intervention targeting depression in PH based on cognitive behavioural therapy (CBT) and tested its acceptability, feasibility, and effectiveness.

**Design:**

A randomized controlled trial (RCT) design was utilized with a wait‐list control group. Acceptability was assessed using content analysis.

**Methods:**

Adults self‐reporting difficulties with depression were recruited from global Pulmonary Hypertension Associations. Participants were randomly assigned to the intervention (*n* = 33) or the wait‐list control group (*n* = 35). Depression was the primary outcome; secondary outcomes included anxiety, health‐related quality of life, pain self‐efficacy, fatigue, and cognitions and behaviours associated with mood difficulties. Change from baseline to post‐intervention (4 weeks) and follow‐up (1 month later) was measured.

**Results:**

We found a significant reduction in depression and HRQoL in the intervention group compared with the control group, with medium effect sizes. No significant changes were observed in other outcomes (*p* > .05). Overall, 72% of individuals in the treatment arm scored above the clinical level of depression, compared with 28% at post‐intervention and 36% at follow‐up. The intervention was judged to be acceptable and feasible, with the main benefits including tools to support, increased motivation, and understanding of depression. No adverse events were reported. Change in cognitions and behaviours did not mediate the relationship between the intervention group and change in depression (*p* > .05).

**Conclusions:**

Results support the use of CBT for depression in PH and provide evidence for the delivery of self‐help at scale via PHA‐UK, the UK's leading charity for PH.

**Statement of Contribution**

**What is already known on this subject?**

- Approximately, one in four adults with pulmonary hypertension experience clinical levels of depression.
- Adults with pulmonary hypertension often report difficulties accessing appropriate evidence‐based psychological intervention for depression.
- There is limited evidence examining psychological treatments for depression in pulmonary hypertension and other long‐term respiratory conditions.

**What does this study add?**

- We have developed and evaluated a novel cognitive behavioural therapy (CBT) self‐help intervention for depression in adults with pulmonary hypertension.
- Results support the use of CBT for depression in adults with PH.
- We demonstrate the viability of providing a psychological intervention for emotional difficulties in pulmonary hypertension via third sector services.

## INTRODUCTION

Pulmonary hypertension (PH) describes a serious and life‐limiting group of conditions characterized by elevated pulmonary artery pressure (Harzheim et al., 2013). Common symptoms include dyspnoea, fatigue, chest pain, and dizziness (Zhou et al., 2020). There are five groups of PH: (i) pulmonary arterial hypertension (PAH), (ii) PH due to left heart disease, (iii) PH due to lung diseases or hypoxia, (iv) chronic thromboembolic PH, and (v) PH with unclear and/or multifactorial mechanisms (Humbert et al., 2022).

The prevalence of psychological distress, specifically mood difficulties, in PH is relatively common (Halimi et al., 2018; Olsson et al., 2021; Pfeuffer‐Jovic et al., 2022; Von Visger et al., 2018). In particular, rates of depression range from 28 to 58% (Bussotti & Sommaruga, 2018; Mai et al., 2022; McCollister et al., 2010). High rates of depression have been observed across geographical regions (North America, Europe and Asia), suggesting depression may be a common experience associated with PH (Mai et al., 2022). The high rate may be somewhat explained by the overlap or interconnection of symptoms of PH and depression, such as fatigue, lack of energy, and poor concentration (American Psychiatric Association, 2022). Verma et al. (2016) propose several factors that could help account for the relationship between PH and depression, including functional impairment, change in quality of life, not coping, non‐compliance with medication, and disease exacerbation. While there has been limited research examining these factors, depression has been found to significantly correlate with health‐related quality of life (HRQoL) in people with PH and therefore could also be a therapeutic target to improve health and well‐being (Harzheim et al., 2013; Zhou et al., 2020).

European Society of Cardiology (2015) guidelines recommend psychological support for individuals with PH. However, there is a lack of high‐quality studies investigating psychological treatments for PH, such as those using a randomized controlled trial (RCT) design (Rawlings et al., 2023). Relaxation training, slow breathing, and Cognitive Behavioural Therapy (CBT) have been recommended for depression in PH (Bussotti & Sommaruga, 2018). CBT‐informed approaches have benefitted people with other cardiovascular conditions, but evidence investigating the effectiveness of CBT for individuals with PH is limited (Bussotti & Sommaruga, 2018; Rawlings, Beail, et al., 2022; Rawlings et al., 2020, 2023; Verma et al., 2016). There is evidence that behaviour change elements associated with CBT (e.g., progressive muscle relaxation) may improve depression in people with PH (Li et al., 2015), and that CBT sessions tailored to PH decrease depression scores (Bussotti & Sommaruga, 2018).

Treatment guidelines in the United Kingdom for depression and depression in adults with a chronic physical health problem recommend a stepped care approach (National Institute for Health and Care Excellence, 2009, 2022a, 2022b). The initial steps involve self‐help approaches, for example based on CBT (National Institute for Health and Care Excellence, 2022a, 2022b). Self‐help offers an approach to therapy that clients' can engage with individually and in their own time. They require less resource than treatments delivered on a one‐to‐one basis or in a group setting. Clients have access to a range of resources, for example informed by a particular therapeutic approach helping them to develop a greater understanding of their difficulties and practice more helpful ways of coping. Self‐help interventions can be cost and clinically effective, and a widely accessible approach to the treatment of mental health difficulties.

There is preliminary evidence for the efficacy of self‐help interventions improving anxiety and depression in PH. Rawlings, Beail, et al. (2022) used a RCT design with an intervention and a wait‐list control group to test an unguided CBT self‐help intervention designed to treat anxiety in PH. Change in mood‐related cognitions and behaviours was a significant mediator of change in anxiety and depression. At the start of the study, 67.6% of participants in the self‐help group scored above the clinical level of depression compared with 32.1% at the end of treatment, with 39.3% reporting a reliable change in depression at the end of the study. However, the study did not recruit people with depression specifically, nor was its primary focus on treating this condition. Therefore, a similar intervention focusing on depression specifically may be more helpful for depression.

The primary aim of this study was to develop and test using a RCT design, the effectiveness of a CBT self‐help intervention targeting depression specifically in adults with PH. Self‐reported symptoms of depression were the primary outcome. Secondary outcomes included anxiety, HRQoL, pain, and fatigue. These were selected due to their relationship with and impact on depression in this clinical group (Halimi et al., 2018; Harzheim et al., 2013) or in individuals with other physical health conditions (Kratz et al., 2017). It was envisaged that the self‐help materials would support individuals to better manage depression in PH. Therefore, it was hypothesised that the intervention group would have a significant reduction in depression scores compared with those in a wait‐list control group at post‐intervention.

Secondary aims included gathering feedback on the acceptability and feasibility of the intervention, and examining potential mediators of change in depression such as cognitions and behaviours, given the finding that unhelpful thoughts and behaviours mediate the relationship between treatment and outcome in mood (Rawlings, Beail, et al., 2022; Rawlings, Thompson, et al., 2022). Acceptability and feasibility are traditionally aims of a pilot study rather than being examined alongside effectiveness. However, many key parameters of the method of intervention delivery and trial design replicated the study by Rawling, Beail, et al. (2022), which tested a CBT self‐help intervention for anxiety and was found to be acceptable and feasible in this clinical group. Therefore, it was deemed appropriate to examine acceptability and feasibility as secondary aims within the present study.

## METHODS

### Design

Participants were randomized on a 1:1 basis to either the CBT informed self‐help intervention for depression or a controlled wait‐list condition. The study was conducted in collaboration with the Pulmonary Hypertension Association UK (PHA‐UK) charity; however, they were not involved in data collection or analysis. Consolidated Standards of Reporting Trials (CONSORT) guidelines were used for reporting of the RCT design (Kwakkenbos et al., 2021; Montgomery et al., 2018). The study was approved by Cardiff University Psychology Ethics Committee and was registered at clinicaltrials.gov.

A two (CBT intervention or wait‐list control) × three (baseline, post and 1‐month follow‐up) design was used. The RCT protocol was checked against the Cochrane risk of bias tool (RoB: Higgins et al., 2019). Outcome measures were completed on Qualtrics by both groups at baseline, post‐intervention (1 month) and at 1‐month follow‐up.

#### Sample size calculation

A power calculation using G*Power 3.1 (Faul et al., 2007) showed a sample size of 77 participants was required using 90% power and a 5% significance level. This was based on a standardised effect size of .53 (Hedges' *g; Cohens' f = .24*), two groups (self‐help and wait‐list) and three measurements (baseline, post‐intervention and follow‐up), and an attrition rate of 15% in accordance with an RCT conducted using the same population, form of recruitment, and similar methodology (Rawlings, Beail, et al., 2022). This calculation was for the primary outcome measure of depression.

### Participants

An opportunity sampling method (Brady, 2006) was utilized as participants were recruited based on their availability and willingness to take part. Participants were recruited from PH Associations globally.

Inclusion criteria were: (a) diagnosis of PH (all forms of PH were included); (b) age 18 years and over; (c) could complete questionnaires without help from others; (d) understood English; (e) able to read all study documents in detail and ask the author any questions; (f) felt they had difficulties with depression or low mood; (g) were not currently experiencing thoughts of self‐harm or suicide – this meant that they had no thoughts of self‐harm or suicide within the last month. Exclusion criteria were: (a) current thoughts of self‐harm or suicide (as based on NICE guidelines [2022] for individuals with depression and a chronic physical health condition). No participants expressed thoughts of self‐harm or suicide throughout the study.

### Measures

A self‐report demographic questionnaire was used which asked about age, gender, ethnicity, employment status, number of years in education, PH diagnosis, World Health Organization (WHO) functional class, length of time since PH diagnosis, and whether the person had been prescribed medication for depression or received psychological therapy for depression within the last 12 months. The WHO functional classes range from I to IV. People with less severe symptoms (WHO functional class I) experience PH‐related symptoms during typical physical activity such as when engaging in household chores; WHO functional class II can be characterized by being comfortable at rest but ordinary physical activity can cause symptoms such as breathlessness and fatigue; functional class III is typified by a greater severity of PH‐related symptoms during routine daily tasks although individuals may still be comfortable at rest; and functional class IV is the inability to engage in physical activity without symptoms – symptoms may also be present at rest (Kiely et al., 2013).

The Patient Health Questionnaire (PHQ‐8) was used to measure depression (Kroenke et al., 2009). The PHQ‐8 has been used in people with PH (Matura et al., 2017) and with individuals who have heart failure and systemic sclerosis with satisfactory validity and reliability reported (Mattsson et al., 2020; Pressler et al., 2011). The PHQ‐9 (which contains the same eight items as the PHQ‐8 plus one additional question asking about thoughts of suicide or self‐harm) has been administered to people with PH previously. The PHQ‐8 was used as per a request from the ethics committee who did not see a need for participants to be asked about thoughts of self‐harm both at the screening (as per exclusion criteria) and data collection stage. The PHQ‐8 has a clinical cut‐off point of ≥10 for defining current depression. A score of 0–4 indicates ‘minimal depression’, 5–9 ‘mild depression’, 10–14 ‘moderate depression’, 15–19 ‘moderately severe depression’ and 20–24 ‘severe depression’ (Kroenke et al., 2009). Good internal consistency was observed in the current study (Cronbach's α = .86), ≥.9 = excellent, ≥.8 = good, ≥.7 = acceptable, ≥.6 = poor, ≤.5 = unacceptable (George & Mallery, 2003).

The Generalized Anxiety Disorder Assessment (GAD‐7) was used to measure anxiety (Spitzer et al., 2006). This has a clinical cut‐off point of ≥8 with a reliable change score of ≥4. A score of 0–4 indicates ‘minimal difficulties’, 5–9 ‘mild’, 10–14 ‘moderate’ and 15–21 ‘severe’ anxiety (Spitzer et al., 2006). The GAD‐7 has good internal consistency (Cronbach's α = .89) (Löwe et al., 2008) and has been used in PH before (Rawlings, Beail, et al., 2022). Excellent internal consistency was reported in the current study (Cronbach's α = .93).

HRQoL was measured using the emPHasis‐10. Scores range from 0 to 50 (Rawlings et al., 2024; Yorke et al., 2014); a higher score indicates lower HRQoL. The emPHasis‐10 has excellent internal reliability (Cronbach's α = .9) and has been shown to be stable across time without intervention (intraclass correlation coefficient = .95) (Yorke et al., 2014). This measure was designed specifically for people with PH. Good internal consistency was demonstrated in the current study (Cronbach's α = .89).

The Fatigue Severity Scale (FSS) was utilised to measure fatigue (Learmonth et al., 2013). Scores range from 9 to 63, with higher scores indicating increased fatigue. Participants rate each item from 1 ‘Strongly Disagree’ to 7 ‘Strongly Agree’. A mean score of >4 for the item ratings indicates severe fatigue. Moderate test‐retest reliability was reported over a 6‐month period in people with multiple sclerosis (Learmonth et al., 2013). Excellent internal consistency was observed in the current study (Cronbach's α = .94). The FSS has previously been administered to people with PH (Sahni et al., 2016; Yorke et al., 2018).

The Pain Self‐Efficacy Questionnaire (PSEQ) was used to measure impact of pain (Nicholas, 2007). Scores range from 0 to 60, with higher scores indicating stronger self‐efficacy beliefs. Participants rate each item from 0 ‘not at all confident’ to 6 ‘completely confident’. Nicholas (2007) reported excellent internal consistency (Cronbach's α = .92), and a high degree of test‐re‐test reliability of up to 3 months, when there is no change in pain or disability. Internal consistency was excellent in the present study (Cronbach's α = .94). We are unaware of any studies that have asked people with PH to complete a pain specific self‐report measure. The PSEQ was used here as other tools tend to focus more on pain symptomology, whereas the PSEQ asks the individual about their thoughts and behaviours related to the impact of pain and how they are coping.

The Cognitive Behavioural Processes Questionnaire (CBPQ) (Patel et al., 2015) was used to explore thoughts and behaviours related to depression and possible targets in treatment. Scores range from 0 to 120, with a higher score being associated with more unhelpful cognitions and behaviours associated with depression (Patel et al., 2015). The CBPQ was used to investigate whether the change in cognitions and behaviours in people with PH mediated the relationship between group and the reduction in depression in the trial by Rawlings, Beail, et al., (2022). Internal consistency was good in the current study (Cronbach's α = .88).

Adherence and acceptability questionnaires consisted of a range of open and closed‐ended questions exploring different aspects of the intervention such as general understanding, difficulty, and helpfulness of the intervention rated from (see Data S1 and S2). They were completed by participants in the intervention arm at 2 weeks and after 1‐month follow‐up (see below). At 2 weeks, we asked participants what booklet they were reading, their progress so far, whether they planned to complete the intervention, and any adverse effects. Questionnaires were developed by members of the research team.

### Procedures

#### Development of the intervention

Medical Research Council Frameworks for complex interventions (Craig et al., 2008; Skivington et al., 2021) and the quality appraisal tool for self‐help interventions in depression (Cape, 2015) were used during the development of the booklets. The intervention consisted of four booklets, which participants were instructed to work through weekly.

Theories of depression were used to inform the booklet content such as the cognitive triad (Beck, 1983; Beck et al., 1979) and the hopelessness theory of depression (Abramson et al., 1989). See Table 1 for a description of the content and exercises within each booklet.

**TABLE 1:** Brief description of the intervention.

Booklet	Content	Exercises
One ‘Depression and pulmonary hypertension’	Introduction to depression and low mood in people living with PH. Psychoeducation and normalizing difficulties of depression in people living with PH, including case studies and quotes from healthcare professionals Introduction to the intervention and how to use the booklets Introduction to the CBT model	Watch two videos about depression and write any thoughts or reflections Complete the four‐areas model Identify any personal negative thinking patterns, repetitive unhelpful thinking styles, avoidance, unhelpful behaviours, low mood and low motivation Two mindfulness exercises
Two ‘Replacing inactivity’	The impact of changing behaviour on low mood Importance of doing meaningful activities Activity scheduling and considering the impact of the physical symptoms of PH Pacing and the boom bust cycle to manage pain and fatigue	Complete an activity scheduling diary (one per week for week two, three and four)
Three ‘Questioning unhelpful thoughts’	Explore the different types of unhelpful thinking styles and understand how they can affect people with PH Develop strategies to identify and question unhelpful thoughts	Watch a video about thoughts not being facts Make notes about your unhelpful thinking patterns Identify more realistic and compassionate alternative thoughts that are fair and accurate The two‐minute rule for managing rumination Problem solving task Complete a thought diary
Four ‘Keeping well’	Psychoeducation on other factors affecting depression such as social support, diet, sleep and physical activity Strategies for keeping well in the context of depression and PH Recognizing and managing setbacks How to continue using the skills in these booklets Signposting to other services for mental health support and for PH	Values identification task including steps to move closer to your values Setback plan including identification of situations that may feed into depression and a management plan A letter to yourself reflecting on what you have learnt and your hopes and fears for the future

The booklets were shared for feedback with healthcare professionals and a readership panel of people with PH and caregivers who were service users of the PHA‐UK charity, prior to being utilized in the study. PHA‐UK supported with the graphic design of the materials, recruitment, and funded the project (see Data S3 for an example of the booklets).

#### Study procedures

Recruitment occurred between June 2023 and January 2024. Adverts for the study were circulated via PH Charity Associations globally through a number of channels, such as their websites, social media, newsletters, and mailing groups. All adverts included a link to the participant information sheet followed by an eligibility screening questionnaire, which assessed individuals' eligibility as per the inclusion and exclusion criteria. Individuals who were not eligible to take part received an automated message with the first author's email address should they have additional questions and signposting to relevant services. Eligible participants were directed to a consent form. Once consent was obtained, they were asked to complete the demographic questionnaire and outcome measures.

Following this, participants were given a unique ID number by the first author and randomly assigned (using https://www.random.org/lists/) to either the CBT group or wait‐list control group in blocks of 10 to allow for equal numbers per group. Following randomization, those in the treatment arm received a copy of the intervention.

The self‐help materials were posted in paper form to those living in the UK and provided in PDF (electronic copy) format for those living outside of the UK. This is consistent with the study by Rawlings, Beail, et al., (2022) and based on how the charity would use the intervention in practice. The charity is contacted by individuals from around the world asking for resources and it is more feasible to send information electronically to those outside of the UK. While we did not record this information, we estimate most participants received the intervention via post or email within 1 week of completing baseline measures. The intervention period consisted of 4 weeks. Participants completed post measures at 1 month (approx. 4–5 weeks after completing baseline measures) and follow‐up measures 1 month later, via Qualtrics. Participants were sent two reminders to complete outcome measures – data collected after 2 weeks of the initial request were not included.

We assessed acceptability at week 2 and 1‐month follow‐up via questionnaires about usage and utility of the booklets: the lead author contacted participants to gather acceptability and adherence feedback via phone for those in the UK and Qualtrics for non‐UK participants. Some participants living in the UK completed the questionnaire via Qualtrics if this was more accessible. The intervention was made available to all participants in the wait‐list control condition in July 2024.

### Data analysis

Descriptive statistics were used to summarize participants' characteristics, examine attrition rates and explore results from acceptability questionnaires. To compare participants in the intervention group and control group at baseline, descriptive statistics and independent *t*‐tests were used for continuous data and Pearson's Chi‐squared tests were used for categorical data. Fisher's exact tests were used for categorical data if any of the cells had fewer than five responses.

All analyses were conducted using SPSS 29 (IBM Corp, 2022) and were based on intention‐to‐treat (ITT) principles, including all participants who were randomized. Overall, 23.5% of participants were missing outcome data: 19% missing at post‐intervention and up to 20.6% missing at follow‐up. Before conducting analyses, an expectation‐maximization algorithm was used to impute missing data separately for each group (Do & Batzoglou, 2008). The two‐way mixed ANOVA (two (intervention and control) × three (pre‐, post‐intervention and 1‐month follow‐up) was used to examine effectiveness of the intervention. The between subjects' factor was the intervention arm (self‐help intervention or wait‐list control) and the within subjects' factor was time (baseline, post‐intervention and 1‐month follow‐up). A two × three mixed ANOVA was performed to compare change in the primary and secondary outcome measures between the intervention and control group. Due to the strong association between depression and anxiety, we performed an explorative analyses of anxiety scores in the treatment group. A sensitivity analysis repeating analyses in the completers sample (also known as a per‐protocol or completers analysis, which includes only participants who completed outcome measures at pre‐intervention, post‐intervention and follow‐up) is reported in the Table S1a–c).

Independent samples *t*‐tests were conducted to examine significant main effects across time. When the interaction (group × time) was significant, independent samples *t*‐tests were conducted to compare differences at each of the three time points (pre‐, post‐intervention and follow‐up) between the intervention and control group. The independent *t*‐tests were conducted with Bonferroni corrections to reduce the chance of a type I error. Effect sizes were calculated using G*Power 3 (Faul et al., 2007) and interpreted using Cohen's *f*; specifically, *f =* .10 indicates a small effect, *f* = .25 medium effect, and *f* = .40 large effect (Cohen, 1988).

A point‐biserial correlation between group and change in depression scores at pre‐intervention versus post‐intervention was conducted to examine whether there was a significant correlation and therefore a rationale for conducting a mediation analysis to explore mechanisms of change. Mediated regression analyses were conducted to examine whether the effects of group on change in depression scores were mediated by change in CBPQ scores. Change in scores was calculated by subtracting scores at baseline from post‐intervention scores. Hayes' process macro extension (Hayes, 2017) for SPSS was used for the mediation analysis. The Sobel test was performed using an online calculator to obtain the *p*‐value (Preacher & Leonardelli, 2024).

#### Content analysis

Content analysis (Hsieh & Shannon, 2005) was used for free‐text responses to identify helpful aspects of the intervention and areas for improvement based on the qualitative data obtained from the intervention group. The responses for each of the questions were amalgamated and read, then separated into codes at a descriptive level. These codes were quantified using frequency counts. Categories were modified and added until all comments were coded.

## RESULTS

### Characteristics of participants

**FIGURE 1:**
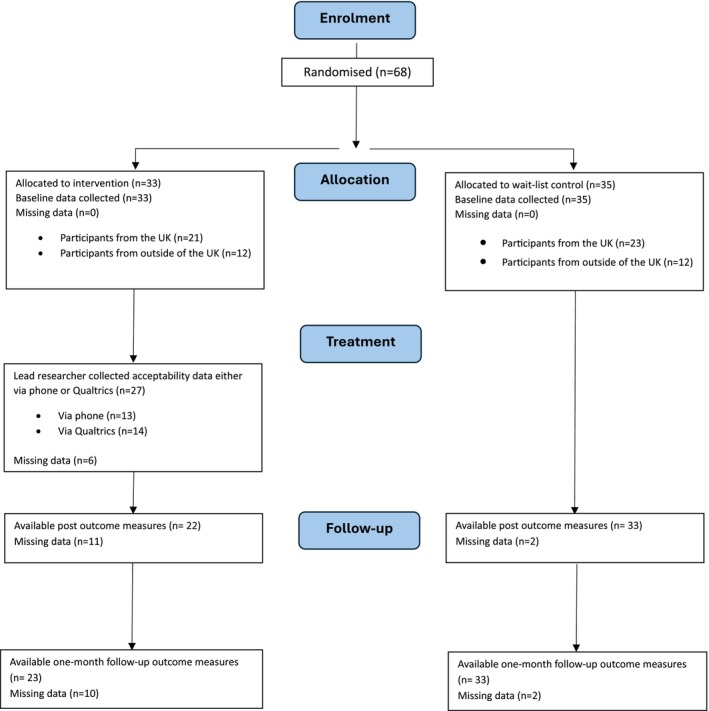
CONSORT flow diagram.

In total, 68 individuals with PH took part in the study (Figure 1). Thirty‐three individuals were allocated to the intervention group and 35 to the control group.

Overall, 85.3% (*n* = 58) self‐reported as female and 14.7% (*n* = 10) male. The mean age of participants was 52.49 (SD = 12.86). Most participants identified themselves as White British (79%, *n* = 54). Diagnoses were as follows: idiopathic PH (32.4%, *n* = 22), connective tissue disease (8.8%, *n* = 6), chronic thromboembolic PH (22.1%, *n* = 15), familial PH (1.5%, = 1), congenital PH (10.3%, *n* = 7), other or not sure (25%, *n* = 17). Participants typically reported their WHO functional class as II (20.6%, *n* = 14) or III (23.5%, *n* = 16); however, the largest group did not report their WHO functional class (41.2%, *n* = 28). Table 2 shows the baseline characteristics for participants in the intervention group and wait‐list control group.

**TABLE 2:** Baseline mean and standard deviation data for participants in the intervention group compared with the control group.

Characteristics	Intervention group	Control group	*p‐*value
Number of participants	*n* = 33	*n* = 35	
Age	55.30 (13.53)	51.71 (12.35)	.61
Gender			1.00
Male	*n* = 5	*n* = 5	
Female	*n* = 28	*n* = 30	
Ethnicity			.69
White	*n* = 27	*n* = 27	
Asian or Asian British	*n* = 2	*n* = 3	
Black, Black British, Caribbean or African	*n* = 1	*n* = 0	
Mixed or multiple ethnic groups	*n* = 2	*n* = 1	
Other ethnic groups	*n* = 0	*n* = 0	
Unknown	*n* = 1	*n* = 4	
Education (years)	15.97 (5.20)	14.88 (5.02)	.43
Employment			.34
Full time	*n* = 10	*n* = 11	
Part time	*n* = 4	*n* = 4	
Not employed	*n* = 5	*n* = 11	
Retired	*n* = 14	*n* = 9	
PH type			.37
Idiopathic PH	*n* = 12	*n* = 10	
Connective tissue disease	*n* = 4	*n* = 2	
Chronic thromboembolic PH	*n* = 8	*n* = 7	
Familial PH	*n* = 0	*n* = 1	
Congenital PH	*n* = 1	*n* = 6	
Other or not sure	*n* = 8	*n* = 9	
PH functional Class			.29
I	*n* = 3	*n* = 3	
II	*n* = 8	*n* = 6	
III	*n* = 4	*n* = 12	
IV	*n* = 2	*n* = 2	
Not sure	*n* = 16	*n* = 12	
Number of years since diagnosis	7.40 (7.77)	10.90 (12.70)	.17
Prescribed medication for depression	*n* = 15 (45.45%)	*n* = 19 (54.29%)	.47
Received therapy for depression	*n* = 8 (24.24%)	*n* = 8 (22.86%)	.89
Depression	13.18 (5.18)	13.14 (5.54)	.61
Anxiety	9.36 (6.03)	11.11 (5.73)	.64
HRQoL	31.21 (9.10)	31.09 (9.80)	.69
Pain	29.55 (11.26)	29.86 (15.09)	.12
Fatigue	53.55 (9.83)	50.03 (12.48)	.38
Cognitions and behaviours (CBPQ)	68.82 (17.86)	71.51 (17.17)	.54

*Note*: Prescribed medication for depression within the last 12 months; received therapy for depression within the last 12 months.

Abbreviations: *n*, number of participants; PH, pulmonary hypertension; HRQoL, Health‐Related Quality of Life; CBPQ, Cognitive and Behavioural Processes Questionnaire.

Both the intervention and control group on the group level scored above the clinical cut‐off of ≥10 for the PHQ‐8 and ≥8 for the GAD‐7 at baseline, scoring in the moderate range.

There were no statistically significant differences in baseline characteristics between participants in the intervention and wait‐list control group (see Table 2). To explore whether it was appropriate to group people living in and out of the UK given differences in healthcare and support, we compared the two groups. There were significant differences between participants on ethnicity and WHO functional class (see Table S2). The rest of the analyses were not significant. Therefore, it was deemed appropriate to group participants in the main analyses (see limitations for more information).

#### Attrition rates

A significantly higher number of participants dropped out of the intervention group compared with the control group. Overall, 10 participants dropped out (14.71%): eight (80%) in the intervention group and two (20%) in the control group. While not systematically collected, five participants who dropped out of the intervention group provided a reason, which included hospitalisation for a health condition (*n* = 2), family illness (*n* = 1), finding the intervention stressful alongside a busy schedule and workload (*n* = 1), and one participant living outside of the UK preferred a paper copy of the intervention rather than an electronic version. Approximately 13 participants signed up to the study but did not complete baseline measures.

On the group level, participants who remained in the study scored above the clinical cut‐off ≥8 on the GAD‐7, which suggested moderate anxiety, whereas those who dropped out of the study scored below the clinical cut‐off, which suggested mild anxiety (see Table S3). No other significant differences were found.

#### Effectiveness

A series of two‐way mixed ANOVAs showed statistically significant interactions between group and time for depression (medium effect) and HRQoL (medium effect) (Table 3). None of the other outcomes were significant.

**TABLE 3:** Means and standard deviations in the intervention (*n* = 33) and control (*n* = 35) group for primary and secondary outcome measures, including results of 2 × 3 mixed ANOVAs.

Outcome measure	Group	Baseline	Post	Follow‐up (1‐month)	Two‐way mixed ANOVA (group × time) *F*	*p*	η^2^	Cohens' *f*
Depression	CBT Control	13.15 (5.22) 13.14 (5.54)	8.82 (5.28) 11.74 (6.11)	8.45 (4.58) 10.89 (6.20)	*F* (1.798, 118.672) = 4.870 GG	.012*	.069	.27
Anxiety	CBT Control	9.36 (6.03) 11.11 (5.73)	6.76 (5.32) 10.34 (6.02)	5.82 (4.84) 9.00 (6.64)	*F* (1.649, 108.841) = 1.873 GG	.165	.028	.17
HRQoL	CBT Control	31.21 (9.10) 31.09 (9.80)	30.97 (8.43) 30.00 (11.70)	27.67 (10.25) 30.34 (11.68)	*F* (2, 132) = 3.985	.021*	.057	.25
Pain	CBT Control	29.55 (11.26) 29.86 (15.09)	34.03 (9.24)^†^ 32.03 (15.02)	34.12 (11.32) 34.37 (12.31)	*F* (1.716, 113.236) = .668 GG	.493	.010	.10
Fatigue	CBT Control	53.55 (9.83) 50.03 (12.48)	50.09 (12.73) 49.11 (11.34)	49.52 (9.93) 48.91 (12.95)	*F* (2,132) = 2.057	.132	.030	.18
Cognitions and Behaviours (CBPQ)	CBT Control	68.82 (17.86) 71.51 (17.17)	57.52 (15.62) 67.60 (22.88)	50.67 (14.90) 59.69 (24.52)	*F* (1.447, 95.475) = 1.850 GG	.173	.027	.17

Abbreviations: CBPQ, Cognitive and Behavioural Processes Questionnaire; GG, Greenhouse Geisser correction used; HRQoL, Health‐Related Quality of Life.

* Statistically significant *p* < .05.

^†^ Levene's Test of Equality not met (*p* < .05).

The intervention group reported lower levels of depression following treatment compared with the control group. For depression in the intervention group, there was a statistically significant difference between pre‐ and post‐intervention, with lower scores reported at the post‐intervention stage (Table 4). There was also a statistically significant difference between pre‐intervention and follow‐up, with lower scores reported at follow‐up. There was not a statistically significant difference between post‐intervention and follow‐up. In comparison, the control group showed a significant difference overall at baseline vs. follow‐up, with a reduction in depression scores, but no significant changes between pre‐ vs. post‐intervention or post‐intervention vs. follow‐up.

**TABLE 4:** Pairwise comparisons using Bonferroni correction for significant interactions between Time × Group.

Outcome measure	Group	Pairwise comparisons		
		Baseline vs. post	Baseline vs. follow‐up	Post vs. follow‐up
Depression	CBT Control	*p <* .001* *p* = .059	*p <* .001* *p* = .005*	*p =* .543 *p* = .142
HRQoL	CBT Control	*p* = .796 *p* = .236	*p <* .001* *p* = .436	*p* = .002* *p* = .725

* Statistically significant as *p* ≤ .017.

For the HRQoL measure, although those in the intervention group did not show any significant changes pre‐ post‐intervention, they did show significant improvements between pre‐intervention– follow‐up and post‐intervention– follow‐up. In comparison, the control group showed no significant differences at any time points.

For the significant main effects of time (Table 5), there was a significant difference between pre‐ vs. post‐intervention and pre‐intervention vs. follow‐up, showing reductions in anxiety, pain, fatigue, and unhelpful thoughts and behaviours (CBPQ). There were also significant reductions in anxiety and unhelpful thoughts and behaviours (CBPQ) between post‐intervention and follow‐up, but this was not seen for pain and fatigue. There were no significant differences between the intervention and control group for CBPQ, pain, or fatigue. However, there was a significant main effect of group for overall anxiety (*F* (1, 66) = 4.882, *p* = .031), with lower anxiety in the intervention group compared with the control group.

**TABLE 5:** Pairwise Comparisons using Bonferroni correction for significant main effect of time.

Outcome measure	Pairwise comparisons		
	Baseline vs. post	Baseline vs. follow‐up	Post vs. follow‐up
Anxiety	*p* < .001*	*p* < .001*	*p* = .008*
Pain	*p* = .010*	*p* < .001*	*p* = .171
Fatigue	*p* = .006*	*p* = .001*	*p* = .637
Cognitions and Behaviours (CBPQ)	*p* < .001*	*p* < .001*	*p* < .001*

* Statistically significant as *p* ≤ .017.

A one‐way repeated measures ANOVA for the anxiety scores in the intervention group across time (pre‐ vs. post‐intervention vs. follow‐up) showed a significant main effect of time (*F* (2, 64) = 14.158, *p* < .001). Mauchly's test of sphericity was not significant (*p* > .05) and equality of variances was met. Pairwise comparisons using Bonferroni corrections suggested that the difference between pre‐ vs. post‐intervention scores was significant (*p* < .001), and the pre‐ vs. follow‐up scores (<.001), post‐intervention vs. follow‐up was not significant (*p* = .482). This suggests that there is a significant difference over time in the intervention group between pre‐ vs. post‐intervention and pre‐ vs. follow‐up for anxiety, with a reduction in anxiety; however, this does not reach significance when compared with the control group interaction.

For depression, scores in the intervention group reduced below the clinical cut‐off and to the ‘mild’ range at post‐intervention, with the mean score reducing further at follow‐up but remaining in the ‘mild’ range. The control group remained in the moderate range and above the clinical cut‐off at post‐intervention and follow‐up. The results were the same for anxiety (see Figures S4 and S5).

On the individual level, 72% of individuals who were randomized to the treatment group and completed the intervention scored above the clinical level of depression, compared with 30.43% at post‐intervention and 39.13% at follow‐up. In the control group, 72.7% scored above the cut‐off at baseline, 66.7% after the intervention, and 48.48% at follow‐up. A reliable change index of 5 or more from baseline demonstrated in the intervention group, 40.9% of individuals at post‐intervention and 41.7% at follow‐up reported a reliable change. In the control group, 21.2% of participants reported a reliable change at post‐intervention and 36.4% at follow‐up.

##### Mediator analyses

A point‐biserial correlation showed a positive correlation between condition and change in depression scores pre‐ vs. post‐intervention, which was statistically significant (*r*_pb_ = .33, *n* = 68, *p* = .007). This supports the rationale for the mediation analysis between group, change in PHQ, and change in CBPQ at pre‐ vs. post‐intervention.

Condition did not significantly predict change in CBPQ scores pre‐ vs. post‐intervention (*b* = 7.389 [se = 4.197], 95% CI [−.992, 15.769], *p* = .083) but did significantly predict change in depression scores (*b* = 2.388 [se = 1.031], 95% CI [.330, 4.46], *p* = .024). Change in CBPQ was a significant predictor of change in depression (*b* = .074 [se = .030], 95% CI [.015, .133], *p* = .015). However, change in CBPQ did not significantly mediate the relationship between depression and group (*b* = .545 [se = .413], 95% CI [−.065, 1.544]). This was confirmed using a Sobel test (Z = −1.433 [se = .38], *p* = .152).

### Qualitative results

Twenty‐eight out of 33 participants in the intervention group provided feedback at 2 weeks (Table S4). Most participants were working on booklet one (32.1%, *n* = 9) or booklet two (35.7%, *n* = 10) with the rest working on booklet three (21.4%, *n* = 6) or four (3.6%, *n* = 1), (7.1%, unknown *n* = 2).

Most participants said they spent ‘a lot’ or ‘a great deal’ of time looking at the booklets (59.3%, 16 out of 27), understood the content (92.3%, 24 out of 26) and felt it could help with their depression (84.6%, 22 out of 26) and other areas of their life (80.8%, 21 out of 26). Twenty‐six out of 28 participants (92.9%) reported intending to finish the intervention, one of these participants dropped out after this stage.

Four participants living outside of the UK reported finding the intervention distressing and were automatically sign posted to contacts and resources for support via Qualtrics. Three of these participants provided positive feedback about the helpfulness of the intervention and stated they wished to continue taking part. The fourth participant dropped out at this stage, stating it was ‘another bit of work to my already overloaded schedule and workload’.

Fifteen out of 25 participants (60%) in the intervention group completed the final acceptability questionnaire (Table S5). Most participants rated the booklets overall as ‘very’ or ‘extremely helpful’ (73.3%, *n* = 11). The ratings for each booklet suggest that all four booklets were helpful. However, participants found the letter writing task (reflecting on what they have learnt and their hopes and fears for the future) less helpful. The majority of participants (86.7%, *n* = 13) reported the intervention as ‘good’ or ‘excellent’. Most participants valued that the intervention was specific to pulmonary hypertension (86.7%, *n* = 13) and thought that the booklets would be beneficial to people when diagnosed with pulmonary hypertension (80%, *n* = 12 rated ‘agree’ or ‘strongly agree’). Fifteen participants provided qualitative data. Five categories were identified based on the questions that were asked (Table 6). Most (73.3%, *n* = 11) participants stated wanting to take part in the intervention for support with depression and their PH. Participants described the main benefits of taking part as having tools to support them, increasing their understanding of depression and increasing their motivation to do things. Nearly all of the participants (*n* = 14) said they planned to re‐visit the booklets and/or put into practice the skills they learnt. Participants viewed the content of the intervention as personally relevant, easy to read and demonstrating an understanding of PH. Some of the barriers to engaging with the booklets included too much information (*n* = 2) and a lack of guidance (*n* = 2). Eight participants did not offer any suggestions for improvements.

**TABLE 6:** Content analysis results (*n* = 15).

Categories	Codes	Participant quote examples	*n*
Reason for taking part	Interested in PH research	‘Interested in research about PH’ (P101)	2
	To help others	‘I wanted to take part in this study to support the well‐being of other patients especially those who are newly diagnosed’. (P112)	3
	For support with depression and PH	‘I have depression and anxiety as well as post‐capillary pulmonary hypertension. I want to learn how to deal with my emotions and improve my mental health’. (P162)	11
	Recent PH diagnosis	‘I was recently diagnosed with pulmonary arterial hypertension’. (P137)	2
	Heard about benefits of mindfulness	**‘**I have heard a lot of positive feedback about the benefits of mindfulness and was interested in giving it a go when I was offered the opportunity to participate in this study’. (P150)	1
Impact of taking part	Helped me talk to others about my feelings	‘It made me talk to my loved ones about my feelings in a way I hadn't before’. (P114)	4
	Normalized my experiences	‘Identify that it's not just me, and my thoughts and feelings are also felt and experienced by others also’. (P108)	3
	Improved how I understand depression	‘It enabled me to think about how my depression is affecting other parts of my life and reinforcing the cycle of depression’. (P162)	5
	Gave me tools to help	‘It taught me how to keep things in the moment’. (P150)	8
	Increased my motivation to do things	‘I have started to partake new responsibilities and challenges I have set new goals for myself’. (P156)	5
Plans for continuing to use what you learnt from the booklets	Re‐visit the booklets	‘To continue using the booklets on a regular basis until it becomes my norm’. (P108)	7
	Put into practice the skills learnt	‘Not doing the activities will inhibit my mood and by recognizing my early warning signs and sticking to a healthy lifestyle my depression will be less severe’. (P112)	7
	Talk to people about my feelings	‘I talk to people I am close to about my difficulties’	2
What was liked the most about the intervention	Personal relevance	‘That it was centred around issues that I actually deal with’ (P162)	5
	Strategies to support my physical and mental health	‘The self‐help intervention gave useful strategies to help maintain my mental health and to make sure I am doing what I need to do to support my physical and mental health’. (P112)	2
	Understanding of PH	‘it's great to have something just for PH’. (P114)	3
	Layout of the booklets	‘Mixture of materials – booklets and online. Flows well. Each week different focus’. (P108)	3
	Easy to read	‘The format of the booklets made them easy to read’. (P132)	4
	Exercises	‘Exercises worked into them’. (P132)	3
	Ability to complete the booklets at home	‘I was able to make this journey on my own in the privacy of my own home and helped to intensify my sense of achievement’. (P150)	1
Barriers to engaging with the booklets	Too much information	‘Some pages I felt had too much on the page – a bit too busy and overwhelming’. (P108)	2
	Lack of guidance or interaction	‘The onus was completely on me’. (P125)	2
	Explanations too technical	‘Some symptoms were a bit technical in the explanations’. (P137).	1
	Difficult to complete the booklets in the timeframe	‘The timeline and pressure to complete the booklets didn't work well for me however that was on me as I had too much going on at the time’. (P132)	1
	Lack of accessibility	‘How would people with visual problems, fine motor skills or learning disabilities access this intervention and what about one for children?’. (P162)	1
	QR codes needed	‘QR codes would've been brilliant as then I could've watched the videos on my phone’. (P162).	2

*Note*: Not all 15 participants provided a qualitative answer to each question.

Abbreviations: P, participant number; PH, pulmonary hypertension.

## DISCUSSION

The primary aim of the study was to test the effectiveness of a CBT self‐help intervention for depression designed specifically for adults with PH. A RCT was used comparing the intervention group to a wait‐list control group. Other aims included assessing change in anxiety, HRQoL, pain and fatigue over time as secondary outcomes; gathering feedback on the acceptability and feasibility of the self‐help booklets and trial; and examining whether unhelpful thoughts and behaviours mediated a change in depression.

The intervention was associated with a significant reduction in depression and HRQoL when compared with the control arm, both with medium effect sizes (*f* = .27 and *f* = .25 respectively). While both the per‐protocol and ITT analyses found a significant reduction in depression over time between groups, a significant improvement in HRQoL was only found in the ITT analyses. We accept our hypothesis that the CBT self‐help booklets would significantly reduce depression in the intervention group compared with the control group at post‐intervention.

Participants in the CBT group reported a significant reduction in depression scores after the intervention and at follow‐up; however, no significant difference was seen at follow‐up compared with post‐intervention. This suggests that while treatment effects were consolidated at follow‐up, symptoms did not continue to reduce after the intervention ended. In other words, while there was no evidence of delayed treatment effects, those who found the intervention beneficial were unlikely to report a relapse at 1 month. Individuals receiving the intervention who fail to report a change in depression scores once treatment has ended may benefit from being stepped up to more intensive approaches to care straight away rather than waiting for delayed effects.

In contrast, no significant difference was reported in HRQoL by those in the CBT group after the intervention, but there was a difference at follow‐up. This means although HRQoL does not change immediately after the intervention, participants experienced an improvement at follow‐up. This finding may be useful to share with individuals receiving the intervention; for example, it may encourage participants to continue using the strategies despite feeling disheartened or unmotivated following a lack of change in their quality of life after the intervention ended.

It is conceivable that a consequence of the alleviation of depression symptoms is to improve HRQoL. However, based on the current data, we are unable to account for the factors responsible or explain why the effect was delayed. While we found a significant interaction between groups over time in unhelpful thoughts and behaviours in the per‐protocol analysis, significance was lost in the ITT analysis. Moreover, the ITT analyses showed involvement in the trial was associated with a significant improvement in thoughts and behaviours in both groups. Change in CBPQ predicted change in depression scores, but change in CBPQ did not significantly mediate the relationship between intervention group and change in depression. This suggests the CBPQ tool was not measuring mechanisms of change associated with the reduction in depression observed. Behavioural activation has been identified as a key driver of change in CBT for depression (López‐López et al., 2019), whereas exposure and anxiety hierarchies form a key part of treatment plans for people with anxiety (Luong et al., 2020). Indeed, in adults with PH, unhelpful behaviours have been found to be a significant predictor of depression, whereas cognitions were not – conversely, cognitions but not behaviours significantly predicted anxiety (Rawlings, Beail, et al., 2022; Rawlings, Thompson, et al., 2022). Questions in the CBPQ relating to cognitions may have introduced measurement noise, meaning it was less accurate in identifying change.

There were no significant differences between the intervention and control groups on the other secondary outcome measures: anxiety, pain, and fatigue. Changes in behaviour due to measurement effects may explain why the control group showed improvements over time. This is sometimes also known as the Hawthorne effect (Abraham et al., 2018). For example, those in the control group reported a significant reduction in depression scores at follow‐up compared with baseline, and significant main effects of time were seen in all secondary outcomes (Table 5). Nevertheless, the control group did not show a significant reduction in depression in comparison to the intervention group. Interestingly, the self‐help intervention for anxiety in PH was associated with significant reductions in anxiety and depression, which were mediated by change in CBPQ. When combined, this fits with the evidence base suggesting components of CBT for depression and anxiety are different, and access to interventions should be informed by primary need. If the person's goal is to manage their anxiety, the self‐help resource for anxiety may be offered, whereas the resource for depression could be offered for those with low mood. For individuals with mixed anxiety and depression, the anxiety resource may still be most relevant, given changes were seen in both mood difficulties in that trial, unlike the current study. However, this warrants future research before being implemented in practice.

The reciprocal relationship between physical and mental health has been recognized for some time; however, it has not always been prioritized in the design of services and treatment for PH (Rawlings et al., 2023). Most participants in this study reported having benefitted from the intervention and valued that the intervention was specific to PH. This suggests participants valued the integration of physical and mental health, with the booklets being designed specifically for depression in PH. This fits with another study demonstrating that unguided self‐help was acceptable for people with PH (Rawlings, Beail, et al., 2022), and other long‐term conditions (Farrand & Woodford, 2015). Participant feedback suggested that the intervention was acceptable; most participants reported that the intervention was helpful for depression and thought that the intervention would be beneficial to individuals following a diagnosis of PH. The self‐help nature of the study means it has the potential to be offered prior to a therapist‐directed treatment, fitting with the ethos of prudent healthcare delivering the minimal amount of treatment required to be effective for the person and achieve their desired outcomes (Addis et al., 2019). This means that some individuals may benefit enough from the self‐help intervention; therefore, more intensive interventions are not required.

However, a small number of participants indicated that they might have benefitted from additional guidance, which is in keeping with the wider literature on self‐help interventions (Cuijpers et al., 2010; Gellatly et al., 2007). Attrition rates can be as high as 50% in self‐help interventions (Webster et al., 2014), compared with 14.7% in this study, with significantly higher attrition in the intervention group compared with the control group. This may have been due to the nature of depression, meaning participants understandably struggled with motivation to complete the self‐help booklets, especially given the unguided nature (Gellatly et al., 2007). These participants may have needed a more intensive individualised intervention than self‐help. However, the attrition rate is also complicated by the nature of PH, resulting in regular hospital visits and stays for some individuals, which may have impacted adherence (Bergot et al., 2019). A separate pilot study examining acceptability may have helped shed further light on this, such as interviewing people who dropped out.

The significantly higher drop‐out rate in the intervention group compared with the control group has been found in other studies of CBT for depression (Hagen et al., 2005; Warmerdam et al., 2008). However, it was not found in a comparable study testing CBT self‐help for anxiety (Rawlings, Beail, et al., 2022). This may be due to different presentations seen in anxiety compared with depression and due to the timeframe of both studies. Rawlings, Beail, et al. (2022) recruited during the coronavirus pandemic when most of the public were at home and unable to socialise due to restrictions. This may have led to increased ability and willingness to complete the self‐help booklets compared with the current study when restrictions were lifted.

Artificial intelligence (AI) is a potential method for building rapport and adherence in self‐help interventions (Gual‐Montolio et al., 2022). Future research could look to explore the feasibility and acceptability of AI in facilitating engagement with self‐help interventions, the impact of digital reminders on adherence (Bevens et al., 2022), and the benefit of expert‐by‐experience accounts of completing such interventions.

Individuals with PH living globally (e.g., Canada, India, UK and Australia) were recruited via Pulmonary Hypertension Association charities with no restrictions on length of time since diagnosis or severity of PH. Recruiting from multiple countries is a strength as it means the sample is more representative of the general population with PH (Choudhary et al., 2013; Frost et al., 2013; Ling et al., 2012). However, there is a need to examine the effectiveness of interventions for depression in other regions as there may be cultural and service differences.

The recruitment target of 77 participants was not met in this study, which is a limitation and suggests a longer recruitment period would be needed for this to be feasible. A longer‐term follow‐up would have also been helpful to evaluate the impact of the intervention over time (Llewellyn‐Bennett et al., 2016). Acceptability and feasibility were built into this study as secondary aims and arguably might have been investigated independently. Despite this, the intervention itself appeared to be feasible based on participant feedback and drop‐out rates. Further studies are needed to assess the impact of CBT for depression in individuals with PH with larger sample sizes and longer follow‐up time periods.

The need to increase our understanding of the experience of this population engaging in psychological treatments has been identified (Rawlings et al., 2023). The sample included mainly females, representing the increased risk of females developing PH compared with males (Austin et al., 2013; Rodriguez‐Arias & García‐Álvarez, 2021); however, it also shows that males were underrepresented in this study. This is unfortunately a common finding in randomised trials targeting depression (Knox et al., 2023). Future research exploring facilitators and barriers to males engaging in depression research in the context of PH would be helpful, such as how to target recruitment strategies.

Despite the study recruiting internationally, the study was not accessible to individuals unable to understand the English language. Evidence suggests engaging in therapy that uses your first acquired language (mother tongue) is a key element that could affect outcomes (Tannenbaum & Har, 2020). It is unknown whether English was the first acquired language of individuals that participated in this study. There are important cultural adaptations that would be useful to consider if the intervention was translated to other languages.

Finally, we grouped individuals living in the UK and outside of the UK despite the groups significantly differing in ethnicity and WHO functional class. The significant difference in WHO functional class is likely to be because 23 participants living in the UK selected ‘not sure’, representing over half of the UK sample. In comparison, 79% of international participants (*n* = 19) categorized their WHO functional class. This significant result appears to represent uncertainty or lack of disclosure from participants living in the UK, rather than a reflection of a differing level of severity of PH (although we cannot rule this out).

In conclusion, this is the first CBT self‐help intervention tailored specifically to depression in PH and investigated using a RCT design. The results showed a significant reduction in depression and significant improvement in HRQoL in the intervention group compared with the control group. The study provides support for the use of CBT for depression in adults with PH. Feedback from participants suggests that tailoring the self‐help booklets to PH was valued. The results were shared with PHA‐UK, and the booklets have been made widely available to people with the condition free of charge.

## AUTHOR CONTRIBUTIONS

**Abbie S. L. Stark:** Conceptualization; writing – original draft; methodology; writing – review and editing; formal analysis; project administration; data curation; resources. **Gregg H. Rawlings:** Conceptualization; writing – original draft; methodology; writing – review and editing; resources; supervision; formal analysis. **James D. Gregory:** Writing – original draft; methodology; supervision. **Iain Armstrong:** Conceptualization; funding acquisition; methodology; writing – review and editing. **Melanie Simmonds‐Buckley:** Formal analysis; writing – review and editing. **Andrew R. Thompson:** Conceptualization; methodology; writing – original draft; writing – review and editing; supervision; resources.

## CONFLICT OF INTEREST STATEMENT

Dr. Rawlings has received payment from Janssen‐Cilag Ltd. for a presentation on depression and PH. The authors have no conflicts of interest.

## Supporting information


Data S1.

Data S2.

Data S3.

Data S4.

Data S5.

Data S6.

Data S7.

## Data Availability

Data can be made available on reasonable request.
