# Supplementary material for: A randomized controlled trial of self‐help cognitive behavioural therapy for depression in adults with pulmonary hypertension
Source: Br J Health Psychol. 2025 Jun 12;30(3):e12800. doi: 10.1111/bjhp.12800 (PMC12159717; doi:10.1111/bjhp.12800)

**Supplementary Materials**

**Supplementary Materials 1: Adherence and acceptability questionnaire at**

**week 2**

**Lead researcher telephones participants in the intervention group**

Hi, my name is [ANONYMISED], can I please speak to {participant’s name}. I am contacting you regarding the study that you are taking part in about the self-management intervention to help individuals with pulmonary hypertension to manage depression. [ANONYMISED] are running this project.

You gave me permission to contact you partway through the study to talk with you about how you are getting on with the intervention.

- Is this a suitable time to speak?

- If no, arrange a suitable time to call if possible
-If yes, this phone call will only be brief and should take no more than 10 minutes. I have some questions to ask you about your experiences of the intervention so far:

- 1. Are you comfortable talking on the phone with me about your experiences of the intervention (i.e., do you have any concerns with confidentiality?).
  2. It is a good idea to take this call in a space that is private and quiet, is that possible? (i.e., are there any distractions and is the participant alone?)
  3. Can I confirm that you have received the intervention?

Yes – move to next question
No – confirm postal address or email address and arrange for intervention to be re-sent

- 1. Have you managed to have a look at the booklet?
     No – explore reasons why
     Yes – how much have you looked at the intervention?

1. (not at all) – 5 (a great deal)
   1. What section are you up to?
   2. How much have you understood the information in the intervention so far?
      1 (not at all) – 5 (a great deal)
   3. How distressing have you found the intervention so far?
      1 (not at all) – 5 (a great deal)

If participants scored 4 or more on item g, the following information will be used:

“I am sorry to hear that you have found the intervention distressing. While it can be normal that focusing more on some of the difficulties associated with depression and PH can cause some distress, we anticipate that this distress will reduce over time. Nevertheless, I would like to remind you that you have the right to stop taking part at any time and you do not have to give a reason. If you would like further support I would also remind you that there is a list of organisations that you might contact contained at the end of each booklet” (The list includes the following contact numbers: 1) Samaritans - <https://www.samaritans.org> or telephone 116 123; 2) Mind - <https://www.mind.org.uk> or telephone 0300 123 3393; 3) Depression UK <https://www.depressionuk.org>; 4) NHS self-help -<https://www.nhs.uk/conditions/stress-anxiety-depression/self-help-therapies/>); 5) 999 emergency services.)

“Can I please confirm that you are happy to continue taking part?” (Yes or no). *If no*, participants will be thanked for their participation and invited to provide a reason for withdrawal. This will then be discussed with members of the research team and if appropriate the Chair of the ethics committee following which, any action points will be taken. *If yes*, the participants will be thanked and reminded that if the distress continues or increases, they should consider stopping the intervention and either contact a member of the research team, their healthcare provider and/or the list of services include at the end of each booklet.

The following statement will then be read: “Before I continue asking you questions, is there anything else you would like to say about the distress you are experiencing?” If the participant discloses anything concerning including thoughts of self-harm or suicide or risk of harm to others or harm from others, the researcher will explain that the participant should stop their participation in the intervention as they will need to speak with their research team and will contact the participant at a later date – they will then be signposted to the services above. The researcher will then discuss this with members of the research team and chair of the ethics if appropriate and any action points will be taken.

- 1. How difficult have you found using the intervention?
     1 (not at all) – 5 (a great deal)
  2. How often have you been using the skills you have developed so far from the intervention?
     1 (not at all) – 5 (a great deal)
  3. How much do you feel the intervention is helping you with your depression?

1 (not at all) – 5 (a great deal)

- 1. How much do you feel the intervention is helping you in other areas of your life?
     1 (not at all) – 5 (a great deal)
  2. How much do you think the intervention can help you with your depression?
     1 (not at all) – 5 (a great deal)
  3. How much do think the intervention can help you in other areas of your life?
     1 (not at all) – 5 (a great deal)
  4. Do you intend to finish the intervention?
     yes or no
  5. Do you have any other comments about the intervention at this stage? (Open ended question)

Thanks for your time. Please remember we will contact you again in 2 weeks via email when you should have completed the intervention asking you to complete some questions.

**Supplementary Materials 2: Final acceptability questionnaire after follow-up**

Dear Participant,

Thank you for taking part in the research study that aimed to develop and evaluate a self-management intervention to help individuals with pulmonary hypertension manage difficulties associated with depression.

To help us to better understand your experiences of using the self-help intervention and taking part in the study, we have created a questionnaire asking you about your experiences that we would like you to answer. This questionnaire should take approximately 10-15 minutes to complete. We are interested in your honest opinion, whether they are positive or negative. Thank you very much, we appreciate your help:

1. What motivated you to take part in this research study? (open ended)
2. How much did the intervention help you to manage your depression?

a. 1(not at all)–5(a lot)

b. Please expand on your answer:

1. How much did the intervention help you in other areas of your life?

a. 1(not at all)–5(a lot)

b. Please expand on your answer:

1. How likely are you to recommend the intervention to another person with pulmonary hypertension?

a. 1(not at all)–5(a lot)

1. Please rate each of the following sections in terms of how helpful it was for managing your depression: 1 (not at all helpful) – 5 (extremely helpful)

Component 1-4

Depression and pulmonary hypertension

Replacing inactivity

Challenging negative thoughts

Keeping well

6. The questionnaires asking about my depression, anxiety quality of life, pain, fatigue and thoughts and behaviours were relevant to my difficulties

a. 1 (strongly disagree) – 5 (strongly agree) b. Please expand:

1. I feel more in control of my depression
   a. 1 (strongly disagree) – 5 (strongly agree)
2. I was given enough time to work through the intervention a. 1 (strongly disagree) – 5 (strongly agree)
3. I valued that the intervention was specific to pulmonary hypertension a. 1 (strongly disagree) – 5 (strongly agree)

10.I think when people are diagnosed with pulmonary hypertension they would benefit from being given this intervention

a. 1 (strongly disagree) – 5 (strongly agree)

11.I think relatives and friends of those with pulmonary hypertension would benefit from using this intervention

a. 1 (strongly disagree) – 5 (strongly agree)

12.I valued being contacted partway through the study by a member of the research team

a. 1 (strongly disagree) – 5 (strongly agree)

13.The level of support I received from the research team was sufficient

a. 1 (strongly disagree) – 5 (strongly agree) 14. Please expand on your answer

15.I found the letter writing exercise in booklet four useful

a. 1 (strongly disagree) – 5 (strongly agree)

b. I give consent for the research team to use the contents of my letter for research purposes. I understand the information will be used to support other research in the future, and may be shared anonymously with other individuals, for example, for research publications. I give permission that the research team may use this information in publications, reports and other research outputs, with the confidentiality of my information preserved. This means that no information will be included that makes me identifiable: Yes or No

16.How do you plan to continue using the skills you have developed by using the intervention (open ended):

17.Overall, how would you rate the self-help intervention? a. 1(Poor)–5(Excellent)
b. Please expand on your answer:

18.What did you like the most about the intervention? (open ended) 19.What did you like the least about the intervention? (open ended)

20.Do you have any other comments about the intervention at this stage? (open ended question)

Thank you for your time and participation in the study.

**Supplementary Materials 3: Selected pages from the self-help resource**

All front covers are cropped for anonymisation purposes to remove author information and funding details.

Booklet 1 front cover and example page


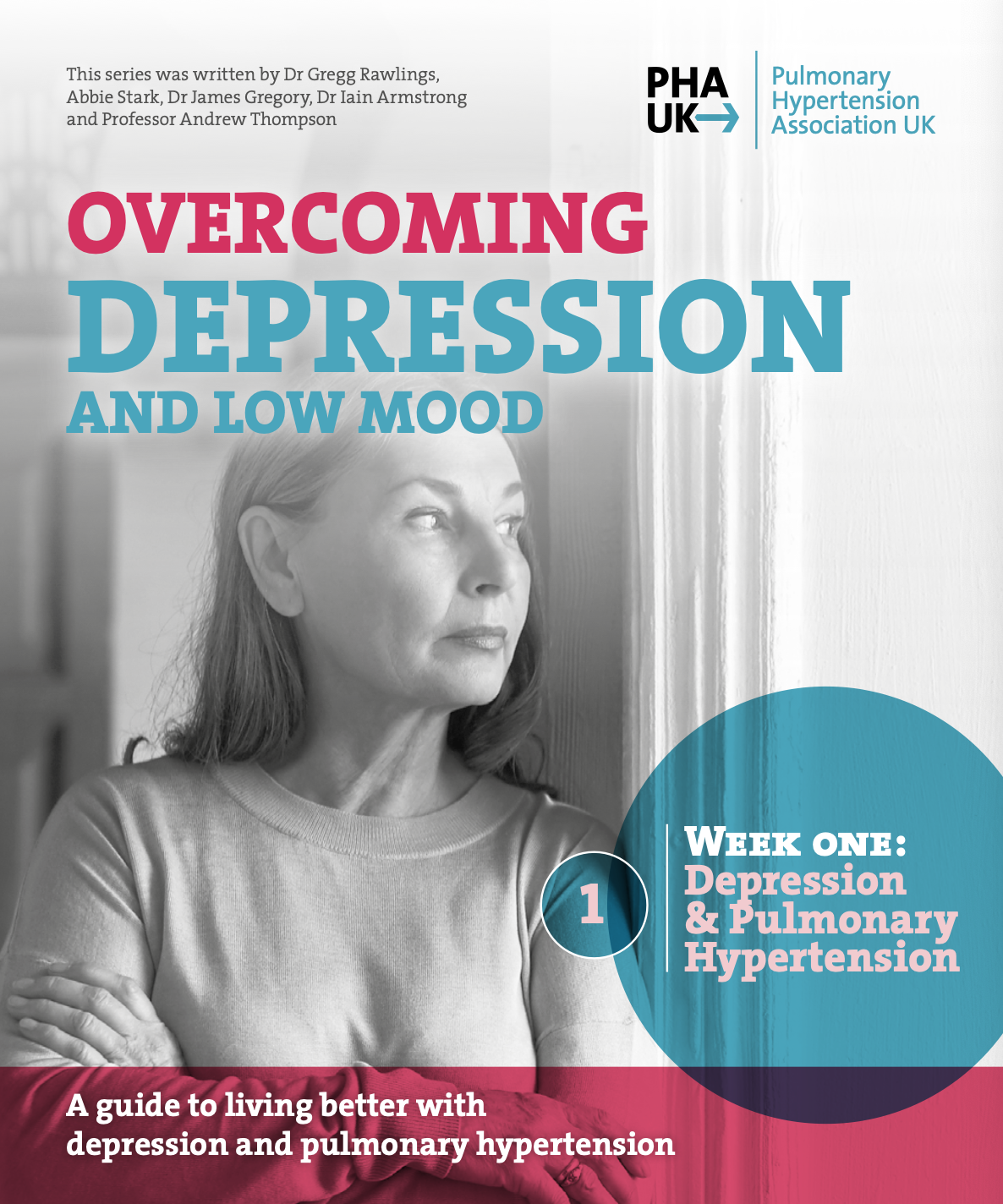


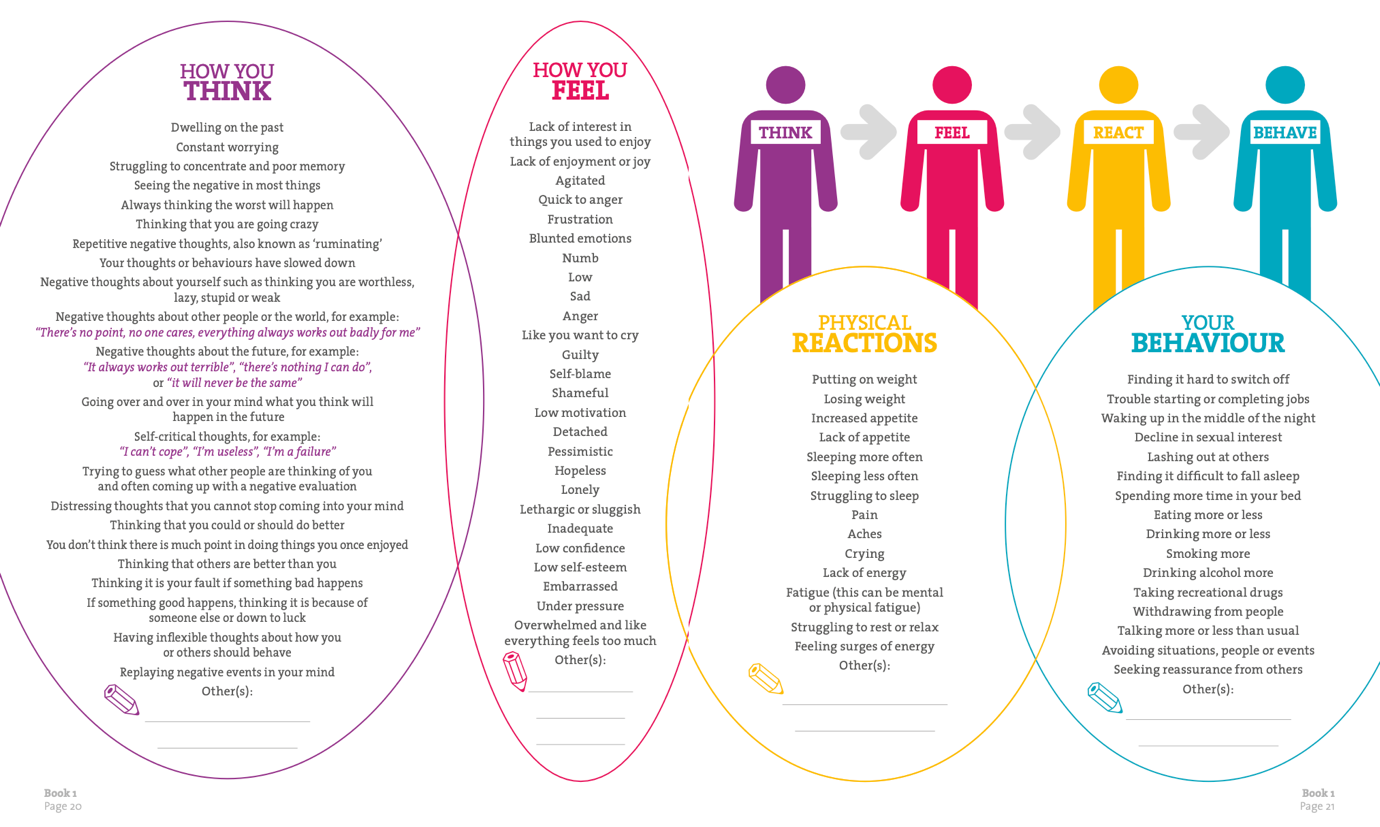

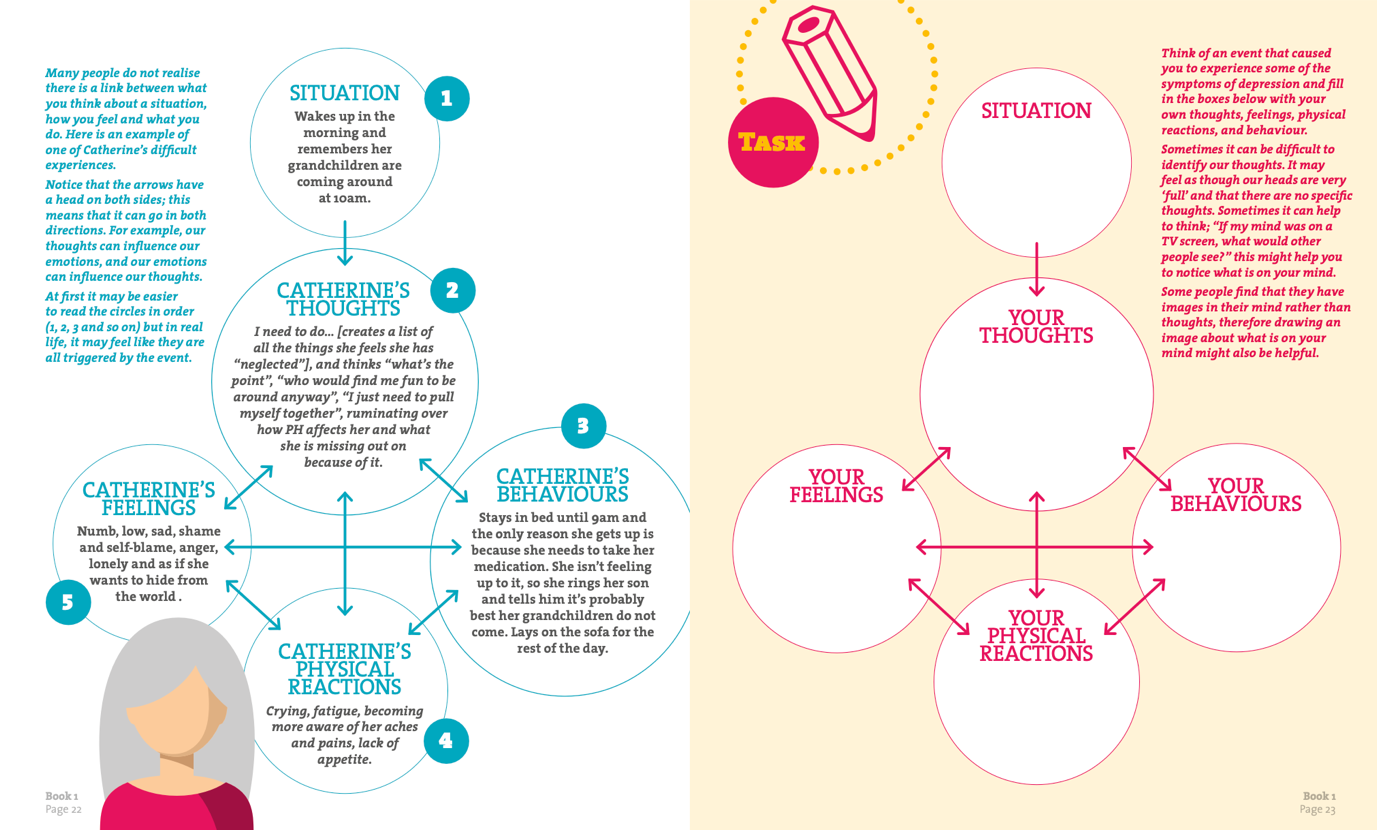


Booklet 2 front cover


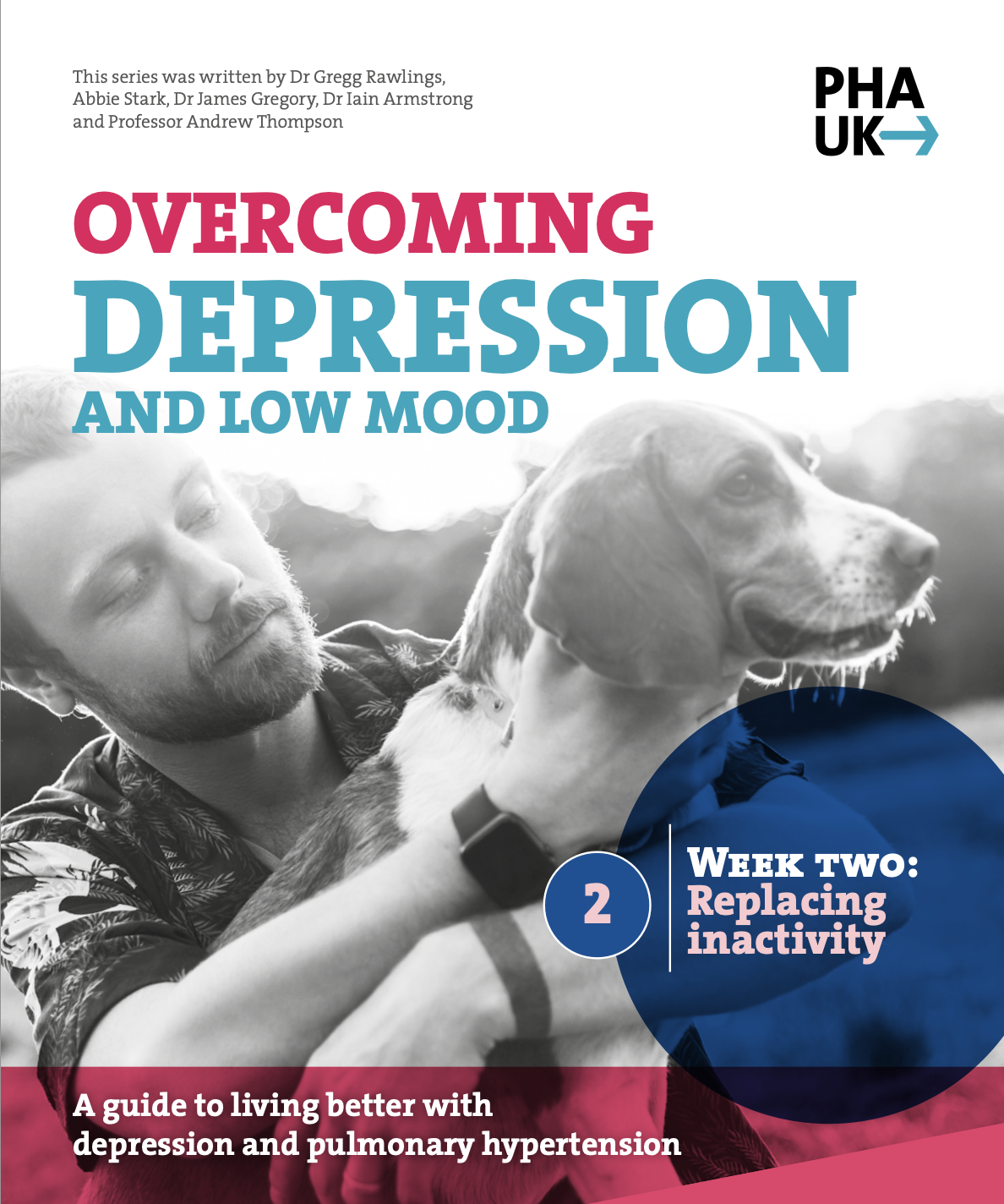


Booklet 3 front cover and an example page


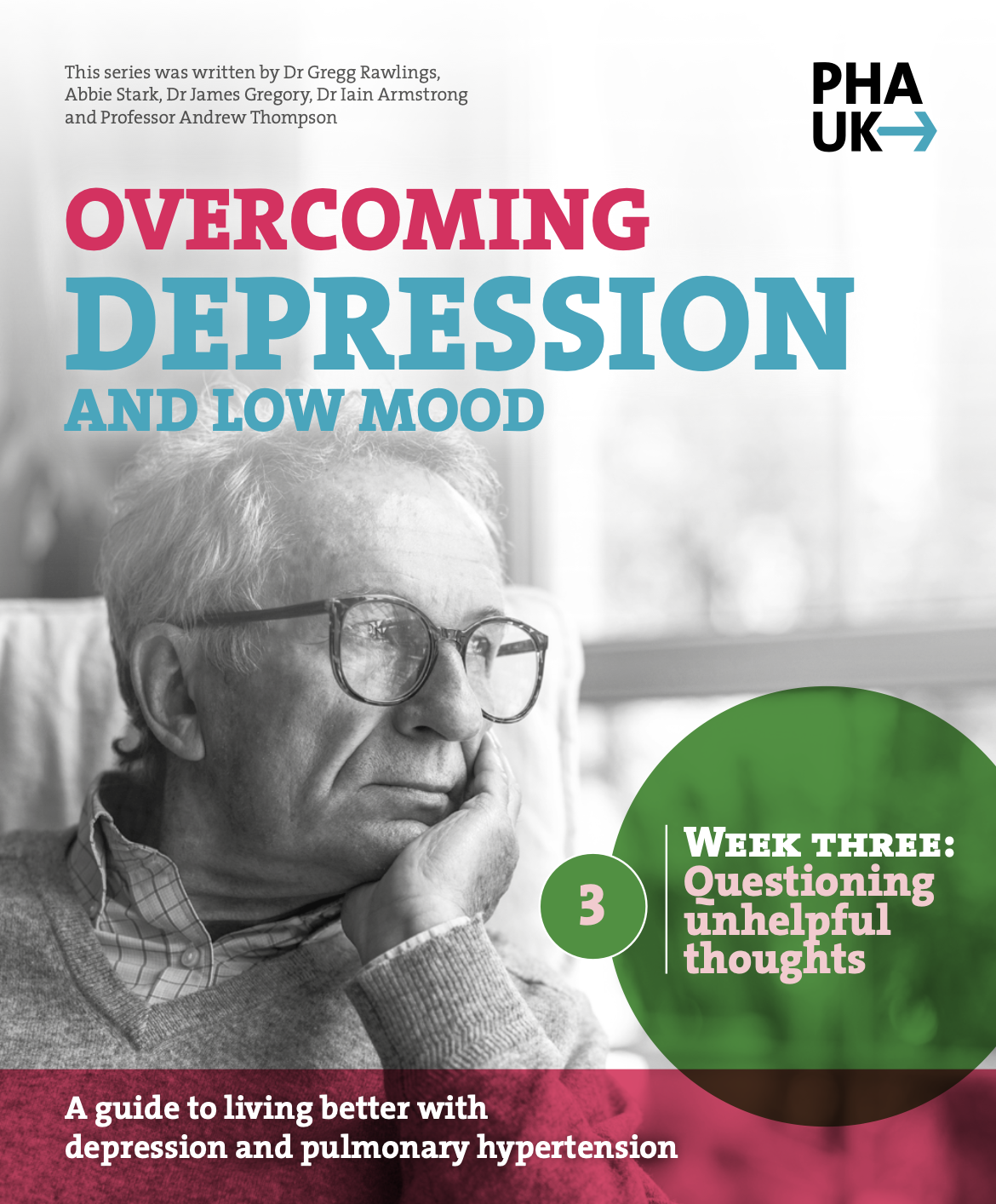


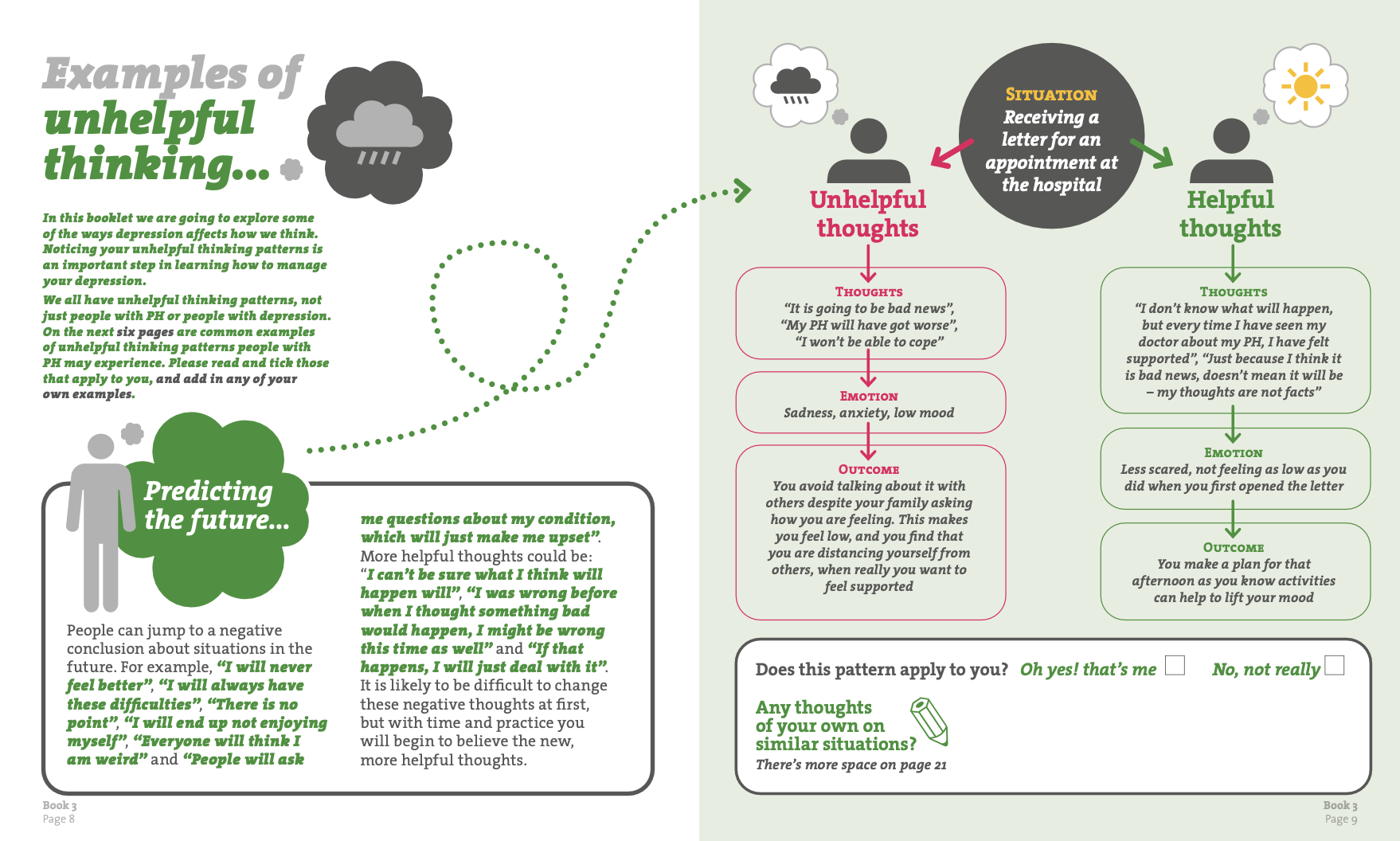


Booklet 4 front cover and an example page


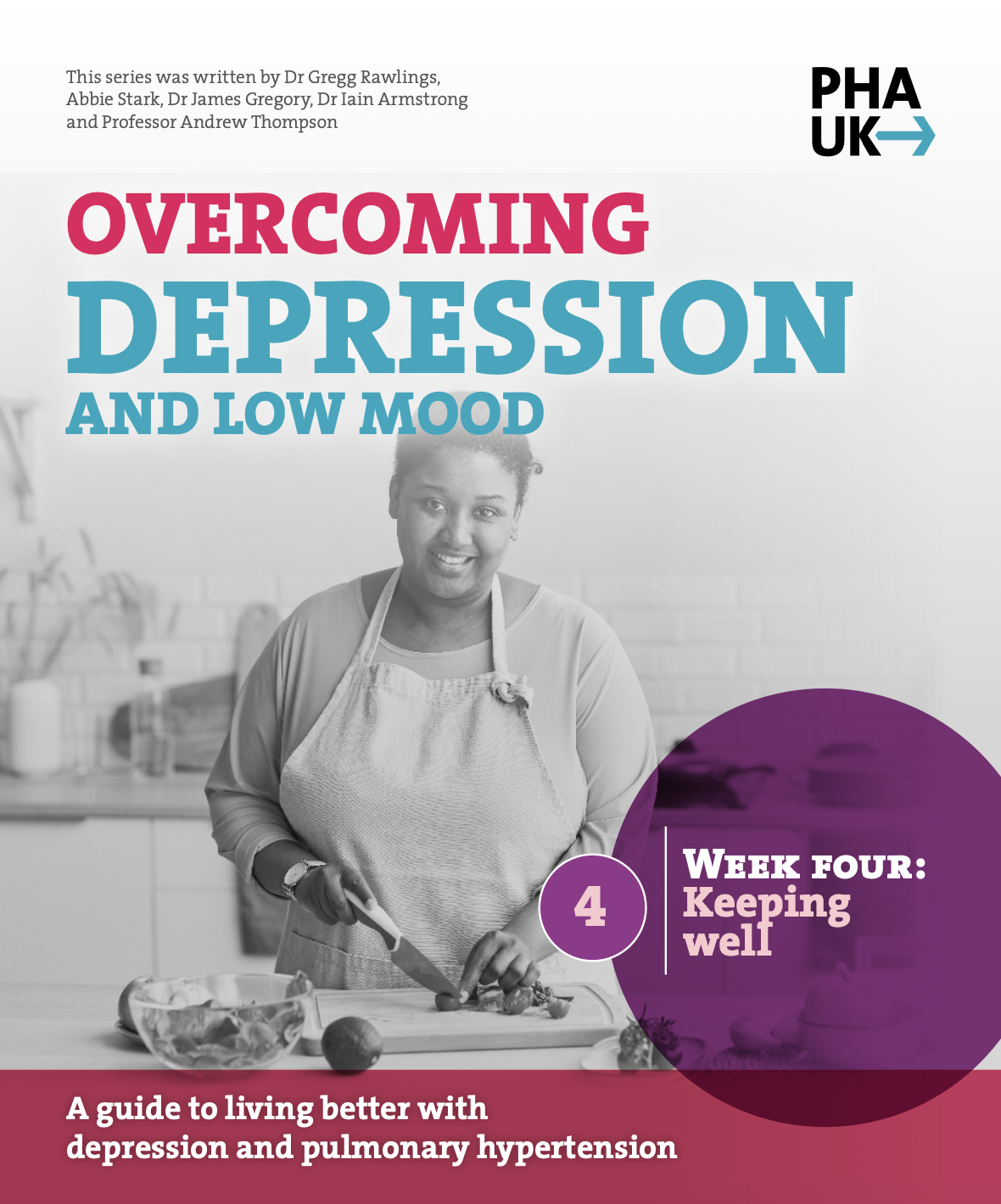


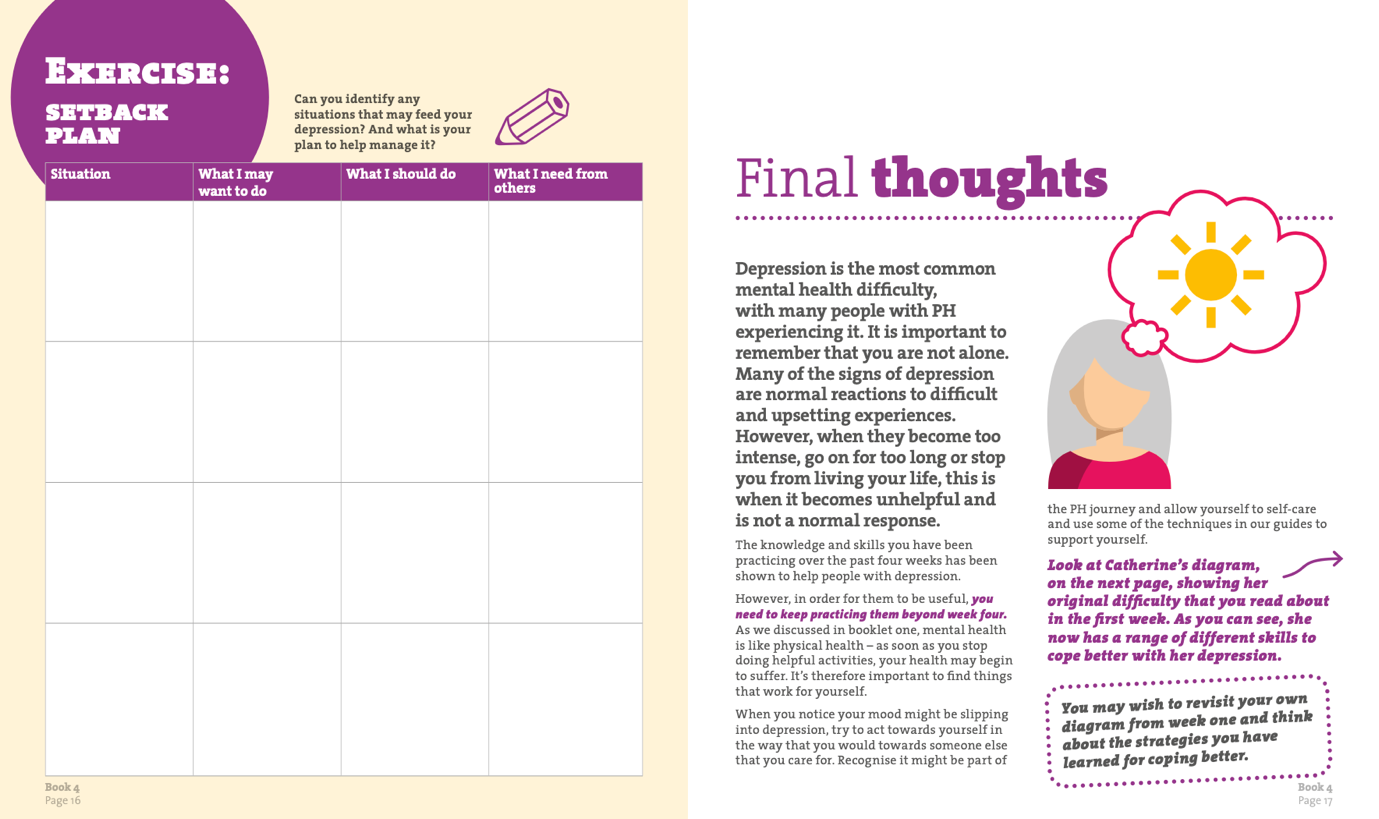


**Supplementary Table 1a: Results from per-protocol analysis**

*Means and standard deviations in the intervention (n=22) and control (n=33) group for primary and secondary outcome measures, including results of 2x3 mixed ANOVAs.*

| Outcome measure | Group | Baseline | Post | Follow-up (one-month) | Two-way mixed ANOVA  (group x time)  F | *p* | η2 | Cohens’ f |
| --- | --- | --- | --- | --- | --- | --- | --- | --- |
| Depression | CBT Control | 13.57 (4.81) 13.21 (5.57) | 9.67 (5.03) 11.91 (6.18) | 8.48 (4.32) 11.12 (6.18) | F (1.745, 90.757) = 3.503 GG | 0.040* | 0.063 | 0.26 |
| Anxiety | CBT Control | 10.10 (5.66) 11.30 (5.76) | 7.52 (5.56)  10.52(6.10) | 6.14(4.76)  9.21(6.73) | F (1.720, 89.426) = 1.554 GG | 0.219 | 0.029 | 0.17 |
| HRQoL | CBT Control | 33.10 (8.78)  31.33 (9.94) | 32.05 (8.96) 30.27 (11.89) | 29.52 (9.62) 30.70 (11.76) | F (2, 104) = 2.289 | 0.106 | 0.042 | 0.21 |
| Pain | CBT Control | 29.38 (9.93) 30.68 (14.16) | 33.62 (10.25) ^†^ 32.61 (14.67) | 34.71 (12.00)  34.58 (12.37) | F (1.757, 87.842) = 0.352 GG | 0.677 | 0.007 | 0.08 |
| Fatigue | CBT Control | 54.71 (9.63)  50.16 (12.13) | 51.14 (13.50)  49.03 (11.30) | 47.81 (11.74)  47.06 (13.97) | F (2,102) = 1.717 | 0.185 | 0.033 | 0.18 |
| CBPQ | CBT Control | 73.76 (13.18)  70.25 (16.67) | 57.71 (14.70) ^†^  66.94 (23.76) | 51.00 (14.77)  60.69 (25.19) | F (1.518, 77.400) = 4.674 GG | 0.020* | 0.084 | 0.30 |

Note. * = statistically significant p < .05; ^†^ = Levene’s Test of Equality not met (p < .05); GG = Greenhouse Geisser correction used; HRQoL = Health-Related Quality of Life; CBPQ= Cognitive and Behavioural Processes Questionnaire.

**Supplementary Table 1b**

*Pairwise comparisons using Bonferroni correction for significant interactions between TimexGroup.*

|  |  | Pairwise Comparisons | | |
| --- | --- | --- | --- | --- |
| Outcome measure | Group | Baseline vs Post | Baseline vs. Follow-up | Post vs. Follow-up |
| Depression | CBT Control | *p=*0.001* *p*=0.333 | *p=*0.001* *p*=0.058 | *p=*0.379 *p*=0.609 |
| CBPQ | CBT  Control | *p*=0.001*  *p*=0.924 | *p=*0.001*  *p*=0.038 | *p=*0.001*  *p*=0.015* |

Note. *Statistically significant as p ≤ 0.017.

**Supplementary Table 1c**

*Pairwise Comparisons using Bonferroni correction for significant main effect of Time.*

|  | Pairwise Comparisons | | |
| --- | --- | --- | --- |
| Outcome measure | Baseline vs. Post | Baseline vs. Follow-up | Post vs. Follow-up |
| Anxiety | *p*=0.014* | *p*=<0.001* | *p*=0.031 |
| HRQoL | *p*=0.599 | *p*=0.034 | *p*=0.534 |
| Pain | *p*=0.151 | *p*=0.009* | *p*=0.512 |
| Fatigue | *p*=0.041 | *p*<0.001* | *p*=0.054 |

Note. *Statistically significant as p ≤ 0.017.

**Supplementary Table 2**

*Baseline mean and standard deviation data for participants currently living in the UK versus participants living outside of the UK.*

| Characteristics | UK | International | *p* value |
| --- | --- | --- | --- |
| Number of participants | n=44 | n=24 |  |
| Condition |  |  |  |
| CBT intervention | n=21 | n=12 |  |
| Control | n=23 | n=12 |  |
| Age | 52.95 (12.14) | 51.63 (14.33) | 0.69 |
| Gender |  |  | 1.00 |
| Male | n=37 | n-21 |  |
| Female | n=7 | n=3 |  |
| Ethnicity |  |  | 0.049* |
| White | n=38 | n=16 |  |
| Asian or Asian British | n=3 | n=2 |  |
| Black, Black British, Caribbean or African | n=1 | n=0 |  |
| Mixed or multiple ethnic groups | n=0 | n=3 |  |
| Other ethnic groups | n=0 | n=0 |  |
| Unknown | n=2 | n=3 |  |
| Education (years) | 14.97 (4.65) | 16.42 (5.89) | 0.32 |
| Employment |  |  | 0.67 |
| Full time | n=13 | n=8 |  |
| Part time | n=5 | n=3 |  |
| Not employed | n=9 | n=7 |  |
| Retired | n=17 | n=6 |  |
| PH Type |  |  | 0.66 |
| Idiopathic PH | n=15 | n=7 |  |
| Connective tissue disease | n=4 | n=2 |  |
| Chronic thromboemblic PH | n=11 | n=4 |  |
| Familial PH | n=0 | n=1 |  |
| Congenital PH | n=5 | n=2 |  |
| Other or not sure | n=9 | n=8 |  |
| PH Class |  |  | 0.02* |
| I | n=2 | n=4 |  |
| II | n=10 | n=4 |  |
| III | n=8 | n=8 |  |
| IV | n=1 | n=3 |  |
| Not sure | n=23 | n=5 |  |
| Number of years since diagnosis | 8.11 (8.83) | 11.20 (13.39) | 0.26 |
| Prescribed medication for depression | n=21 (47.73%) | n=13 (54.17%) | 0.80 |
| Received therapy for depression | n=9 (20.45%) | n=7 (29.17%) | 0.55 |
| Depression | 13.32 (5.43) | 12.88 (5.25) | 0.75 |
| Anxiety | 10.66 (6.12) | 9.54 (5.51) | 0.46 |
| HRQoL | 30.86 (9.44) | 31.67 (9.50) | 0.74 |
| Pain | 28.48 (13.51) | 31.96 (12.80) | 0.31 |
| Fatigue | 51.45 (11.24) | 52.25 (11.70) | 0.78 |
| Cognitions and Behaviours (CBPQ) | 71.45 (16.52) | 67.92 (19.14) | 0.43 |

Note. n = number of participants; * = statistically significant p < .05; PH = Pulmonary Hypertension; HRQoL = Health-Related Quality of Life; CBPQ= Cognitive and Behavioural Processes Questionnaire; Prescribed medication for depression within the last 12 months; Received therapy for depression within the last 12 months.

**Supplementary Table 3**

*Baseline mean and standard deviation data for participants who remained in the study versus participants who dropped out.*

| Characteristics | Participants remaining in the study | Participants who dropped out | *p* value |
| --- | --- | --- | --- |
| Number of participants | n= 58 | n= 10 |  |
| Condition |  |  | 0.04* |
| CBT intervention | n=25 | n=8 |  |
| Control | n=33 | n=2 |  |
| Age | 51.71 (12.31) | 57.00 (15.64) | 0.23 |
| Gender |  |  | 1.00 |
|  | n=9 | n=1 |  |
| Female | n=49 | n=9 |  |
| Ethnicity |  |  | 0.59 |
| White | n=46 | n=8 |  |
| Asian or Asian British | n=4 | n=1 |  |
| Black, Black British, Caribbean or African | n=1 | n=0 |  |
| Mixed or multiple ethnic groups | n=2 | n=1 |  |
| Other ethnic groups | n=0 | n=0 |  |
| Unknown | n=5 | n=0 |  |
| Education (years) | 15.13 (4.58) | 17.22 (7.36) | 0.26 |
| Employment |  |  | 0.08 |
| Full time | n=19 | n=2 |  |
| Part time | n=6 | n=2 |  |
| Not employed | n=16 | n=0 |  |
| Retired | n=17 | n=6 |  |
| PH Type |  |  | 0.56 |
| Idiopathic PH | n=18 | n=4 |  |
| Connective tissue disease | n=4 | n=2 |  |
| Chronic thromboemblic PH | n=13 | n=2 |  |
| Familial PH | n=1 | n=0 |  |
| Congenital PH | n=6 | n=1 |  |
| Other or not sure | n=16 | n=1 |  |
| PH functional Class |  |  | 0.26 |
| I | n=5 | n=1 |  |
| II | n=11 | n=3 |  |
| III | n=16 | n=0 |  |
| IV | n=3 | n=1 |  |
| Not sure | n=23 | n=5 |  |
| Number of years since diagnosis | 9.26 (10.50) | 8.88 (12.21) | 0.92 |
| Prescribed medication for depression | n=29 (50.88%) | n=5 (45.46%) | 1.00 |
| Received therapy for depression within last 12 months | n=16 (28%) | n=0 | 0.10 |
| Depression | 13.05 (5.42) | 13.80 (4.96) | 0.69 |
| Anxiety | 10.91 (5.70) | 6.50 (5.89) | 0.03* |
| HRQoL | 31.78 (9.25) | 27.50 (9.89) | 0.19 |
| Pain | 29.05 (13.06) | 33.50 (14.57) | 0.33 |
| Fatigue | 52.16 (11.17) | 49.30 (12.55) | 0.51 |
| Cognitions and Behaviours (CBPQ) | 71.64 (15.68) | 61.90 (24.76) | 0.10 |

Note. * = statistically significant p < .05, PH = Pulmonary Hypertension; HRQoL = Health-Related Quality of Life, CBP-Q= Cognitive and Behavioural Processes Questionnaire; Prescribed medication for depression within the last 12 months; Received therapy for depression within the last 12 months.

**Supplementary Table 4: Acceptability findings at week two.**

| Question | None at all / A little | Moderate amount | A great deal /  A lot | Total n |
| --- | --- | --- | --- | --- |
| How much have you looked at the intervention? | 5 | 6 | 16 | 27 |
| How much of the intervention have you understood? | 2 | 0 | 24 | 26 |
| How distressing is the intervention? | 18 | 5 | 4 | 27 |
| How difficult is the intervention? | 2 | 6 | 18 | 26 |
| How often have you been using the skills? | 8 | 8 | 10 | 26 |
| How much is the intervention helping with your depression? | 6 | 12 | 8 | 26 |
| How much can the intervention help you with your depression? | 4 | 5 | 17 | 26 |
| How much is the intervention helping in other areas of your life? | 8 | 8 | 10 | 26 |
| How much can the intervention help in other areas of your life? | 5 | 4 | 17 | 26 |

Note. Values represent N.

| **Supplementary Table 5: Final acceptability questionnaire quantitative findings.**  *Intervention Feedback Ratings (N=15). The table shows the number of participants selecting each rating, grouped from ratings of 1-2, 3, or 4-5.* | | | |
| --- | --- | --- | --- |
|  | 1-2  Not at all / A little | 3 A moderate amount | 4-5 A lot / A great deal |
| How much did the intervention help you manage your depression? | 3 | 4 | 8 |
| How much did the intervention help you in other areas of your life? | 3 | 5 | 7 |
| How likely are you to recommend the intervention to another person with pulmonary hypertension? | 2 | 2 | 11 |
|  | 1-2 Not at all helpful / Slightly unhelpful | 3 Somewhat helpful | 4-5 Very helpful / Extremely |
| Booklet 1 - Depression and Pulmonary Hypertension | 2 | 4 | 9 |
| Booklet 2 – Replacing Inactivity | 3 | 1 | 11 |
| Booklet 3 – Questioning Unhelpful Thoughts | 4 | 0 | 11 |
| Booklet 4 – Keeping Well | 3 | 2 | 10 |
|  | 1-2  Strongly disagree / Somewhat disagree | 3 Neither agree nor disagree | 4-5 Somewhat agree / Strongly agree |
| The questionnaires asking about my depression, anxiety quality of life, pain, fatigue and thoughts and behaviours were relevant to my difficulties. | 2 | 2 | 11 |
| I feel more in control of my depression. | 2 | 5 | 8 |
| I was given enough time to work through the intervention. | 4 | 4 | 7 |
| I valued that the intervention was specific to pulmonary hypertension. | 1 | 1 | 13 |
| I think when people are diagnosed with pulmonary hypertension they would benefit from being given this intervention. | 1 | 2 | 12 |
| I think relatives and friends of those with pulmonary hypertension would benefit from using this intervention. | 2 | 5 | 8 |
| I valued being contacted partway through the study by a member of the research team. | 2 | 4 | 9 |
| The level of support I received from the research team was sufficient. | 4 | 3 | 8 |
| I found the letter writing exercise in booklet 4 useful. | 6 | 2 | 7 |
|  | 1-2 | 3 | 4-5 |
| Overall, how would you rate the self-help intervention, from 1 (poor) – 5 (excellent)? | 2 | 2 | 11 |

Note. Values represent n.

**Supplementary Figure 1: Clinical change for depression**

*Mean PHQ-8 scores at baseline, post-intervention and follow-up for the CBT and control group including the completer analysis and intent to treat (ITT).*


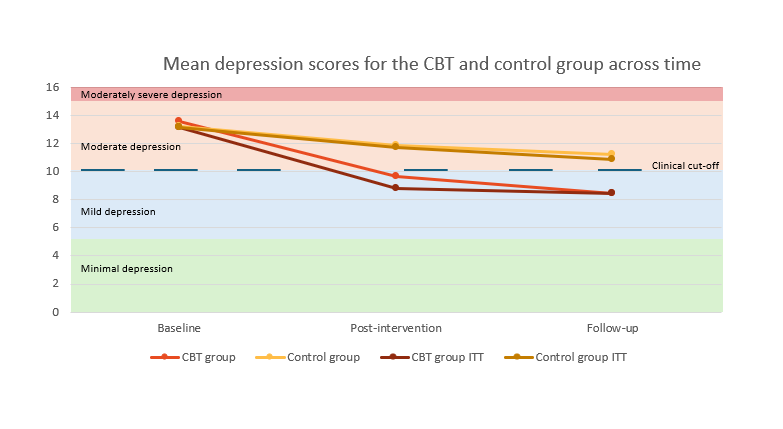


**Supplementary Figure 2: Clinical change for anxiety**

*Mean GAD-7 scores at baseline, post-intervention and follow-up for the CBT and control group including the completer analysis and intent to treat (ITT).*


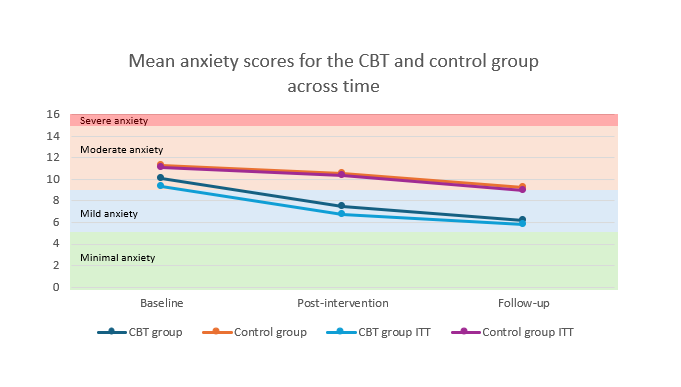

Supplement: Supplementary file 1 — Data S1. [file BJHP-30-0-s004.docx]
