# Supplementary material for: A randomized controlled trial of self‐help cognitive behavioural therapy for depression in adults with pulmonary hypertension
Source: Br J Health Psychol. 2025 Jun 12;30(3):e12800. doi: 10.1111/bjhp.12800 (PMC12159717; doi:10.1111/bjhp.12800)
Supplement: Supplementary file 2 — Data S2. [file BJHP-30-0-s007.pptx]

## Slide 1
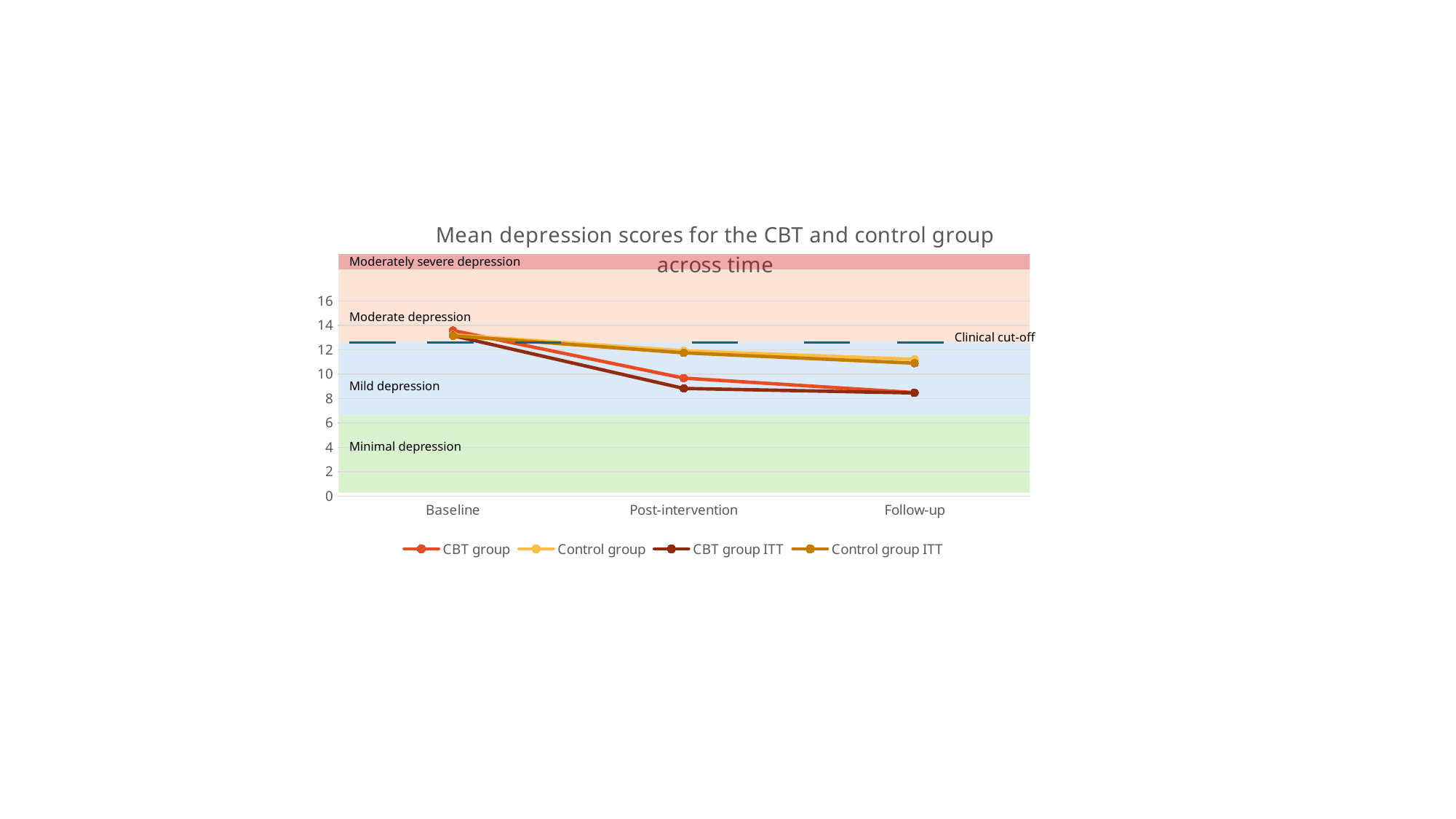

### Chart: Mean depression scores for the CBT and control group across time
| Category | CBT group | Control group | | CBT group ITT | Control group ITT |
|---|---|---|---|---|---|
| Baseline | 13.57 | 13.21 | None | 13.15 | 13.14 |
| Post-intervention | 9.67 | 11.91 | None | 8.82 | 11.74 |
| Follow-up | 8.48 | 11.21 | None | 8.45 | 10.89 |Moderately severe depression
Moderate depression
Clinical cut-off
Mild depression
Minimal depression
