# Supplementary material for: A randomized controlled trial of self‐help cognitive behavioural therapy for depression in adults with pulmonary hypertension
Source: Br J Health Psychol. 2025 Jun 12;30(3):e12800. doi: 10.1111/bjhp.12800 (PMC12159717; doi:10.1111/bjhp.12800)
Supplement: Supplementary file 3 — Data S3. [file BJHP-30-0-s005.pptx]

## Slide 1
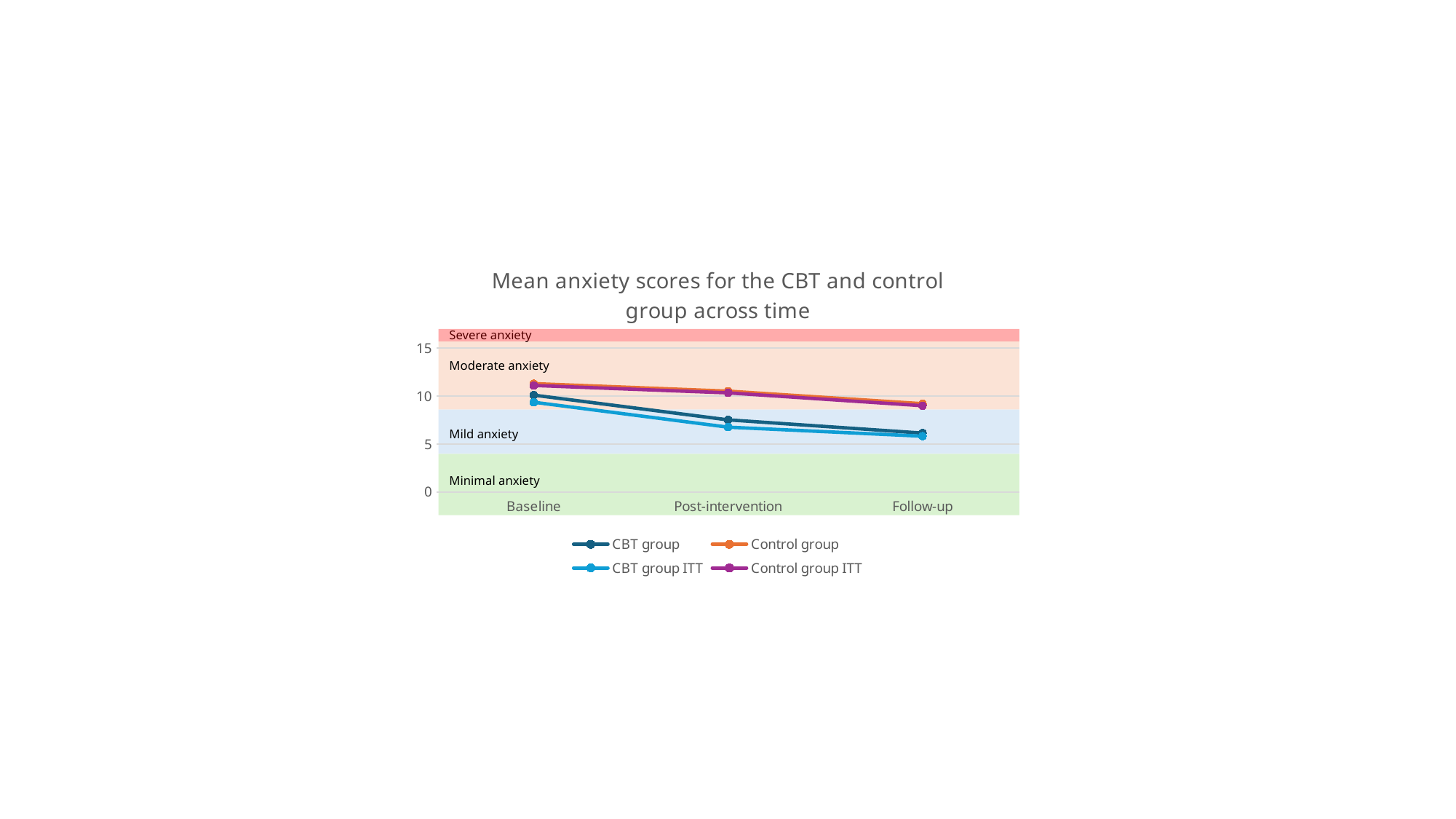

### Chart: Mean anxiety scores for the CBT and control group across time
| Category | CBT group | Control group | | CBT group ITT | Control group ITT |
|---|---|---|---|---|---|
| Baseline | 10.1 | 11.3 | None | 9.36 | 11.11 |
| Post-intervention | 7.52 | 10.52 | None | 6.76 | 10.34 |
| Follow-up | 6.14 | 9.21 | None | 5.82 | 9.0 |Severe anxiety
Moderate anxiety
Mild anxiety
Minimal anxiety
