# Supplementary material for: A randomized controlled trial of self‐help cognitive behavioural therapy for depression in adults with pulmonary hypertension
Source: Br J Health Psychol. 2025 Jun 12;30(3):e12800. doi: 10.1111/bjhp.12800 (PMC12159717; doi:10.1111/bjhp.12800)
Supplement: Supplementary file 4 — Data S4. [file BJHP-30-0-s003.pdf]

This series was written by Dr Gregg Rawlings, Abbie Stark, Dr James Gregory, Dr Iain Armstrong and Professor Andrew Thompson

PHA UK
Pulmonary Hypertension Association UK

OVERCOMING DEPRESSION AND LOW MOOD

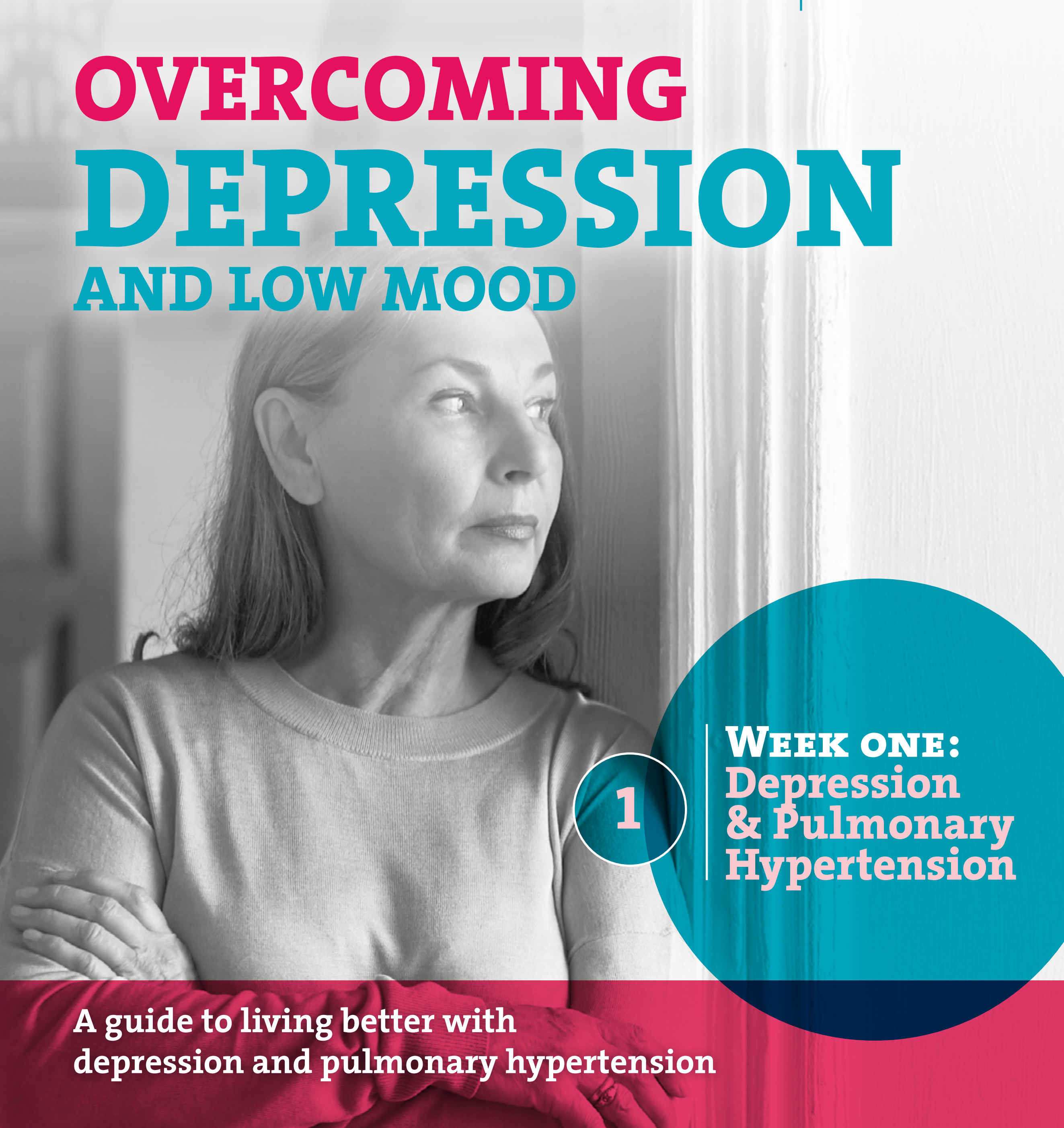

1

Week one: Depression & Pulmonary Hypertension

A guide to living better with depression and pulmonary hypertension

PHA UK

Week one:
Depression & Pulmonary Hypertension

Overcoming depression and low mood

Here is a summary of what will be covered in this booklet:

- *An introduction to depression and low mood in people living with pulmonary hypertension, and how symptoms for these conditions can overlap*
- *An explanation of how to use these booklets*
- *How depression can influence our thoughts, feelings, physical sensations (what we feel in our bodies) and behaviour*
- *Noticing your own thoughts, feelings, physical sensations, and behaviour*
- *How thoughts and behaviours can maintain low mood and depression*

We would like to thank the PHA UK members who contributed their personal experiences to aid the development of these booklets.

This series was written by Dr Gregg Rawlings, Abbie Stark, Dr James Gregory, Dr Iain Armstrong and Professor Andrew Thompson. This series of four booklets has been produced in collaboration with Nottingham Trent University, Cardiff and Vale University Health Board and Pulmonary Hypertension Association UK (PHA UK).

Introduction

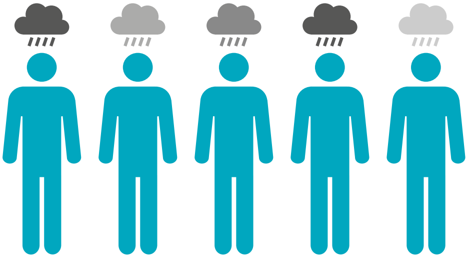

Depression is a common mental health problem with approximately 1.5 million people believed to experience it in the United Kingdom.

People living with long-term health conditions, such as pulmonary hypertension (PH), are more likely to experience depression and other difficulties with their mental health than the general population. In fact, one third of people with PH experience difficulties with depression. In addition, lower levels of depression-like feelings, what we might call 'low mood', are commonly experienced by people living with long-term conditions such as PH.

So, remember, you're not alone.

Depression in people with PH may be associated with a range of experiences such as the impact of PH symptoms; the effect PH has on them and their family and friends; concerns about the future; hospital appointments; medication; how the condition will progress; other people's reactions to the condition; and other challenges such as financial problems, sex and relationship difficulties, and travel restrictions. And that is just to name a few of the issues commonly encountered by people with PH.

As we will see later, depression and low mood can also impact upon energy levels, sleep, concentration and memory, and appetite. It can have a wider impact on daily functioning. Further, we know that depression often co-occurs with anxiety, with six out of ten people living with depression also experiencing anxiety.

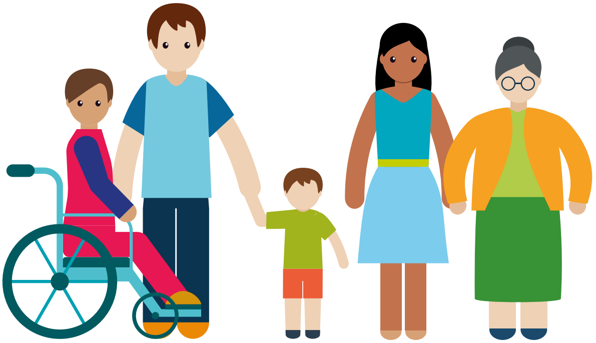

Depression can also make some difficulties that people with PH often experience worse. As shown below, there is an overlap between symptoms of depression and symptoms of PH. For example, some people may experience problems with fatigue due to their PH, but may also experience additional fatigue as a result of depression:

- Fatigue
- Aches and pain
- Reduced physical activity
- Increased risk of anxiety
- Poor memory and concentration
- Feeling isolated

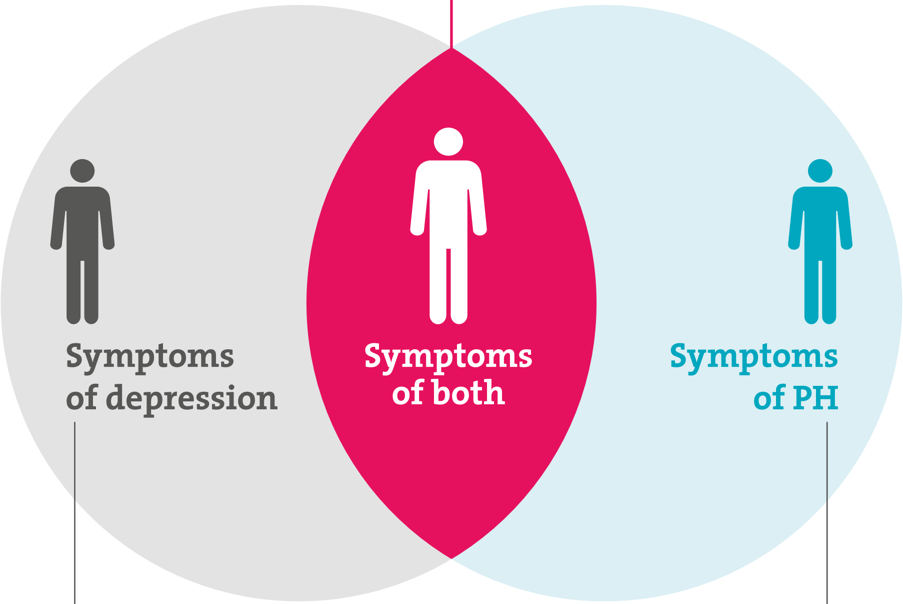

Symptoms of depression

- Lack of interest
- Feeling down or depressed
- Sleeping too much or too little
- Reduced appetite or overeating
- Social withdrawal

Symptoms of PH

- Breathing difficulties
- Swelling
- More difficult to do your usual activities
- Coughing
- Bloating
- Weight gain

Experiences related to depression or anxiety are not always related to people's demographics, such as their age and gender. This means that anyone with PH can experience difficulties with their mood and it is not just isolated to one group.

Dr Gregg H. Rawlings
Nottingham Trent University
Developer of *A guide to living better with depression and pulmonary hypertension* and *A guide to living better with anxiety, worry and panic in pulmonary hypertension*

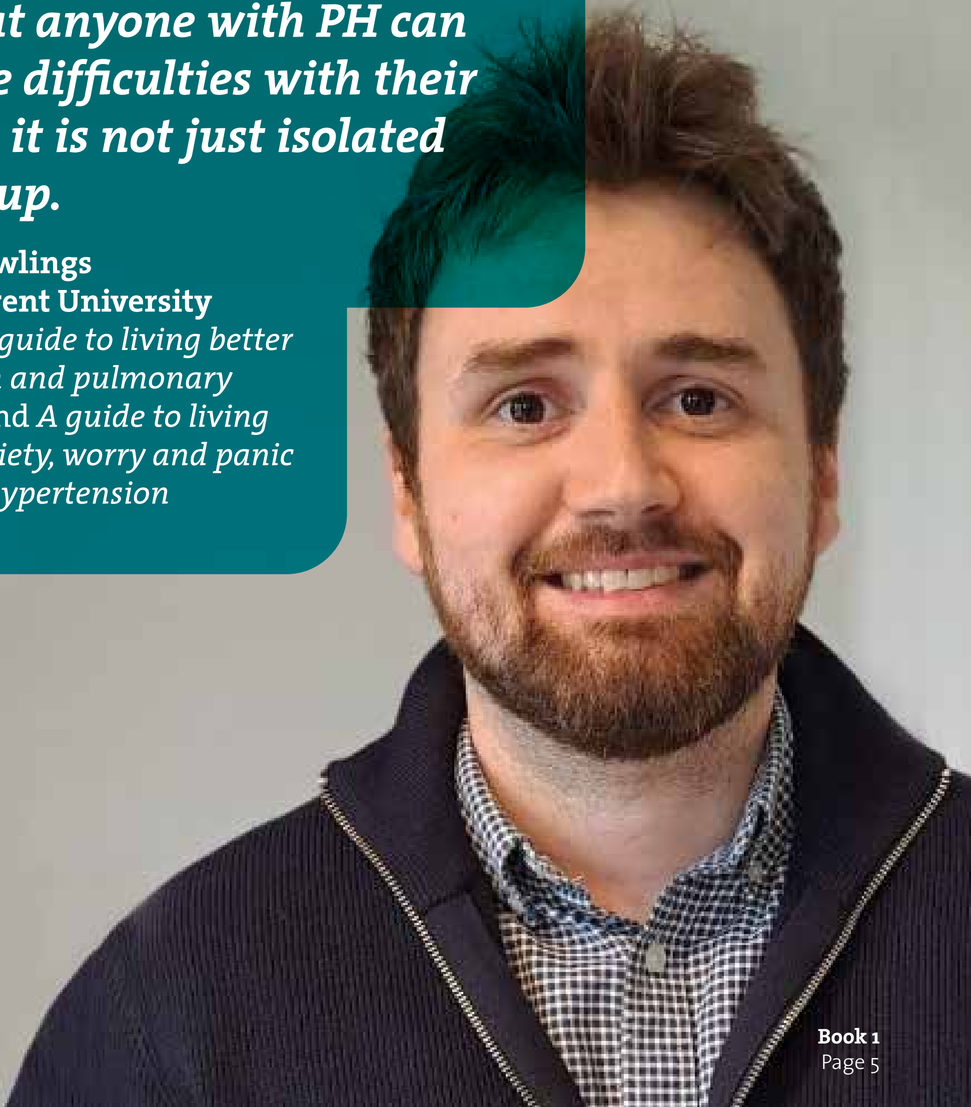

Introducing *Catherine* & *Ally*

Throughout this series of booklets, to help illustrate and explain different strategies, we will follow the stories of Catherine and Ally, who both have a diagnosis of PH and who both experience depression, but are affected by it in different ways. While both Catherine and Ally are made up, the difficulties they experience are commonly reported by real people with PH. Perhaps you will recognise some of their difficulties in yourself.

Catherine is a 69-year-old female who developed depression soon after she was diagnosed with PH seven months ago. Although there have been times in her life when she has felt low, this time she feels worse. Catherine cannot stop thinking about her future and is having negative and distressing thoughts, such as ***"Nothing good will happen again"****.*

Although her doctors have reassured her that she is responding well to the medicine, Catherine cannot stop thinking about her PH. She struggles to sleep as she lies awake with repetitive negative thoughts about what she cannot do that she was able to do before developing PH. In the morning, she struggles to get out of bed because she hasn't slept very well, but also, she doesn't see the point in getting up.

Catherine has always loved spending time with her grandchildren but now feels guilty about not being able to run and play with them like she used to. Although she doesn't enjoy food in the same way, she is eating more and has put on weight, which she is self-conscious about. She struggles to engage in physical activity as she is anxious about becoming breathless. Catherine has noticed she has little motivation to do anything, even things she used to enjoy.

Catherine often feels annoyed at herself if she does not do things she feels she should, such as getting up by a certain time, cleaning her house, and speaking to her friends. She tells herself to ***"pull herself together"*** *and stop being so* ***"useless"****. She has always seen herself as a young person; however, now she feels as if she is grieving for her old body.*

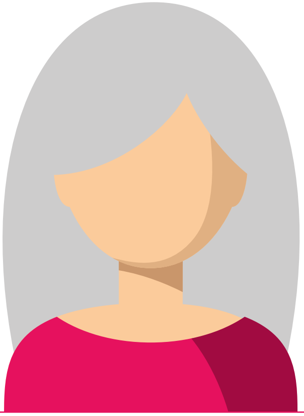

Ally is a 25-year-old male. He has lived with PH for a number of years and felt he was coping well, but just as his friends his age are gaining more independence, he is upset that he cannot do the things they are.

The medication he must regularly take is almost a constant reminder of his condition. Ally gets annoyed that he cannot play sports like his friends or go out to the same places. When he does go out, he becomes self-conscious as they have inside jokes and he feels left out. He worries whether they are having a good time when he is there and whether they really want to be doing something else but can't because of him. He also has to plan ahead whenever he goes out in case there is nowhere to sit to rest, or if there are stairs, which he struggles with due to his PH. The last time he was out he felt exhausted and had to leave after an hour. He felt embarrassed and called himself an ***"idiot"*** *and said that* ***"he should know better"****. Most social interactions now feel exhausting for him. Ally tends to stay alone in his bedroom and has become good at coming up with reasons why he can't go out. He has noticed his social group has reduced over time, which further fuels his negative thoughts about himself.*

Ally has always been close to his parents but recently he has been distancing himself from them and often lying, saying he is fine when really he can't remember the last time he felt fine. Ally has also noticed he and his girlfriend have drifted apart. He used to enjoy physical contact with her but now he feels he does not have the energy. Ally experiences negative thoughts about himself; for example, thinking ***"why would people care about me"*** *or* ***"why do they want to spend time with me; I am boring and a failure"****.*

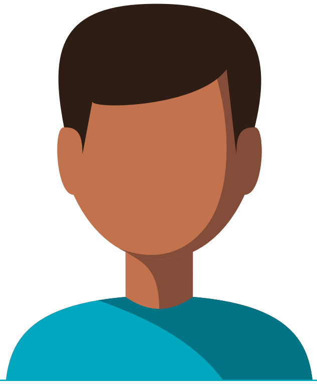

Some people with PH are aware that they are experiencing low mood or depression and may have already discussed this with a healthcare professional or their family and friends. However, many people may not feel able to talk to others about their mental health due to feelings of shame, stigma, or thoughts of hopelessness. For example, people may fear being judged negatively by themselves or by others for having such experiences. Some individuals may also be in denial about how they are feeling, such as pretending it is not a big deal or that it is not having an impact on them, when it really is. Some people may be hoping that their depression will go away on its own; however, we know that if people do not actively manage their depression, it is unlikely to improve.

If depression goes unrecognised and untreated, then it can have a negative effect on your health and wellbeing. So remember, you have taken a positive and brave step today in making the decision to help develop skills to overcome feelings of low mood and depression.

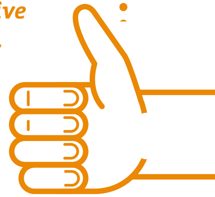

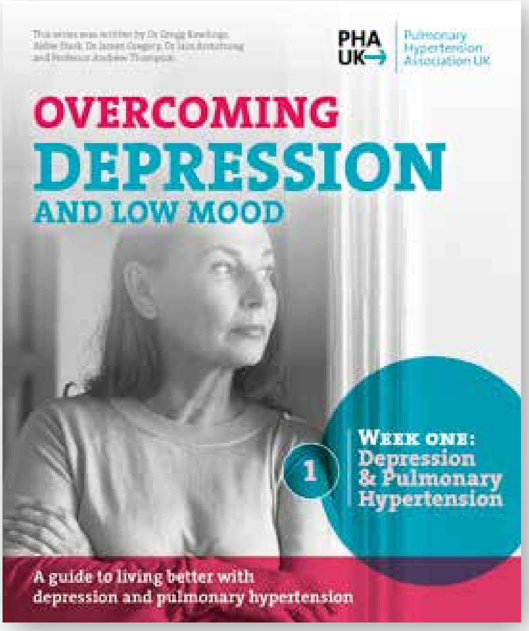
PHA UK
Pulmonary Hypertension Association UK
OVERCOMING
DEPRESSION
AND LOW MOOD
1
Week one:
Depression & Pulmonary Hypertension
A guide to living better with depression and pulmonary hypertension

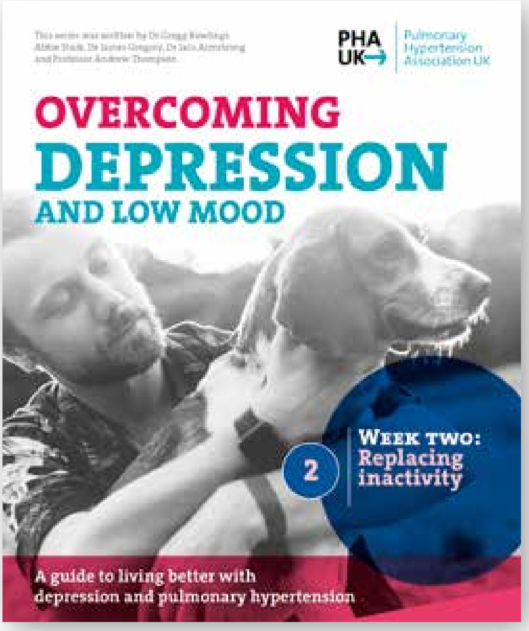
PHA UK
Pulmonary Hypertension Association UK
OVERCOMING
DEPRESSION
AND LOW MOOD
2
Week two:
Replacing inactivity
A guide to living better with depression and pulmonary hypertension

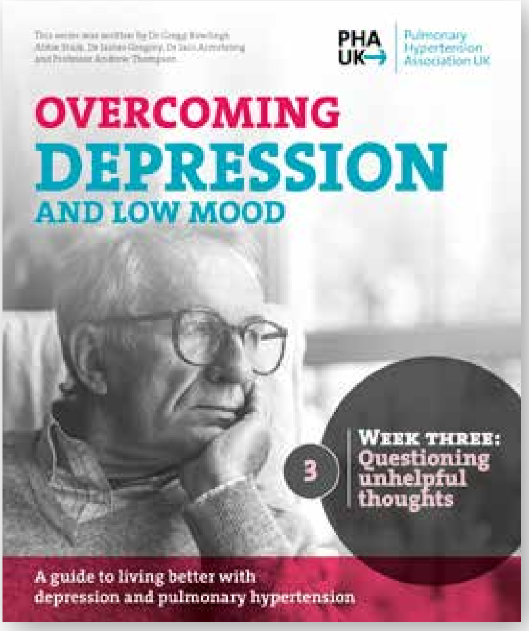
PHA UK
Pulmonary Hypertension Association UK
OVERCOMING
DEPRESSION
AND LOW MOOD
3
Week three:
Questioning unhelpful thoughts
A guide to living better with depression and pulmonary hypertension

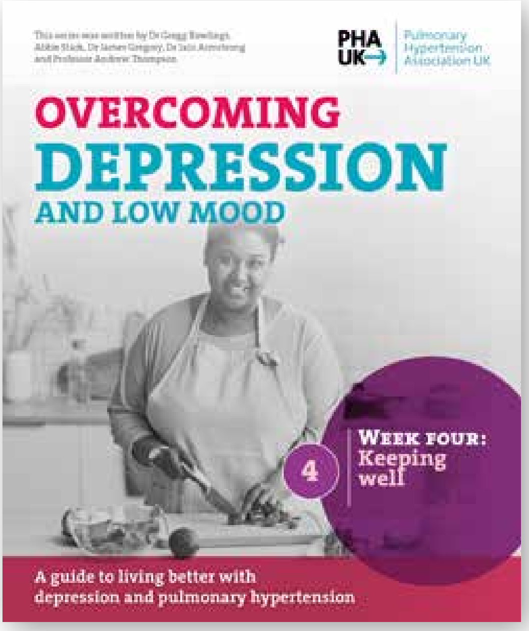
PHA UK
Pulmonary Hypertension Association UK
OVERCOMING
DEPRESSION
AND LOW MOOD
4
Week four:
Keeping well
A guide to living better with depression and pulmonary hypertension

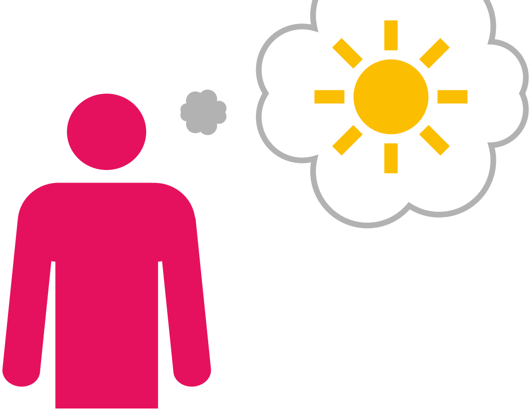

How to use these booklets

The goal of this series of four booklets is to help you to better understand the relationship between depression and PH and empower you in developing more helpful ways of coping. The information is from an evidence-based psychological treatment called Cognitive Behavioural Therapy, or CBT for short.

CBT can help you manage depression by changing the way you think (the cognitive part) and what you do (the behavioural part). A similar intervention focusing on anxiety has shown to help people with PH.

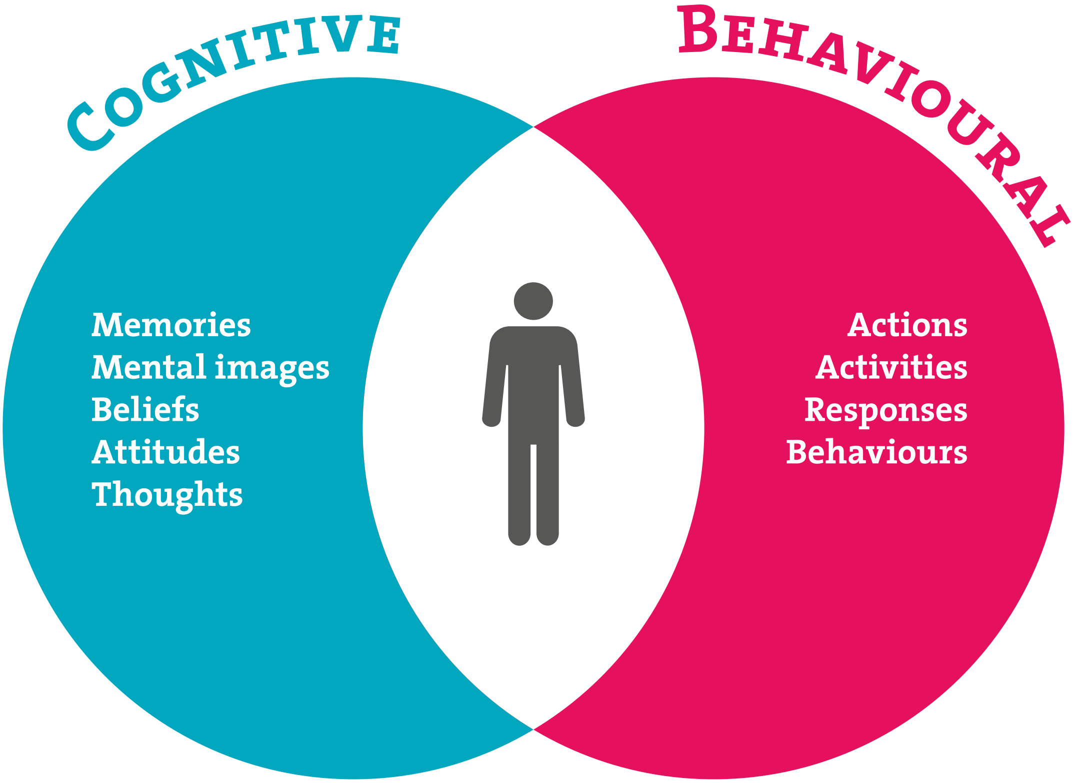

You should read each booklet in order, giving yourself time to understand the different sections before moving onto the next.

Some people read the booklet in one go, while others may read it bit by bit; the best way is what works best for you! Each booklet should take about one week to read and practice the different strategies. This means the four booklets should take four weeks to complete. There is at least one strategy for you to practice over the following week, which aims to help you develop skills to manage low mood.

As you go through each booklet, you will gain a better understanding of depression, how depression can become a problem, and how depression and PH can impact on each other. You will also develop useful strategies aimed at better managing depression in PH.

We would recommend that you tell a family member or friend that you are working through these booklets. Ask them if they would like to also read them along with you and provide support when you are making changes. We know that getting support from other people can help people with PH to manage the impact of low mood and depression.

It will not be enough to just read these booklets. If you want to make a real change in your life, you will need to commit yourself to the tasks and practice. We know that people who practice the strategies are typically the ones that gain the most benefit, so it is important to give these ideas a go. It is also the case that those who make changes from the very start, rather than waiting to make a positive change, are also more likely to find it helpful.

It is important that you keep practicing the exercises.

The more effort you put into each booklet, the more likely your depression will get better. It can be useful to think of mental health like physical health. If someone sets out to walk three miles without stopping, then they may need to train and practice often. Once they are physically fit enough, then they can achieve their goal. However, if they then stopped practicing, their fitness would reduce. Mental health is very similar. The more you practice activities designed to improve your mental health, the better it will be. As soon as you stop doing helpful activities, your mental health may begin to suffer.

We also know that when people have experienced depression, the chance of them experiencing it again is higher. This is another reason why it is important to keep practicing the exercises in this series, as it may help to prevent you from experiencing depression in the future.

If you need further support after reading these booklets, we recommend that you contact your healthcare provider. We have also provided a list of recommended contacts at the end of each booklet, who may also be able to provide you with support.

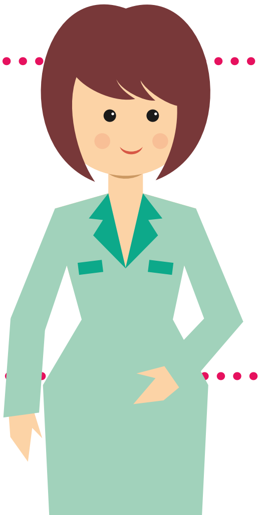

Remember, you are not alone in your difficulties with PH. Here are some real testimonies from other people with PH who have difficulties with low mood.

I was diagnosed with mild anxiety and depression during lockdown, but I think I've been suffering with this for much longer.

On a bad day I feel pretty useless and worthless. I feel frustrated by my conditions but know this will be short lived. I try to write off the day and tell myself: 'Tomorrow is a new day, all will be well'.

I had a telephone consultation with my GP [about it] and I decided I would practice mindfulness, and I have also returned to art which has been my saviour. Added to this I am lucky to have family who are extremely supportive.

PHA UK member

I was diagnosed with PH a few years ago; it never really bothered me. I was given medication and just got on with life. Symptoms were mild, life just went on, but then I suffered horrific anxiety.

Every palpitation, every time I caught my breath... I was over-thinking every symptom and panicking that any second, I would 'pop'. The fear of leaving my children was crippling.

I stayed indoors and always needed my partner with me 'just in case'. I took time off work, I visited A&E multiple times, I tried to distance myself from loved ones.

I tried many medications to try and control my depression and anxiety but had bad reactions to most. I have found podcasts, music and breathing exercises have helped me.

I'm not over it, not by a long shot, but I'm working slowly every day to overcome the fear. I would ask anyone with PH that starts to feel down to seek help immediately as although it's a very misunderstood disease, we are not alone.

PHA UK member

What exactly is depression?

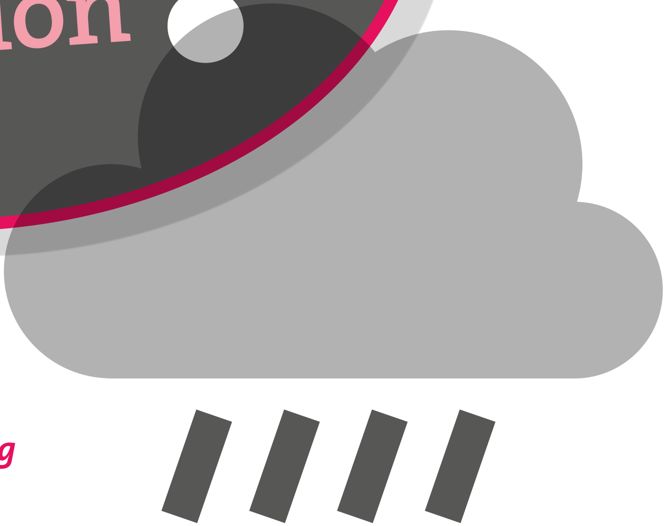

“*Sadness*”, “*low mood*”, “*frustration*”, “*upset*” and “*feeling blue*” are just some of the words that we might use to describe depression. It is normal to feel low mood and upset from time to time; after all, everyone experiences setbacks in their life and encounters hardship and difficult life events.

Given the challenges you face due to a life with PH, you may be more likely to experience difficult events that make you feel sad and upset than people without PH.

Low mood is different from depression, as it can be a healthy and natural response that we need to experience to understand and learn from what we have been through. ‘Clinical’ or ‘major’ depression on the other hand can be more intense, can last much longer (for example, lasting several weeks to months) and it can affect you in your everyday life. In other words, it stops you from living the life that you want.

Depression is often triggered by adverse or difficult life events, and is a more severe form of distress than low mood. It can become a long-term condition in its own right.

✓

Normal experience

✗

Not normal experience

Happy

Low mood

Depressed

Normal response to hardship
Interferes with life
Can still get on with life
May get better over time

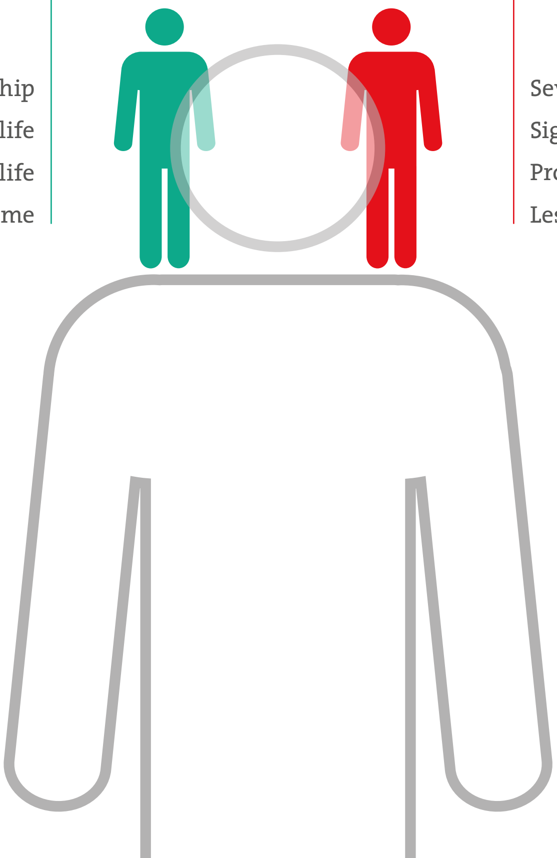

Severe low mood
Significantly interferes with life
Prolonged difficulties
Less likely to improve by itself

DEPRESSION...

...is a subjective term, which means not everyone with it will experience the same difficulties.

Also, while some people can find it hard to understand, a person with depression may not feel low all the time. For example, it is very common for their level of depression to change throughout the day, with people feeling their lowest when they first wake up in the morning and their mood improving throughout the day.

Some people with depression can experience happiness, whereas others just experience sadness. However, this happiness may not last long or it feels different to when they felt happiness in the past. It is important that depression is managed, as in addition to the distressing and challenging symptoms that it causes, which we will look at soon, it can have a series of knock-on consequences.

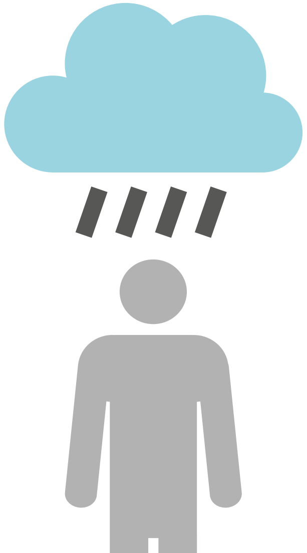

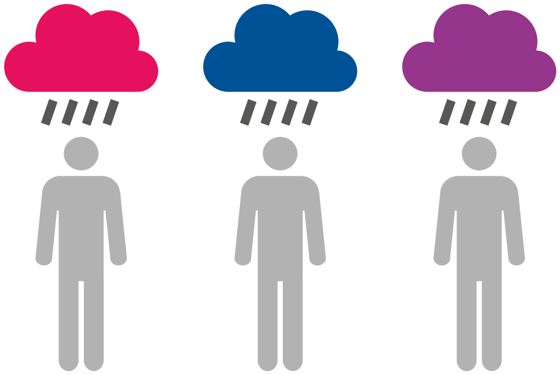

It can:

- *Increase our risk of developing more difficulties, such as anxiety and sleeping problems*
- *Have a negative effect on our relationships and social life*
- *Lead to fatigue and loss of energy*
- *Reduce our physical condition, making it more likely that we are out of breath and find it more difficult to do physical activity*
- *Weaken our immune system and make us more susceptible to illness*
- *Make us experience aches and pain*
- *Stop us from living our lives*
- *Result in us engaging in even more unhelpful ways of coping that can actually maintain our depression*

To find out more about depression, please spend the next five minutes watching the video at the following link. Just type the link below into your web browser.

This video has been watched by over 23 million people!

www.bit.ly/MoreAboutDepression

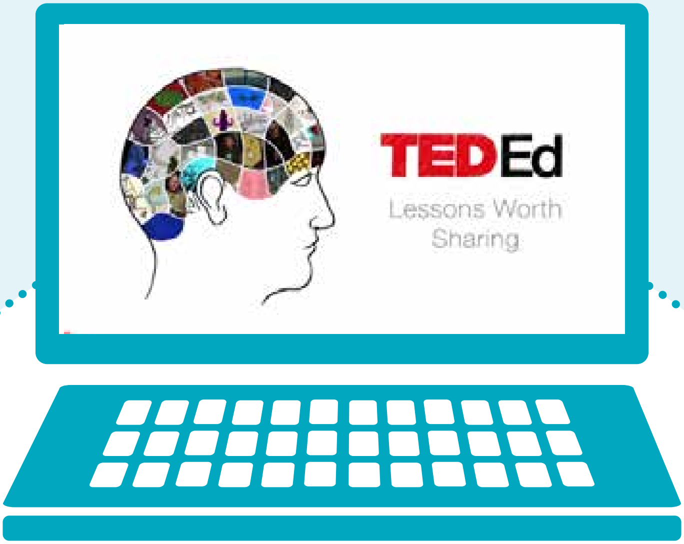

You may want to write down some of your thoughts and reflections from watching the video.

What causes depression?

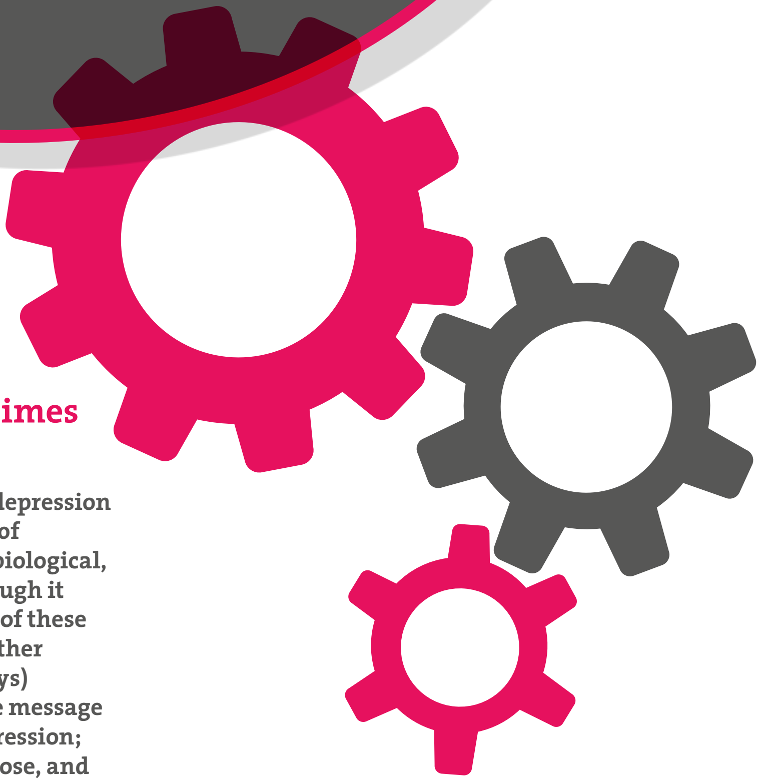

The person experiencing depression is not the cause of it, although it can sometimes feel like this.

It can be helpful to understand that depression is not caused by one thing, but a range of difficulties. We can split them up into biological, psychological, and social factors; although it is important to remember that not one of these things alone can cause depression. Further depression is often (although not always) triggered by life events. The take-home message is that it is not your fault you have depression; you are not doing these things on purpose, and you do not deserve to be experiencing these difficulties.

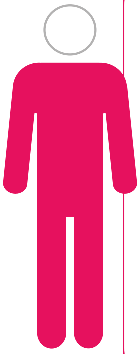

BIOLOGICAL FACTORS

Biological factors associated with depression include a range of ***inflammatory and neurological*** changes that can play a role in maintaining the condition. Some people may have a vulnerability to these factors being activated, and for others, these can be triggered by other disease or illness processes. Nevertheless, whilst some of these are typically out of our control, there are things you can do to help to cope with the symptoms of depression and help to reduce the likelihood of developing it. As you engage with these booklets, you will start to develop some of these skills. In addition, it is also important to recognise that for some people, medication can sometimes also be helpful. Your GP or family doctor will be able to provide you with more information and inform you whether you may benefit from medication to help you with depression.

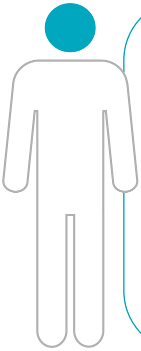

PSYCHOLOGICAL FACTORS

Psychological factors for depression can include ***how you think and how you respond*** to an event. Experiences in your life such as difficult news, challenging events, loss, trauma and anxiety can make us more likely to experience depression. How you view yourself, others and the world may also make you more vulnerable to experiencing depression. For example, if you have a tendency to be critical or negative towards yourself, then you are more likely to feel low. In fact, we know that depression in people with PH is associated with thoughts of self-judgement. We will discuss this more later.

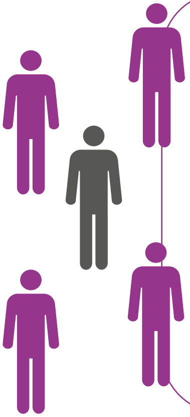

SOCIAL ENVIRONMENT

Our social environment also has a role. We know people who are having difficulties in their ***relationships or that feel isolated or lonely*** are more likely to have difficulties with depression. This is because humans have evolved over millions of years to be social. In other words, our bodies are designed to be in an environment where we feel we have a purpose in life and where we feel connected with other people. It makes sense that isolation is associated with depression in people with PH. Of course, we are all different and some people may like social interactions more than others. However, whether you are an extrovert (someone who enjoys social events) or an introvert (someone who enjoys their own company more than that of others), feeling connected to other people is important.

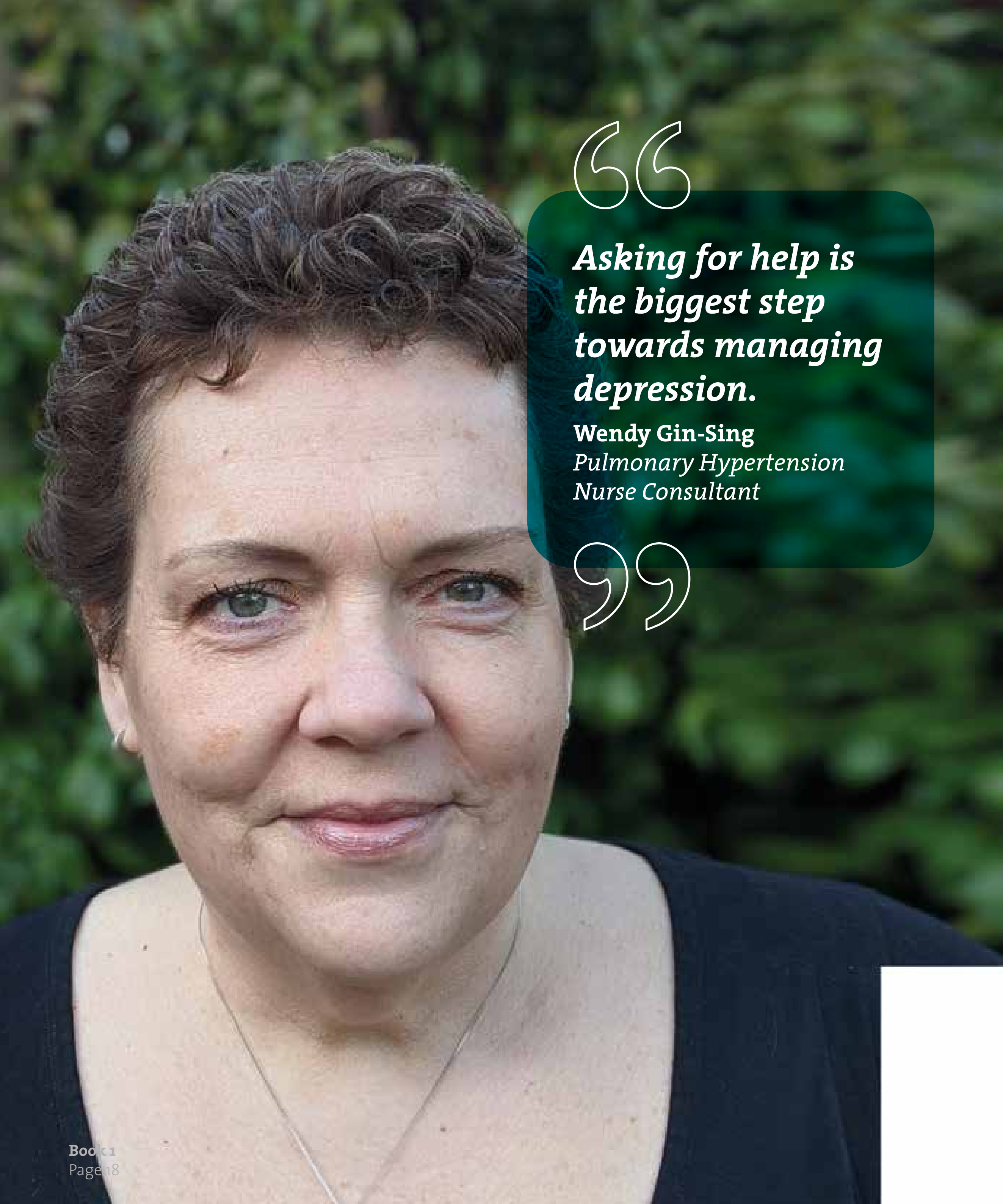

Asking for help is the biggest step towards managing depression.

Wendy Gin-Sing
Pulmonary Hypertension Nurse Consultant

What are the common symptoms of depression?

The first step in learning how to better manage your depression is to recognise the symptoms. As you do this, you will become better at telling apart what symptoms are caused by depression, what are caused by PH, and what could be both.

Depression can be sneaky and negatively colour our thoughts in three main ways. For example, we can see how it has impacted on Ally's thoughts:

1

How we feel about others:

e.g.

"People always let me down"
"I can't trust people"
"People make fun of me"

What we think others think of us:

e.g.

"Everyone thinks I am rubbish"

2

How we think about ourselves:

e.g.

"I'm useless"
I'm pathetic"
"I can't do this"
"I am such a failure"

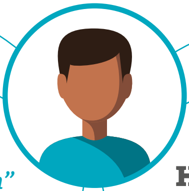

3

How we think about the future:

e.g.

"There's no point"
"There's nothing for me here"

If you could see these thoughts in someone else's head, you wouldn't be surprised that they were feeling low, but it doesn't have to be this way.

Along with negative thoughts, depression is also associated with negative **FEELINGS** and challenging or painful **PHYSICAL REACTIONS**, all of which influence your **BEHAVIOUR**.

On the next page is a list of other common symptoms of depression. Please tick any that you experience regularly.

Remember, feeling low or down can be normal from time to time, and just because you experience one of these symptoms, it does not mean it is bad. You may want to revisit this section as you work through the booklets and become more aware of the symptoms of depression.

HOW YOU THINK

Dwelling on the past

Constant worrying

Struggling to concentrate and poor memory

Seeing the negative in most things

Always thinking the worst will happen

Thinking that you are going crazy

Repetitive negative thoughts, also known as 'ruminating'

Your thoughts or behaviours have slowed down

Negative thoughts about yourself such as thinking you are worthless, lazy, stupid or weak

Negative thoughts about other people or the world, for example: *"There's no point, no one cares, everything always works out badly for me"*

Negative thoughts about the future, for example: *"It always works out terrible", "there's nothing I can do",* or *"it will never be the same"*

Going over and over in your mind what you think will happen in the future

Self-critical thoughts, for example: *"I can't cope", "I'm useless", "I'm a failure"*

Trying to guess what other people are thinking of you and often coming up with a negative evaluation

Distressing thoughts that you cannot stop coming into your mind

Thinking that you could or should do better

You don't think there is much point in doing things you once enjoyed

Thinking that others are better than you

Thinking it is your fault if something bad happens

If something good happens, thinking it is because of someone else or down to luck

Having inflexible thoughts about how you or others should behave

Replaying negative events in your mind

Other(s):

____

____

HOW YOU FEEL

Lack of interest in things you used to enjoy

Lack of enjoyment or joy

Agitated

Quick to anger

Frustration

Blunted emotions

Numb

Low

Sad

Anger

Like you want to cry

Guilty

Self-blame

Shameful

Low motivation

Detached

Pessimistic

Hopeless

Lonely

Lethargic or sluggish

Inadequate

Low confidence

Low self-esteem

Embarrassed

Under pressure

Overwhelmed and like everything feels too much

Other(s):

____

____

____

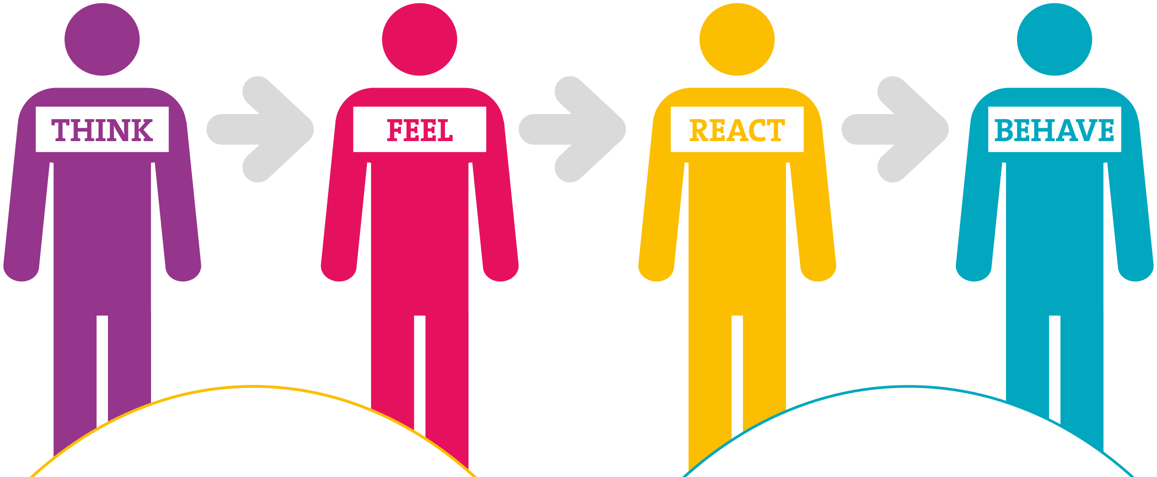

PHYSICAL REACTIONS

Putting on weight
Losing weight
Increased appetite
Lack of appetite
Sleeping more often
Sleeping less often
Struggling to sleep
Pain
Aches
Crying
Lack of energy
Fatigue (this can be mental or physical fatigue)
Struggling to rest or relax
Feeling surges of energy
Other(s):

____

____

YOUR BEHAVIOUR

Finding it hard to switch off
Trouble starting or completing jobs
Waking up in the middle of the night
Decline in sexual interest
Lashing out at others
Finding it difficult to fall asleep
Spending more time in your bed
Eating more or less
Drinking more or less
Smoking more
Drinking alcohol more
Taking recreational drugs
Withdrawing from people
Talking more or less than usual
Avoiding situations, people or events
Seeking reassurance from others
Other(s):

____

____

Many people do not realise there is a link between what you think about a situation, how you feel and what you do. Here is an example of one of Catherine's difficult experiences.

Notice that the arrows have a head on both sides; this means that it can go in both directions. For example, our thoughts can influence our emotions, and our emotions can influence our thoughts.

At first it may be easier to read the circles in order (1, 2, 3 and so on) but in real life, it may feel like they are all triggered by the event.

1 SITUATION

Wakes up in the morning and remembers her grandchildren are coming around at 10am.

2 CATHERINE'S THOUGHTS

I need to do... [creates a list of all the things she feels she has "neglected"], and thinks "what's the point", "who would find me fun to be around anyway", "I just need to pull myself together", ruminating over how PH affects her and what she is missing out on because of it.

3 CATHERINE'S BEHAVIOURS

Stays in bed until 9am and the only reason she gets up is because she needs to take her medication. She isn't feeling up to it, so she rings her son and tells him it's probably best her grandchildren do not come. Lays on the sofa for the rest of the day.

4 CATHERINE'S PHYSICAL REACTIONS

Crying, fatigue, becoming more aware of her aches and pains, lack of appetite.

5 CATHERINE'S FEELINGS

Numb, low, sad, shame and self-blame, anger, lonely and as if she wants to hide from the world .

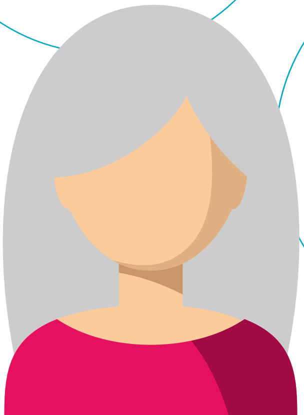

Task

Think of an event that caused you to experience some of the symptoms of depression and fill in the boxes below with your own thoughts, feelings, physical reactions, and behaviour.

Sometimes it can be difficult to identify our thoughts. It may feel as though our heads are very 'full' and that there are no specific thoughts. Sometimes it can help to think; "If my mind was on a TV screen, what would other people see?" this might help you to notice what is on your mind.

Some people find that they have images in their mind rather than thoughts, therefore drawing an image about what is on your mind might also be helpful.

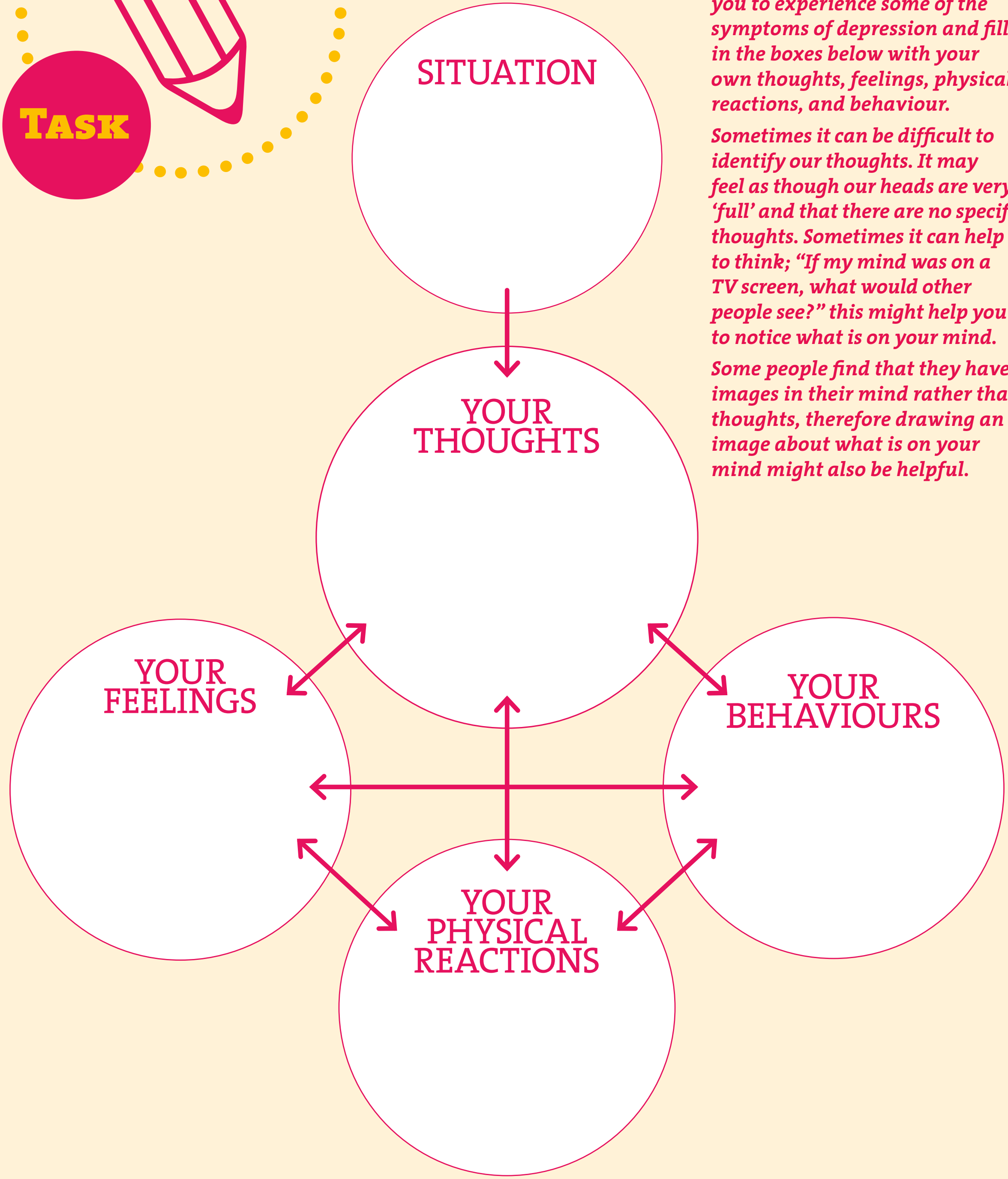

Please spend the next 5 minutes watching the video below. This person describes his experience of depression, referring to it as a big black dog that follows him around. Notice that this video has been watched over 11 million times! Just type the link below into your web browser.

Perhaps you may see some similarities in your experiences of depression.

www.bit.ly/I-had-a-black-dog

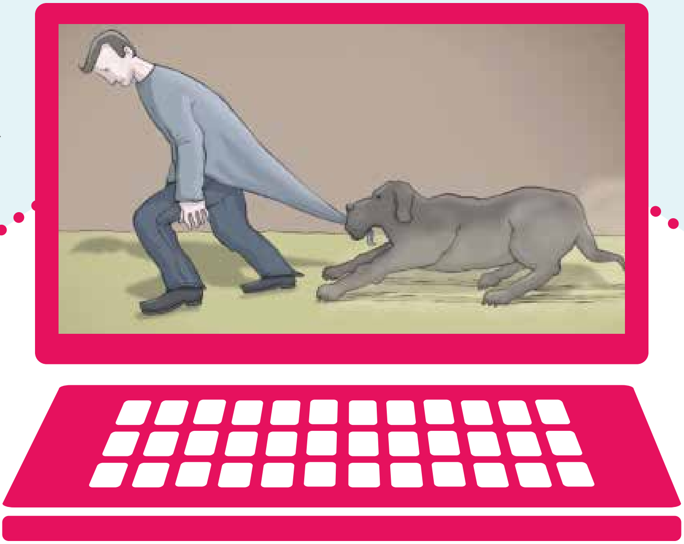

You may want to write down some of your thoughts and reflections from watching the video.

What maintains depression?

As you will have seen in the video, there are a number of ways depression keeps itself hanging around in peoples' lives. You may have started to notice some of them in the diagram you completed.

Over the page are the six most common reasons why depression can be maintained. These behaviours can also prevent people from feeling better. Tick the box if you think it applies to you and please give an example:

1. *Negative thinking patterns*
2. *Repetitive unhelpful thinking styles*
3. *Avoidance*
4. *Unhelpful behaviours*
5. *Low motivation*
6. *Low mood*

1 Negative thinking patterns

People with depression often get stuck in unhelpful thinking patterns; for example, always expecting the worst or calling themselves unkind an self-bullying words. Over time, this will influence how they see and interact with the world. If you think of yourself as 'boring' and 'uninteresting', then you are less likely to speak to others and put yourself in situations where you could make friends. You may even dwell on past conversations with friends, looking for evidence to prove you are 'boring'.

You aren't alone in experiencing negative thoughts. They are really common and can have a negative effect on our confidence, mood and motivation.

Can you identify any of your own negative thinking patterns?

2 Repetitive unhelpful thinking styles

Repetitive negative thinking or 'rumination' is a common symptom of depression. This is where people can go over and over negative events in their head that have happened in the past, or that they expect to happen in the future. This can actually make us more likely to experience events as negative, or remember experiences in a negative way.

For example, Catherine went to the theme park with her family six months ago, but because of her PH, she was unable to go on some rides. While it wasn't a big problem at the time and she enjoyed watching her grandchildren having fun, now when she remembers it, she has a feeling of shame and embarrassment as she felt unable to join in as she would have liked.

What kind of negative things do you repeatedly think over?

3 *Avoidance*

When people experience depression, they often have a loss of motivation or enthusiasm, which gets in the way of them doing things that could actually lift or improve their mood. For example, Ally would enjoy going to the pictures with his friends, but instead, he stays at home watching TV alone. Another example may be that rather than talking to someone about how you are feeling, you put on a brave face and pretend everything is alright. For example, Catherine and her daughter went to a medical appointment for PH and during the consultation, palliative care was mentioned by her doctor. Palliative care is a form of care that aims to improve the quality of life of individuals with life-threatening illness. When they returned home, although her daughter wanted to talk about what the doctor had said and felt it would be helpful for her and her mum to discuss, Catherine explained she did not want to, and needed to go for a lay-down.

Is there anything you have avoided or withdrawn from that you once enjoyed doing, or want to do but are putting off?

4 Unhelpful behaviours

People with depression often engage in behaviours that may help them cope in the short-term but cause them more problems in the long-term. For example, Catherine may feel low as she feels people look at her when she is in the shopping centre because she needs to take regular breaks due to her PH. As a way of coping, Catherine only goes to places where she can walk in one go. Unfortunately, this means the places she can go are very limited and she is finding the list of places is reducing as her fatigue increases. Another example may be using alcohol or smoking as a way to cope. However, in the long-term these behaviours can have a negative impact on our health.

Can you think of any of your behaviours that are unhelpful in the long-term?

5 Low motivation

Loss of energy and interest can result in thoughts such as "It's not worth it", "I will wait until I feel better" and "I am too tired". While fatigue can be common in people with depression and PH, it is important to recognise the differences. When you are tired it can be helpful to rest to recover, but in depression, some rest can actually make you feel worse. It can also give you more time to focus on negative thoughts and ruminate. For example, in the scenario earlier, to avoid talking about what was discussed at her doctor's appointment, Catherine went for a lay-down. But rather than sleeping, she stayed awake going over and over upsetting thoughts in her head.

Can you think of a time when your low motivation has caused you to experience other symptoms of depression?

6 Low mood

As we discussed earlier, feeling low can increase the chances of us thinking negatively and experiencing distressing physical symptoms. One of the most common reasons why people with depression do not engage in more helpful behaviours is because of their low mood. As we will explore next week, it is important to follow a daily plan and structure despite how you feel. For example, Ally wakes up and knows he has a 9am meeting at work. Although he doesn't want to get up, he does anyway and after having a shower and breakfast he notices his mood has improved slightly. However, if he had responded to how he felt when he woke up, he wouldn't have gone to the meeting, probably would have stayed in bed, and would have ended up feeling even lower in mood.

Do you have any examples of when your mood has stopped you from doing something?

We will continue to explore these ***six maintaining factors*** throughout this series.

Summary

In this first booklet, you have learnt how common depression is, what causes it, and how some of the symptoms overlap with PH. Feeling low and sad is a natural and healthy experience, however when this becomes prolonged, severe, or starts to affect your life, it is important to do something about it. Recognising your symptoms and what may be contributing to your depression is the first step.

Focusing your attention on something else, such as a distraction, is one of the best methods to manage unhelpful negative thoughts. The exercises over the next few pages will help you to practice this skill. These activities have helped people with PH who experience difficulties with anxiety.

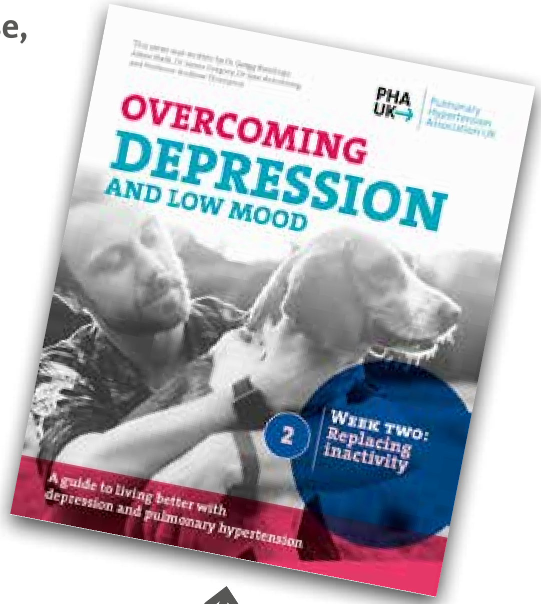

In the next booklet, we will look at how changing your behaviour can help you to manage and reverse some of the effects of depression.

Two Exercises in Mindfulness

for you to practice over the next week

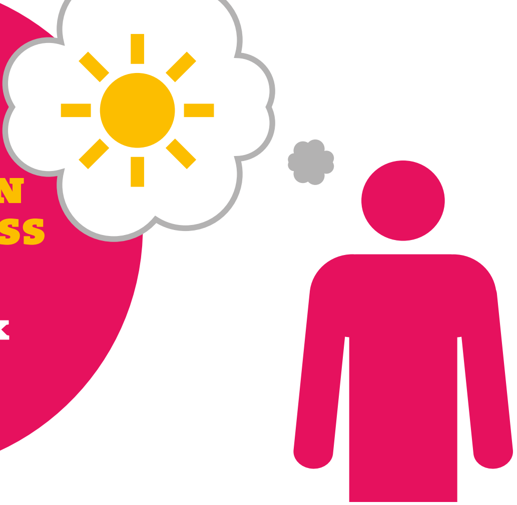

Mindfulness is defined as an awareness that comes from paying attention, on purpose, in the present moment, without judgement.

Mindfulness has been built into Cognitive Behavioural Therapy to guard against relapse in longstanding depression. This is because mindfulness has been shown to be helpful in reducing the likelihood of people experiencing another episode of depression. Due to this evidence, we have introduced a mindfulness exercise for you to practice.

As you have read, where you focus your attention can cause and maintain symptoms of depression. Rather than ruminating about what happened in the past or worrying about future events, mindfulness is an exercise that helps you to be in the present moment.

There are two mindfulness exercises over the next few pages for you to practice over the next week. Please take your time going through the exercises, taking long pauses between each sentence.

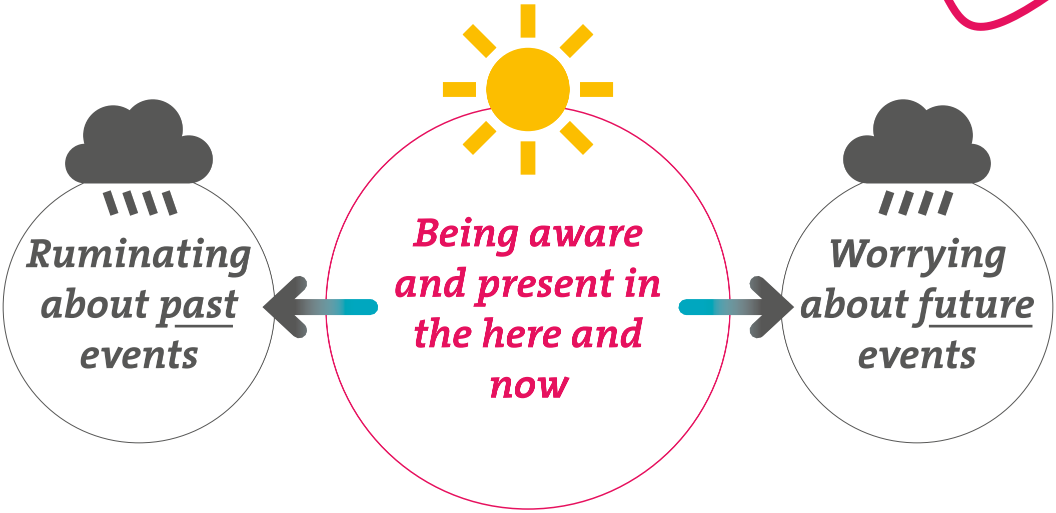

Exercise ONE
Focusing on your body

Get yourself into a comfortable position as you read this. Ideally, sit upright in a chair with your feet flat on the floor, and your back feeling supported by the back of the chair.

Take a few minutes to focus on your breathing. Feel the rhythm of your breathing as you fill and empty your lungs with air, like the waves on the beach coming in before going back out... Focus on your inhale and exhale, noticing the changes in the feelings of your body... Focus your awareness on the physical sensations in your body... Focus on the feelings of pressure where your body comes into contact with the chair and floor... If your mind wanders, just acknowledge your thought without judgment before bringing your attention back to your breathing.

Begin to change your thoughts to allowing yourself to be sitting here focusing on your breathing. There is no particular goal you need to achieve in this moment. Simply just allow the experience to be, without needing it to be anything more than what it is. Do not try to control your breathing, just let your body breathe naturally. If your mind wanders away from your breath to worries, thoughts, concerns or mental images, it is okay. It is normal. When it happens, just notice that your mind has wandered and where it has wandered to. You may want to acknowledge the feeling or sensation you experienced as your mind wandered. Then gently guide your attention back to your breathing. Be kind and compassionate to your mind as it wanders.

When you become aware of any discomfort, tension or other physical sensations, just notice them, acknowledge them, and see if you can make space for them. Do not try to control them or make them go away. See if you can make room for them, just allowing them to be there. Notice the sensations change from each passing moment. Sometimes the sensations may get stronger, while other times, weaker, and sometimes they just stay the same. Just notice the change and allow them to be, just as they are. Breathe calmly into and out from the sensations of discomfort, gently guiding your breath towards where the sensation is in your body.

The goal of this is not to make you feel better, but to get better at feeling.

If you notice you are unable to focus on your breathing, because of an intense physical sensation or intense emotion, let go of the focus on your breath and move your attention to the place of the physical discomfort – the exact place where you experience the feeling the most strongly.

Gently move your attention to the discomfort and stay with it no matter how bad it seems. Acknowledge the sensation... take a look at it.... what does it really feel like? Is it hot or cold? Does it feel sharp or smooth? Does it feel heavy or light? While doing this see if you can make room for the discomfort, allowing it to be there and being willing to stay with it.

*You may also notice thoughts about the sensations. You may notice that you're judging or coming up with ideas about what the sensation means. You may think it is something dangerous. You may start to think about what will happen next. When you notice these thoughts, just acknowledge the thought before bringing your attention back..., not as your mind says it is, but just noticing your thoughts as thoughts, feelings as feelings, sensations as sensations, nothing more, nothing less. If you notice that you're asking yourself questions, just gently answer them with **"I don't know"** and return to your present experience, just as it is.*

*To help you distance yourself from your thoughts and feelings, you may want to start to label them as you begin to notice them. For example, if you notice yourself starting to worry, say to yourself out loud or silently, **"worry, that is a worry"**. Notice the worry without engaging with it, just allowing it to stay by making room for it. If you find yourself judging, just notice that and label it, **"judging, there is judging"**. Observe these thoughts with kindness and compassion. You can do this with other thoughts and feelings, just naming them, such as **"there is planning"**, **"there is remembering"**, **"there is wishing"** or any another label you may notice. Label your emotions and thoughts and move on without judgment. Notice how your thoughts and feelings come and go in your mind and body. You are not what those thoughts and feelings say, no matter how intense or loud they seem.*

Spend the next few minutes practicing this exercise. When you are ready to end the exercise, gradually widen your attention, taking in the noises, smells and sights around you. Allow the awareness of the present moment to stay with you throughout the day.

TURN OVER FOR EXERCISE TWO...

Exercise TWO
Mindfulness in everyday life

This exercise is designed to help you be more aware and appreciate simple and everyday tasks in your life.
Rather than being on autopilot and giving yourself time to worry, the idea is to focus your attention on the activity you are doing. Think of an activity that happens every day more than once – something that you take for granted, like doing the washing up.

*At the very moment you submerge your hands in the hot soapy water, **stop for a moment and be mindful of where you are and how you are feeling in that moment.** Bring your attention to the temperature of the water on your hands. Notice the sensations and smell of the soap and bubbles. Pick up the dirty dishes and gently place them in the water. Watch as the dirt is washed and scrubbed away, leaving a clean smooth surface. Lift the dish out of the water, noticing the water falling off it and splashing back down into the sink. If you notice your mind has wandered, just acknowledge the thought before bringing your attention back to the feeling of water on your hands.*

*When people wash their dishes mindfully, as described above, they reported **an improvement in how nervous they felt,** compared to people who just washed the dishes normally.*

***These mindfulness exercises can be done for a range of everyday activities** such as hoovering, sweeping, eating, showering and doing the laundry. A good activity to do, but very challenging for those of us with a sweet tooth, is to try being mindful while eating a piece of chocolate. Just notice the texture and taste of it on your tongue before taking a bite.*

Try to practice at least one mindfulness exercise each day over the next week.

Mindfulness meditation for BEGINNERS

You may also find it helpful to watch the following video on mindfulness. Just type the link below into your web browser.

www://bit.ly/Flow-Neuroscience

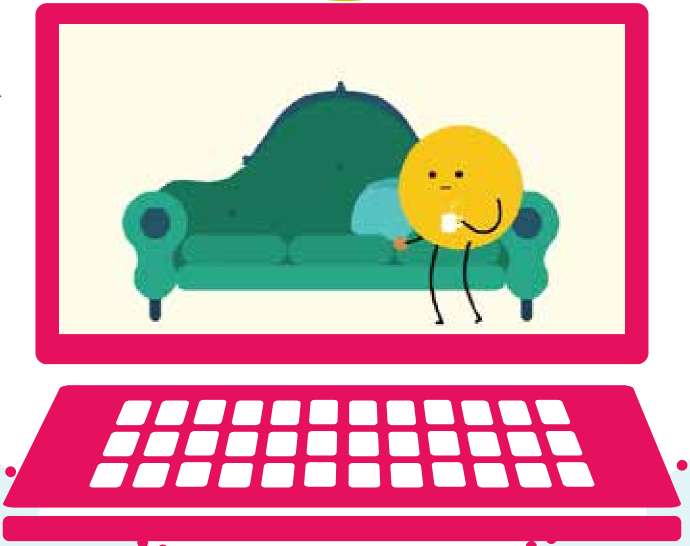

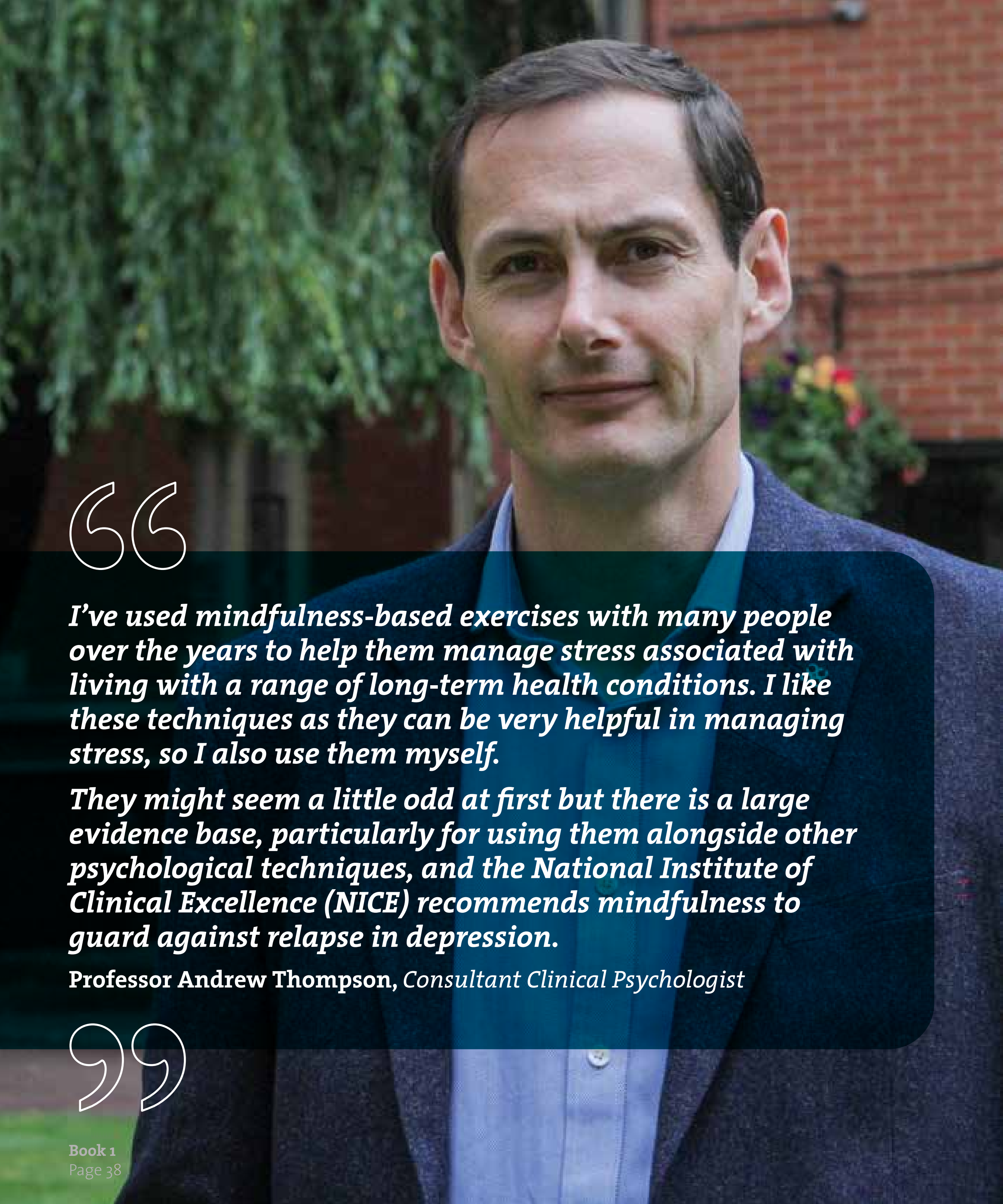

I've used mindfulness-based exercises with many people over the years to help them manage stress associated with living with a range of long-term health conditions. I like these techniques as they can be very helpful in managing stress, so I also use them myself.

They might seem a little odd at first but there is a large evidence base, particularly for using them alongside other psychological techniques, and the National Institute of Clinical Excellence (NICE) recommends mindfulness to guard against relapse in depression.

Professor Andrew Thompson, *Consultant Clinical Psychologist*

Additional resources

If you feel that you need additional information or support, please contact your GP or the PHA UK for advice. We have also included a list of other helpful resources:

Overcoming Worry & Anxiety
(a self-help programme for people with PH)
www.bit.ly/OvercomingWorryAndAnxiety

NHS self-help
www.bit.ly/NHSSelfHelp

PHA UK
www.phauk.org

Depression UK
www.depressionuk.org

Samaritans
www.samaritans.org

Mind
www.mind.org.uk

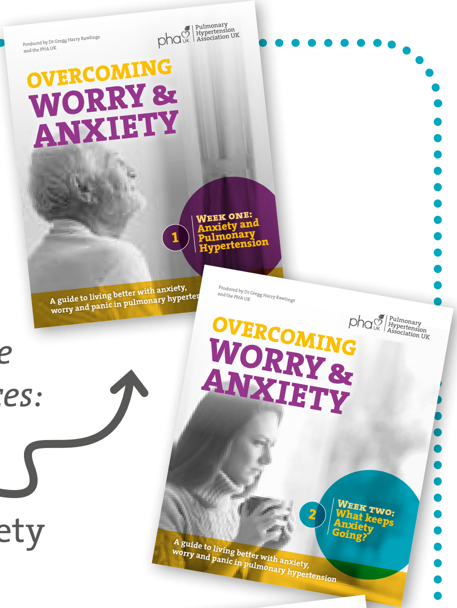

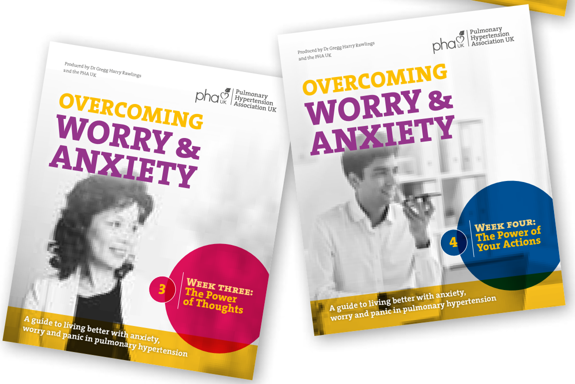

! **Thoughts of self-harm and suicide can also be common signs of depression. If you start to experience any of these symptoms, please speak to a healthcare professional.**

The current study has been designed by researchers working in the UK and it has received ethical approval from an academic institution in the UK (Cardiff University). Participants should be aware and act in accordance with information and governance associated with their country. If you have any questions, please contact your healthcare professional.

For references, see Book 4.

If you're experiencing difficulties with depression or low mood, please tell someone you love and trust.

Paul Sephton,
former Clinical Nurse Specialist in PH

To find out more about the PHA UK, visit **www.phauk.org**

Pulmonary Hypertension Association UK

PHA UK Resource Centre, Unit 1, Newton Business Centre, Newton Chambers Road, Thorncliffe Park, Chapeltown, Sheffield, England S35 2PH

T: 01709 761450 **E:** office@phauk.org @PULHAUK @PHA_UK @PHA ORG UK

Registered Charity No. 1120756. Copyright © 2023 PHA UK. Version 1.2.
