# Supplementary material for: A randomized controlled trial of self‐help cognitive behavioural therapy for depression in adults with pulmonary hypertension
Source: Br J Health Psychol. 2025 Jun 12;30(3):e12800. doi: 10.1111/bjhp.12800 (PMC12159717; doi:10.1111/bjhp.12800)
Supplement: Supplementary file 5 — Data S5. [file BJHP-30-0-s006.pdf]

This series was written by Dr Gregg Rawlings, Abbie Stark, Dr James Gregory, Dr Iain Armstrong and Professor Andrew Thompson

PHA UK

# OVERCOMING DEPRESSION AND LOW MOOD

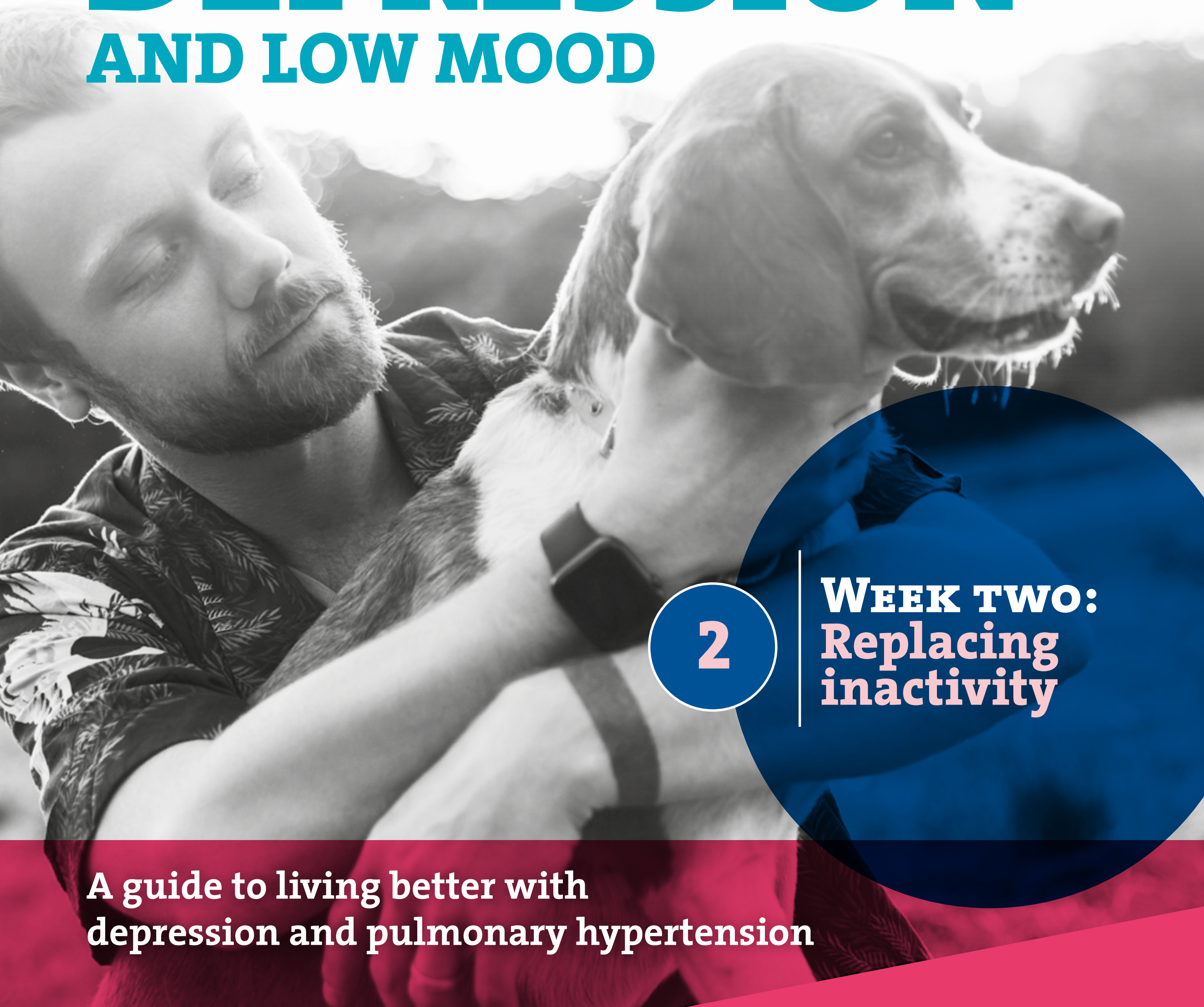

## 2 Week two: Replacing inactivity

A guide to living better with depression and pulmonary hypertension

PHA UK Pulmonary Hypertension Association UK

**Week two: Replacing inactivity**

# Overcoming depression and low mood

**Here is a summary of what will be covered in this booklet:**

- *How changing your behaviour can help you to manage and reverse some of the effects of low mood and depression*
- *The importance of doing meaningful activities*
- *Activity scheduling, which involves planning activities that give you a sense of achievement and pleasure*
- *Tips on how to increase activity levels in a manageable way, especially considering symptoms of PH and how this might physically affect people*

# Replacing inactivity

**In this booklet, we will be looking at how changing your behaviour can help you to manage and reverse some of the effects of depression. As the figure overleaf shows, increasing what you do can help with the symptoms of depression and break the cycles which can maintain depression.**

**For example, as we discussed in the previous book, depression can make people think *"I don't feel like doing anything"*, so they wait for the *feeling* to start the *doing*, and this can get people stuck in a cycle of depression. Sometimes you need to do the doing to get the *feeling* back online and to break the cycle.**

Replacing inactivity and repetitive negative thinking with meaningful and valued activities is one of the most effective ways of treating depression using CBT. This is also known as activity scheduling. Activity scheduling helps people to increase the number of daily activities. Before we show you one way to help you increase your level of activity, it is important we discuss how this can be done while managing the symptoms of PH.

**Compare the two cycles over the page**

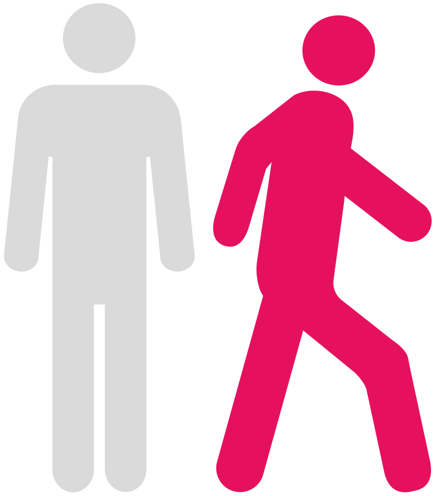

# Inactivity contributing to an unhelpful cycle ***causing*** depression

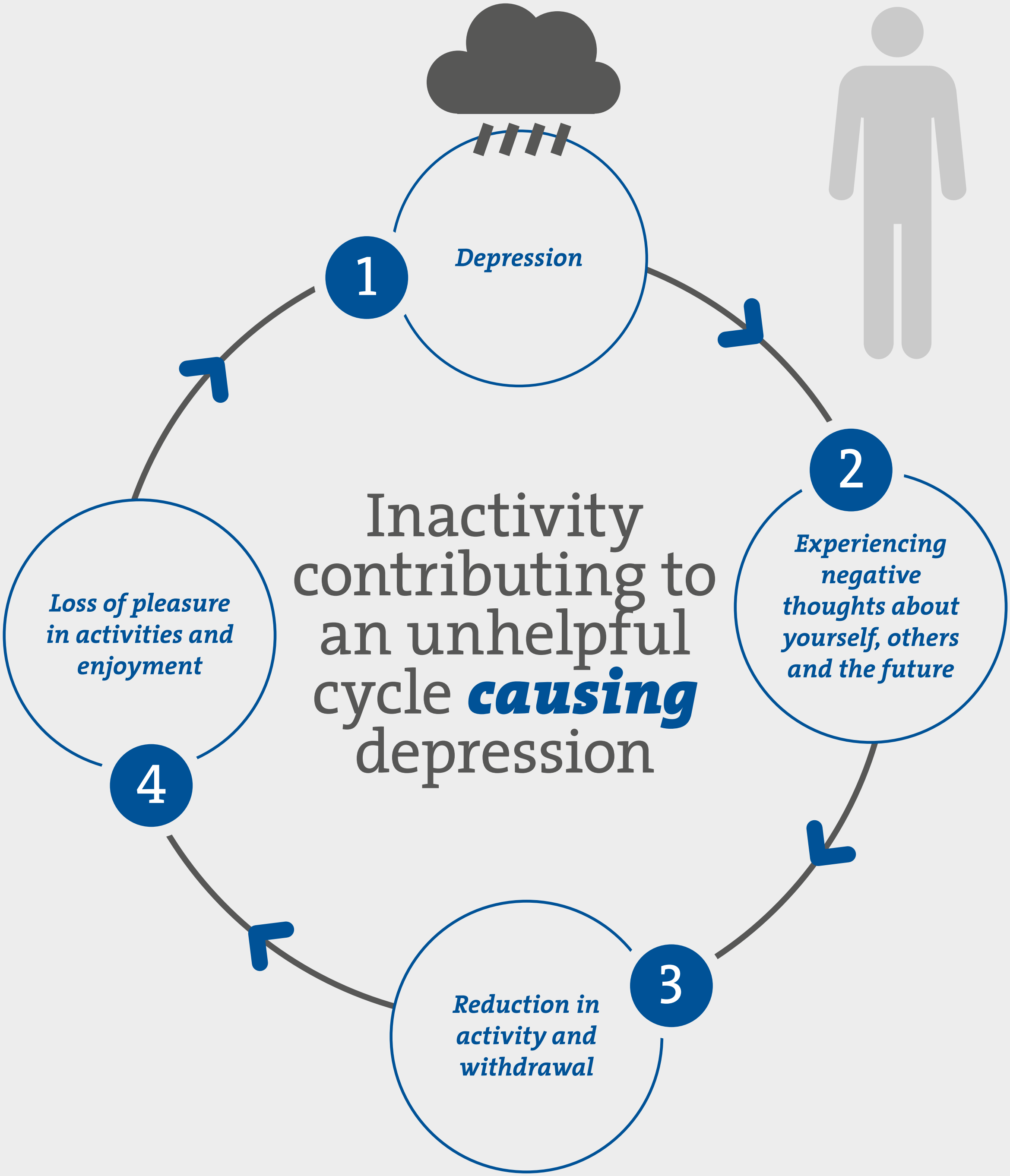

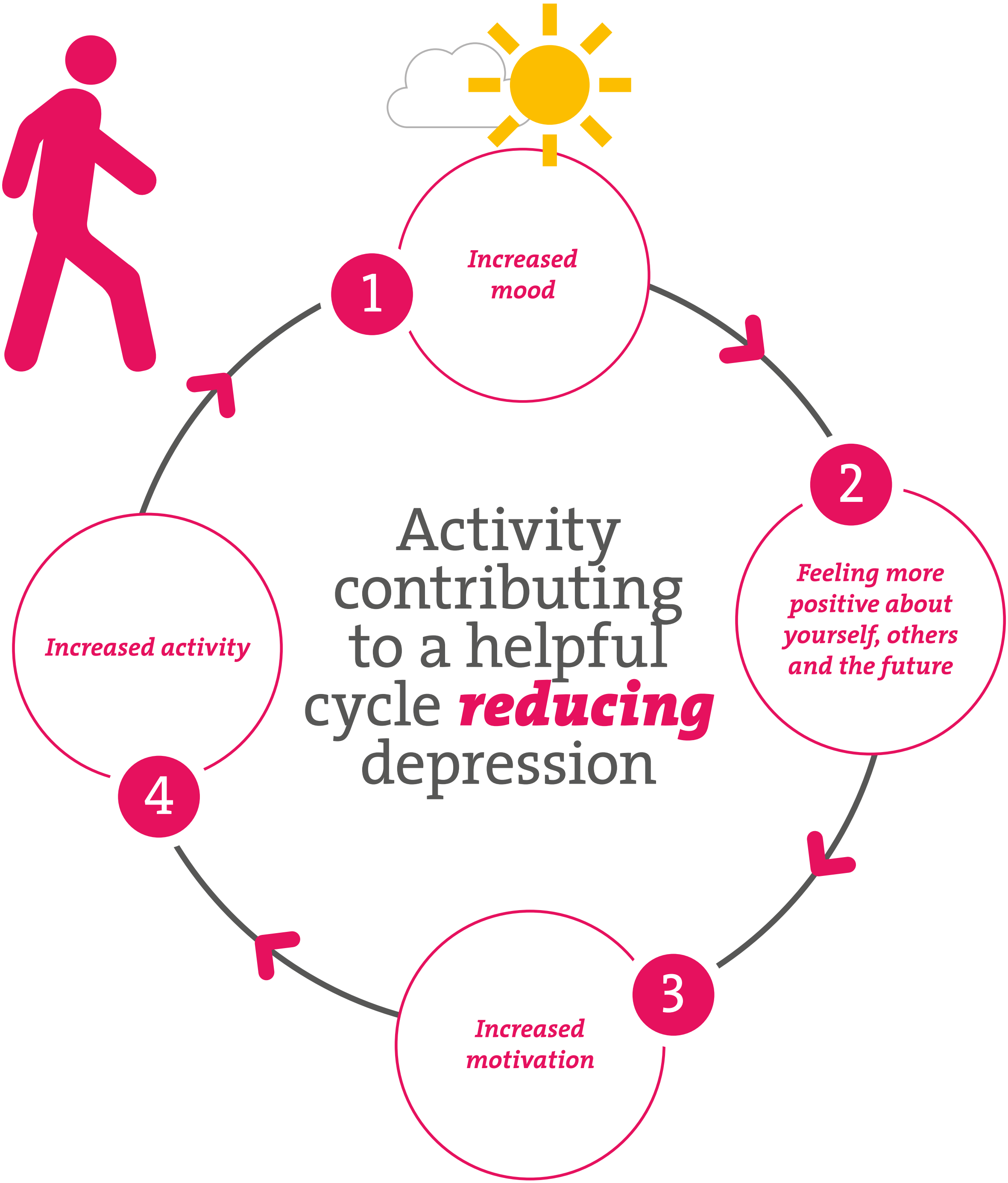
Activity contributing to a helpful cycle reducing depression
1
Increased mood
2
Feeling more positive about yourself, others and the future
3
Increased motivation
4
Increased activity

***"Initial data that we collected during a clinical trial helping people with PH to manage their anxiety seems to suggest changing unhelpful thoughts and behaviours through Cognitive Behavioural Therapy (CBT) helps to reduce symptoms of depression and anxiety. In fact, several of the strategies in that trial are the same ones that are used in this series of booklets. Many people who took part in the trial found CBT helpful and would recommend it to someone living with PH.***

**Dr Gregg H. Rawlings**
**Nottingham Trent University**
Developer of *A guide to living better with depression and pulmonary hypertension* and *A guide to living better with anxiety, worry and panic in pulmonary hypertension*

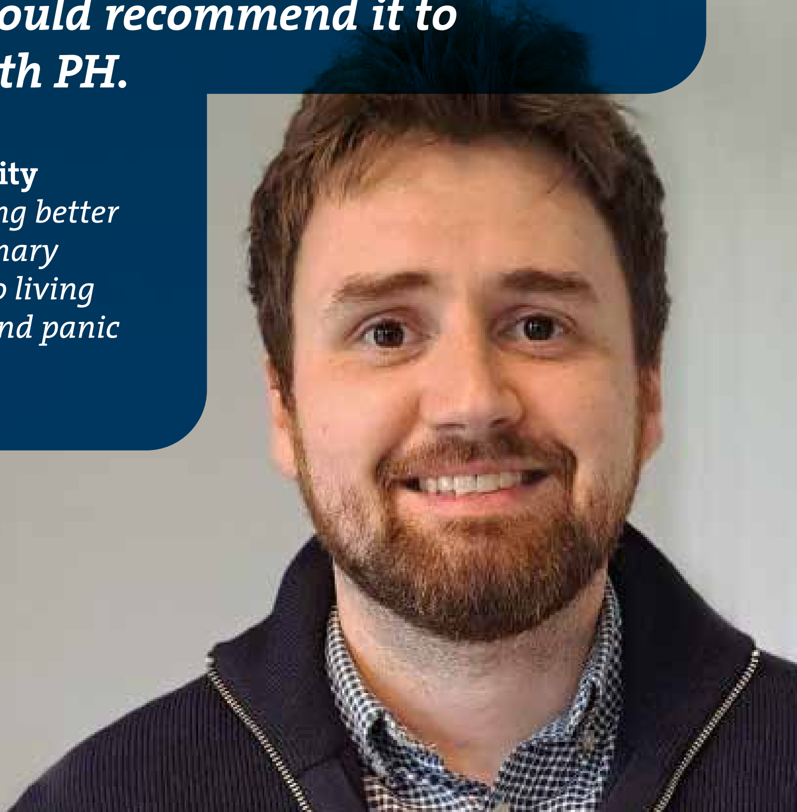

# *Know your* ***limits***

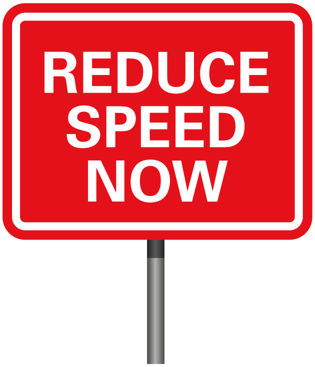


***PH and depression can affect how you do many activities. Behaviours that you used to do before may now make you feel out of breath, pain and tired. If this is the case for you, you are not alone as fatigue is common in people with PH, and people with depression.***

It is very easy to fall into unhelpful patterns of behaviour with fatigue. For example, people with PH may believe the more activity they do, the more likely they are to become fatigued. To cope with this, they do less and rest more.

However, as you read last week, avoiding something because it is distressing is helpful in the short-term, but not in the long-term as it can maintain depression. As the figure below shows, resting too much will mean that you experience less fatigue in the short-term, but in the long-term this can lead to low mood, boredom, repetitive negative thinking and even anxiety.

Also, as you are less active you are likely to be less fit, which means over time it will take less activity to make you feel tired and for you to experience low mood.

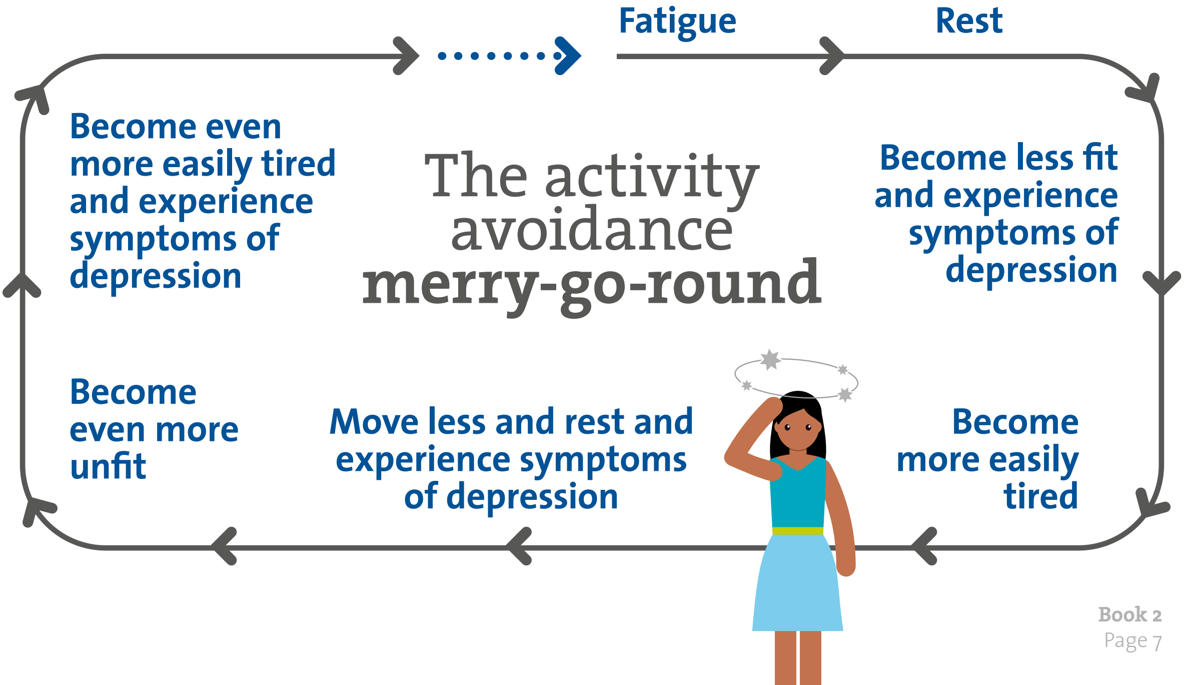

***Another unhelpful cycle is called "boom and bust", where on days people feel they have more energy, they push themselves into doing more work to make up for what they were unable to do on days when they had little energy or greater symptoms of PH.***

It makes sense why people do this, however people then find they need to spend the next few days recovering as a result of over-exertion (or pushing themselves too hard) until they feel like they have more energy and any PH-related symptoms have reduced. Then they start the cycle all over again. What can also happen is as the individual becomes less fit and more easily tired, they can achieve less over time.

Pacing is a coping method that with practice and time, allows you to do activities in a way that helps you manage the difficulties of fatigue. Pacing helps you find a balance between ***doing too much and doing too little***.

Pacing means that you spend just enough time on an activity without pushing yourself to the point where you end up being tired. This approach allows you to engage in more activities and do more over time. The idea of pacing is that your level of activity is increased in small, planned stages at your own pace.

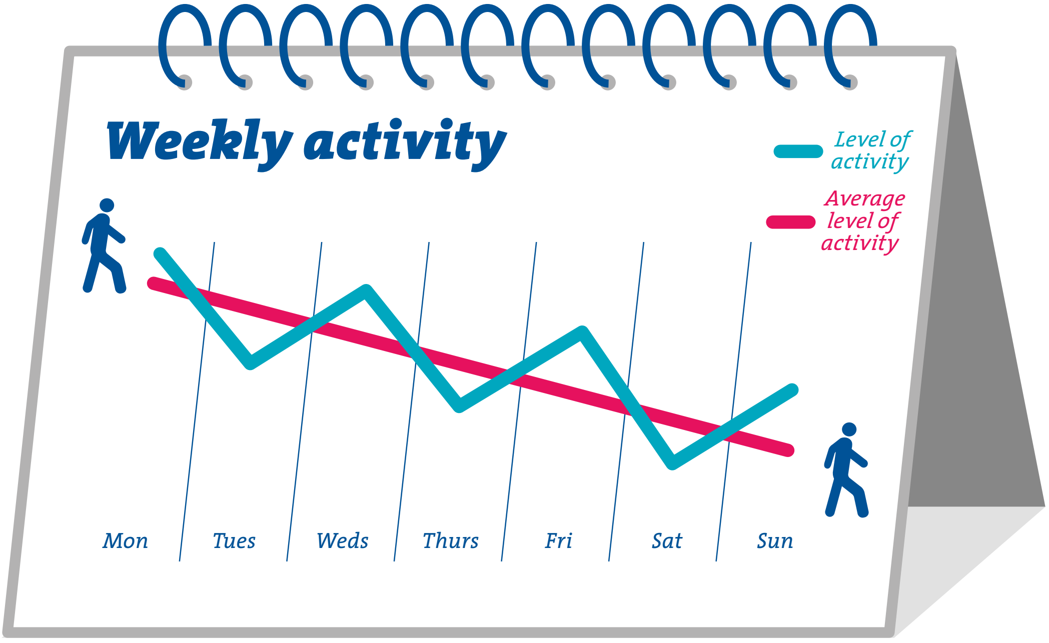

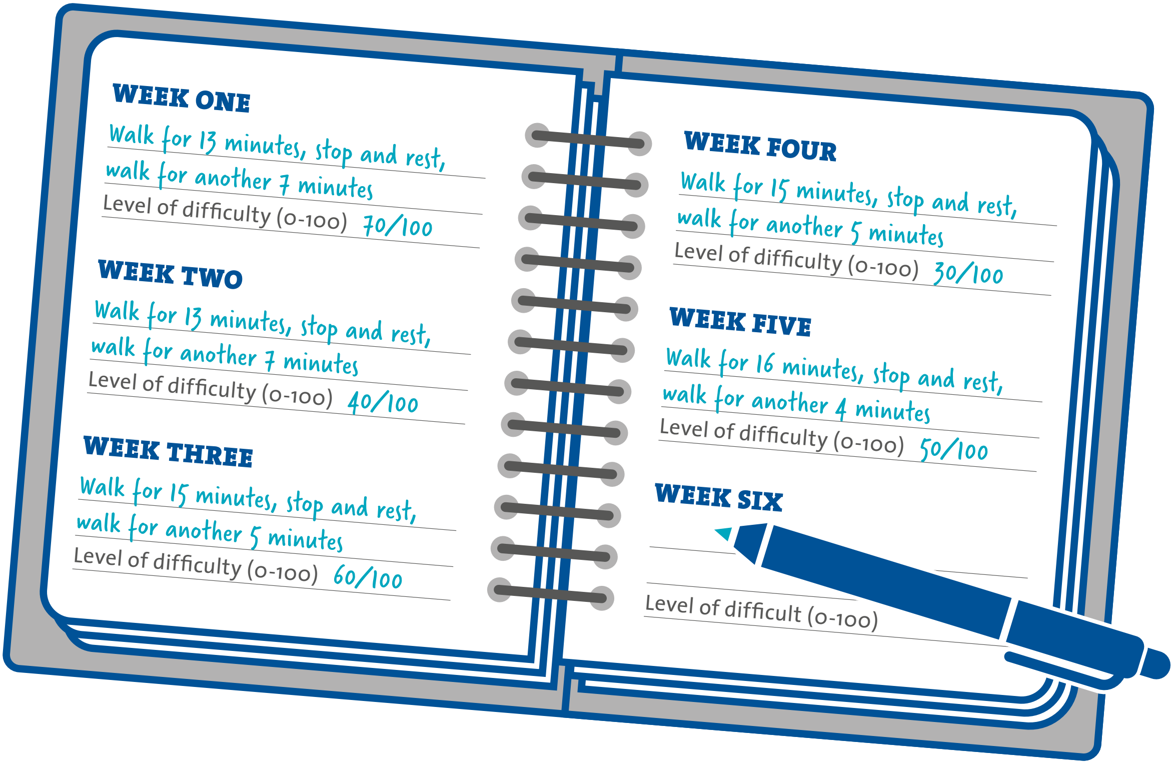


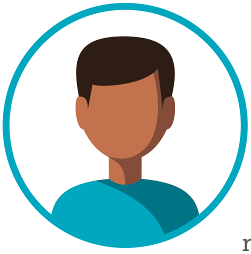

For example, Ally's goal was to walk from where his car is parked to his seat inside the football stadium, which would take him about 20 minutes. Ally measured how long he could walk without stopping on three separate occasions before he felt himself needing to stop. He could manage between 12 and 14 minutes, so Ally's starting point was 13 minutes. Ally started to walk to his chair for 13 minutes and then he stopped to rest. He rated the difficulty of this out of 100. At first, 13 minutes was 70/100 in difficulty but as he repeated this, he found 13 minutes less difficult. He increased it to 15 minutes and repeated it and so on.

Ally noticed that he was getting more confident in his ability and did not feel as anxious or depressed at the thought of not being able to walk to his seat in one go. He practiced his mindfulness (the activity you practiced in the previous book) as he focused on the noises, the smell of food and sights he could see, rather than thinking people were staring at him or whether he was feeling tired or breathing too quickly. He also used it to distract his attention from calling himself unhelpful names such as "useless", "pathetic" and "odd".

Ally started to enjoy his breaks as he would buy a can of pop and listen to other people talking about the football match, which he wouldn't have heard if he stayed at home. Although Ally felt at times he could walk longer, he didn't and kept to his plan.

***Ally managed to achieve his goal of being able to walk to his seat in one go. Ally was pleased with his achievement and used pacing to help achieve other goals.***

# *When using pacing it is important to remember:*

***Figure out your starting point.*** *For Ally it was 13 minutes. The duration of time should challenge you, but not too much.*

***Make a clear goal and stick to it.*** *For example, walking to the post office, hoovering, walking upstairs, sitting in the car on long journeys, only cleaning one room at a time.*

***Use time and not pain or fatigue*** *when deciding when to stop and rest. "No pain no gain" does not apply here.*

***Choose activities that you enjoy and get a sense of achievement from.***

***Taking a break is not a sign of weakness or failure.*** *It is important to let your body recover and help you build stamina over time.*

***Perhaps most importantly, give yourself the credit of achieving something even if you think it is a little gain!***

***Finally, even if Ally did not reach his goal of walking to his seat in one go, it does not mean he failed.*** *I mean, would you think he is failure? He was able to increase his physical fitness, had new and positive experiences, and taken positive steps that will have helped him to manage his depression.*

***Don't let your mood determine your behaviour.*** *Do something despite how you're feeling. It will help improve your mood if you're feeling low and blow off steam if you're feeling frustrated.*

***Don't worry about set-backs, we all experience them.***

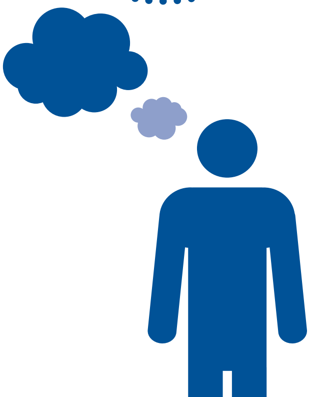

*I've had CBT [Cognitive Behavioural Therapy] and I think it did help. It taught me to rest when needed and don't overdo things on good days. And also, to try to get out of the house more (which I'm still struggling with).*

**PHA UK member**

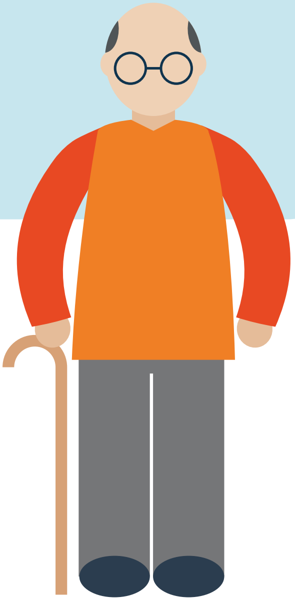

# *Activity scheduling and* ***activating yourself***

**Increasing your level of activity to help manage depression can be achieved in three steps:**

## STEP 3

It is important to recognise the types of activities we do (and don't do). ***Activities can be rated on how pleasurable they are, and how much they give a person a sense of achievement.*** We can use the figure opposite to help us.

## STEP 2

***From day four onwards, start to fill up the rest of your week with a range of mental and physical activities.*** You may have some ideas about what you want to do, however we know this may be difficult for some people or at least at first, so we have provided a list of possible activities. Some activities may be things you do for you, you do for others or you do with others. It is a good idea to look back on the first three days at the amount, type and duration of your activities. Use this as a guide to increase and change what you are doing.

## STEP 1

Look at the daily activity tables on pages **15-23**, which show the hours of the day and days of the week. There is a slot for every waking hour. ***Over the next three days, use the table to record and monitor your daily activities.***

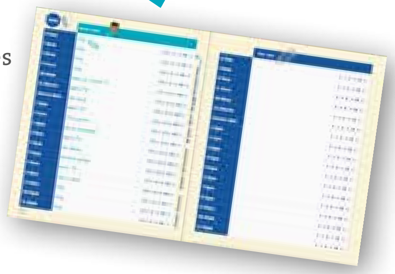

You may want to write things such as, ***"Done the washing", "Went shopping"*** and ***"Spoke to a friend"***. You may not have done anything during that hour and so you could leave it blank, write the word ***"nothing"*** or black it out.

# Tracking your ***pleasure, achievement & mood...***

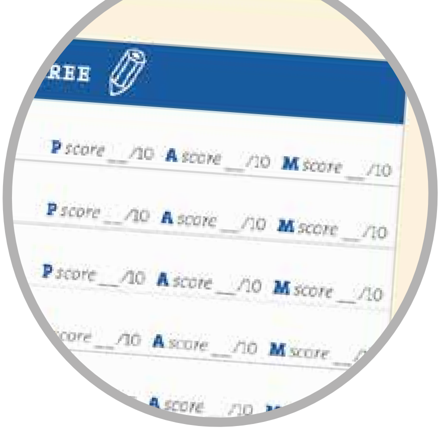


***You will see there are three spaces within each hour in the table to add a score out of 10. This is for you to record the level of pleasure, achievement and mood you experience from each activity. See the scales below.***

***It will be helpful to review your schedule from time to time to see what type of activities you are doing (and not doing).*** It is helpful to find a balance between the two, for example if you are just doing chores, make sure to add some pleasurable activities. If you are spending large amounts of time resting because of your PH, make sure to break it up and engage in mentally stimulating activities.

Finally, it is important to track any changes in your mood as a result of engaging in an activity. This will be useful to see what kind of activities help to improve your mood but also to challenge unhelpful thoughts such as, ***"I am always depressed"*** or ***"I don't enjoy anything"***.

## ***Pleasure*** rating scale

0 1 2 3 4 5 6 7 8 9 10

*No enjoyment at all* — *Average enjoyment* — *Very enjoyable*

## ***Achievement*** rating scale

0 1 2 3 4 5 6 7 8 9 10

*No sense of achievement* — *Average sense of achievement* — *Great sense of achievement*

## ***Mood*** rating scale

0 1 2 3 4 5 6 7 8 9 10

*Low mood* — *Not happy, not sad* — *Happy*

*We cannot just do activities that are pleasurable as we would get* ***nothing productive done*** *such as chores around the house. Plus, we would miss out on the positive emotions associated with achievements, such as a feeling of mastery and confidence. Likewise, we cannot just do activities that give us a sense of achievement because we* ***need to have pleasurable experiences*** *in our lives, whether they are one-off treats or regular events we can enjoy.*

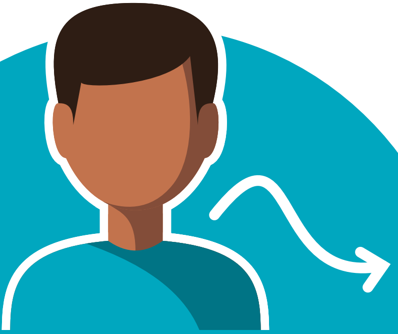

**Here's a typical day for Ally as an example of how to complete the diary, mentioned on page 12.**

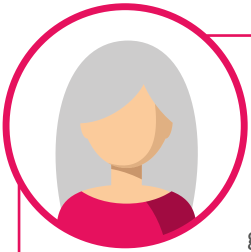

For example, Catherine finds hoovering boring, a chore, and gets ***no pleasure*** at all from it so she would give it a **0 out of 10**. However, she would rate hoovering as an **8 out of 10** on ***achievement*** as she thinks her house looks better for it, and she enjoys watching her grandchildren play with their toys on the floor when they come around to visit her and she knows it's clean.

Remember, it is normal to enjoy experiences and you should not have to feel shame or guilt as a result. If you do start to experience any of those thoughts such as ***"I shouldn't be enjoying myself"*** or ***"what's the point in having fun"***, look back at booklet one and think about the knock-on effect these thoughts could be having on your emotions, physical sensation, and behaviours. It is also important to remember that you may not feel pleasure at first when doing an activity, or you may not feel the same amount of pleasure doing something that you used to enjoy. As your mood starts to lift, those positive feelings should return.

**Remember, for the first three days you are just recording your daily activities. It is on day four when you start to make changes by adding activities that give you a feeling of pleasure or achievement.**

*Before you start to fill in your schedule, make sure to read the rest of the booklet for more helpful advice.*

## Ally's day

| Time | Activity | P | A | M |
|---|---|---|---|---|
| 6-7am | Sleep | P 7/10 | A -/10 | M -/10 |
| 7-8am | Sleep | P 7/10 | A -/10 | M -/10 |
| 8-9am | Sleep | P 7/10 | A -/10 | M -/10 |
| 9-10am | Sleep | P 7/10 | A -/10 | M -/10 |
| 10-11am | Wake up & watch TV | P 8/10 | A 0/10 | M 5/10 |
| 11-12noon | Watch TV | P 8/10 | A 0/10 | M 5/10 |
| 12noon-1pm | Eat lunch | P 8/10 | A 6/10 | M 8/10 |
| 1-2pm | Watch TV | P 8/10 | A 0/10 | M 5/10 |
| 2-3pm | Text partner | P 9/10 | A 3/10 | M 9/10 |
| 3-4pm | Hoover downstairs | P 3/10 | A 9/10 | M 5/10 |
| 4-5pm | Watch TV | P 8/10 | A 0/10 | M 5/10 |
| 5-6pm | Eat dinner | P 7/10 | A 7/10 | M 7/10 |
| 6-7pm | Speak to partner | P 9/10 | A 3/10 | M 9/10 |
| 7-8pm | Watch TV | P 8/10 | A 0/10 | M 5/10 |
| 8-9pm | Have a snack | P 9/10 | A 2/10 | M 7/10 |
| 9-10pm | Sleep | P 7/10 | A -/10 | M -/10 |
| 10-11pm | Sleep | P 7/10 | A -/10 | M -/10 |
| 11-12pm | Sleep | P 7/10 | A -/10 | M -/10 |

*P score = Pleasure A score = Achievement M score = Mood* *See page X to learn more*

## Task

| | Day one |
|---|---|
| 6-7am | **P** /10 **A** /10 **M** /10 |
| 7-8am | **P** /10 **A** /10 **M** /10 |
| 8-9am | **P** /10 **A** /10 **M** /10 |
| 9-10am | **P** /10 **A** /10 **M** /10 |
| 10-11am | **P** /10 **A** /10 **M** /10 |
| 11-12noon | **P** /10 **A** /10 **M** /10 |
| 12noon-1pm | **P** /10 **A** /10 **M** /10 |
| 1-2pm | **P** /10 **A** /10 **M** /10 |
| 2-3pm | **P** /10 **A** /10 **M** /10 |
| 3-4pm | **P** /10 **A** /10 **M** /10 |
| 4-5pm | **P** /10 **A** /10 **M** /10 |
| 5-6pm | **P** /10 **A** /10 **M** /10 |
| 6-7pm | **P** /10 **A** /10 **M** /10 |
| 7-8pm | **P** /10 **A** /10 **M** /10 |
| 8-9pm | **P** /10 **A** /10 **M** /10 |
| 9-10pm | **P** /10 **A** /10 **M** /10 |
| 10-11pm | **P** /10 **A** /10 **M** /10 |
| 11-12pm | **P** /10 **A** /10 **M** /10 |

| Task | Day two |
| --- | --- |
| 6-7am | **P** /10 **A** /10 **M** /10 |
| 7-8am | **P** /10 **A** /10 **M** /10 |
| 8-9am | **P** /10 **A** /10 **M** /10 |
| 9-10am | **P** /10 **A** /10 **M** /10 |
| 10-11am | **P** /10 **A** /10 **M** /10 |
| 11-12noon | **P** /10 **A** /10 **M** /10 |
| 12noon-1pm | **P** /10 **A** /10 **M** /10 |
| 1-2pm | **P** /10 **A** /10 **M** /10 |
| 2-3pm | **P** /10 **A** /10 **M** /10 |
| 3-4pm | **P** /10 **A** /10 **M** /10 |
| 4-5pm | **P** /10 **A** /10 **M** /10 |
| 5-6pm | **P** /10 **A** /10 **M** /10 |
| 6-7pm | **P** /10 **A** /10 **M** /10 |
| 7-8pm | **P** /10 **A** /10 **M** /10 |
| 8-9pm | **P** /10 **A** /10 **M** /10 |
| 9-10pm | **P** /10 **A** /10 **M** /10 |
| 10-11pm | **P** /10 **A** /10 **M** /10 |
| 11-12pm | **P** /10 **A** /10 **M** /10 |

## Task

| Task | Day three |
|---|---|
| 6-7am | **P** /10 **A** /10 **M** /10 |
| 7-8am | **P** /10 **A** /10 **M** /10 |
| 8-9am | **P** /10 **A** /10 **M** /10 |
| 9-10am | **P** /10 **A** /10 **M** /10 |
| 10-11am | **P** /10 **A** /10 **M** /10 |
| 11-12noon | **P** /10 **A** /10 **M** /10 |
| 12noon-1pm | **P** /10 **A** /10 **M** /10 |
| 1-2pm | **P** /10 **A** /10 **M** /10 |
| 2-3pm | **P** /10 **A** /10 **M** /10 |
| 3-4pm | **P** /10 **A** /10 **M** /10 |
| 4-5pm | **P** /10 **A** /10 **M** /10 |
| 5-6pm | **P** /10 **A** /10 **M** /10 |
| 6-7pm | **P** /10 **A** /10 **M** /10 |
| 7-8pm | **P** /10 **A** /10 **M** /10 |
| 8-9pm | **P** /10 **A** /10 **M** /10 |
| 9-10pm | **P** /10 **A** /10 **M** /10 |
| 10-11pm | **P** /10 **A** /10 **M** /10 |
| 11-12pm | **P** /10 **A** /10 **M** /10 |

# "Mind the **Gap**"

**We know that being asked to complete a weekly or even a daily planner may seem like a daunting task at first. You may have even experienced some negative thoughts about it already such as, *"I will never be able to do that", "It is not going to work"* or *"Why should I bother?".***

*However, as you start to do more activities, your mood and negative thoughts should lift, meaning that you will have more energy to do things. You may also find that your sense of pleasure and achievement after an activity increases too, so thinking about what you want to do and doing it will not feel so much of a challenge.*

***There is a very important reason why we have made the daily planner very detailed, such as including times of the day.***

*Have you ever heard of the phrase* ***"the road to hell is paved with good intentions?"****. It means that it is usually not enough to* ***intend*** *to do something good, as it is important that you* ***act*** *on your good intentions. Have you ever intended to do something good in the moment, but then later your motivation reduces, and you end up not doing it? I have. I have a shelf on my bookcase full of books that I planned to read but have never quite found the time.*

*In psychology we call this the* ***"intention-behaviour gap"****. The gap is the space between our intention, wishes or desires to do something, and the action of doing it. The bigger the gap, the more chance of us not acting on our intention. People with low mood can experience this a lot.*

*For example, it is common for people to have the thought,* ***"I'll do it in a bit"*** *and then before they know it, the day has gone. It can then feel like an ever-bigger task, especially as difficult feelings such as guilt and disappointment start to creep in.*

## *The good news is that we know what can help to close this gap.*

*In other words, we know how to increase the chance of someone doing the behaviour or activity. For example, if I say to myself by the end of the month, I want to have read one of the books that I have been putting off, it is likely that come the end of the month, the book will be in the exact same place where it is now, or I may have made a start but quickly given up on it. However, if I set myself a specific plan which focuses on* ***When, Where and How****, then I am more likely to have achieved my goal. For example:*

**WHEN:** I will read my book every other night at 8-9pm.
**WHERE:** I will read it in the back bedroom as there is a good reading lamp in there.
**HOW:** I will read at least 20 pages a night.

*The same rules apply for the daily planner. By making a plan in your daily planner of what you intend to do and when, you are more likely to close the intention-behaviour gap and do the activity.*

***Other things can also help to close this intention-behaviour gap, such as:***

- *Asking someone to do the activity with you so you share the responsibility of it getting done. This is one of the reasons why so many people find it more successful to exercise with someone else than if they did it alone.*
- *Use what you have learnt in this series on depression so far. For example, you know that by not doing the behaviour, it may fuel negative thoughts and emotions related with depression. Doing the activity however will help you to better manage your low mood.*
- *Make sure to give yourself helpful feedback for doing the activity such as praise, and celebrate your achievements. You may also want to ask a loved one for feedback to help give you some extra encouragement.*

TASK

## DAY FOUR

| Time | | Score |
|---|---|---|
| 6-7am | | P /10 A /10 M /10 |
| 7-8am | | P /10 A /10 M /10 |
| 8-9am | | P /10 A /10 M /10 |
| 9-10am | | P /10 A /10 M /10 |
| 10-11am | | P /10 A /10 M /10 |
| 11-12noon | | P /10 A /10 M /10 |
| 12noon-1pm | | P /10 A /10 M /10 |
| 1-2pm | | P /10 A /10 M /10 |
| 2-3pm | | P /10 A /10 M /10 |
| 3-4pm | | P /10 A /10 M /10 |
| 4-5pm | | P /10 A /10 M /10 |
| 5-6pm | | P /10 A /10 M /10 |
| 6-7pm | | P /10 A /10 M /10 |
| 7-8pm | | P /10 A /10 M /10 |
| 8-9pm | | P /10 A /10 M /10 |
| 9-10pm | | P /10 A /10 M /10 |
| 10-11pm | | P /10 A /10 M /10 |
| 11-12pm | | P /10 A /10 M /10 |

**Task**

## Day five

| | |
|---|---|
| 6-7am | P /10 A /10 M /10 |
| 7-8am | P /10 A /10 M /10 |
| 8-9am | P /10 A /10 M /10 |
| 9-10am | P /10 A /10 M /10 |
| 10-11am | P /10 A /10 M /10 |
| 11-12noon | P /10 A /10 M /10 |
| 12noon-1pm | P /10 A /10 M /10 |
| 1-2pm | P /10 A /10 M /10 |
| 2-3pm | P /10 A /10 M /10 |
| 3-4pm | P /10 A /10 M /10 |
| 4-5pm | P /10 A /10 M /10 |
| 5-6pm | P /10 A /10 M /10 |
| 6-7pm | P /10 A /10 M /10 |
| 7-8pm | P /10 A /10 M /10 |
| 8-9pm | P /10 A /10 M /10 |
| 9-10pm | P /10 A /10 M /10 |
| 10-11pm | P /10 A /10 M /10 |
| 11-12pm | P /10 A /10 M /10 |

Task

| Day six | |
|---|---|
| 6-7am | P /10 A /10 M /10 |
| 7-8am | P /10 A /10 M /10 |
| 8-9am | P /10 A /10 M /10 |
| 9-10am | P /10 A /10 M /10 |
| 10-11am | P /10 A /10 M /10 |
| 11-12noon | P /10 A /10 M /10 |
| 12noon-1pm | P /10 A /10 M /10 |
| 1-2pm | P /10 A /10 M /10 |
| 2-3pm | P /10 A /10 M /10 |
| 3-4pm | P /10 A /10 M /10 |
| 4-5pm | P /10 A /10 M /10 |
| 5-6pm | P /10 A /10 M /10 |
| 6-7pm | P /10 A /10 M /10 |
| 7-8pm | P /10 A /10 M /10 |
| 8-9pm | P /10 A /10 M /10 |
| 9-10pm | P /10 A /10 M /10 |
| 10-11pm | P /10 A /10 M /10 |
| 11-12pm | P /10 A /10 M /10 |

**Task**

## Day seven

| | | |
|---|---|---|
| 6-7am | | P /10 A /10 M /10 |
| 7-8am | | P /10 A /10 M /10 |
| 8-9am | | P /10 A /10 M /10 |
| 9-10am | | P /10 A /10 M /10 |
| 10-11am | | P /10 A /10 M /10 |
| 11-12noon | | P /10 A /10 M /10 |
| 12noon-1pm | | P /10 A /10 M /10 |
| 1-2pm | | P /10 A /10 M /10 |
| 2-3pm | | P /10 A /10 M /10 |
| 3-4pm | | P /10 A /10 M /10 |
| 4-5pm | | P /10 A /10 M /10 |
| 5-6pm | | P /10 A /10 M /10 |
| 6-7pm | | P /10 A /10 M /10 |
| 7-8pm | | P /10 A /10 M /10 |
| 8-9pm | | P /10 A /10 M /10 |
| 9-10pm | | P /10 A /10 M /10 |
| 10-11pm | | P /10 A /10 M /10 |
| 11-12pm | | P /10 A /10 M /10 |

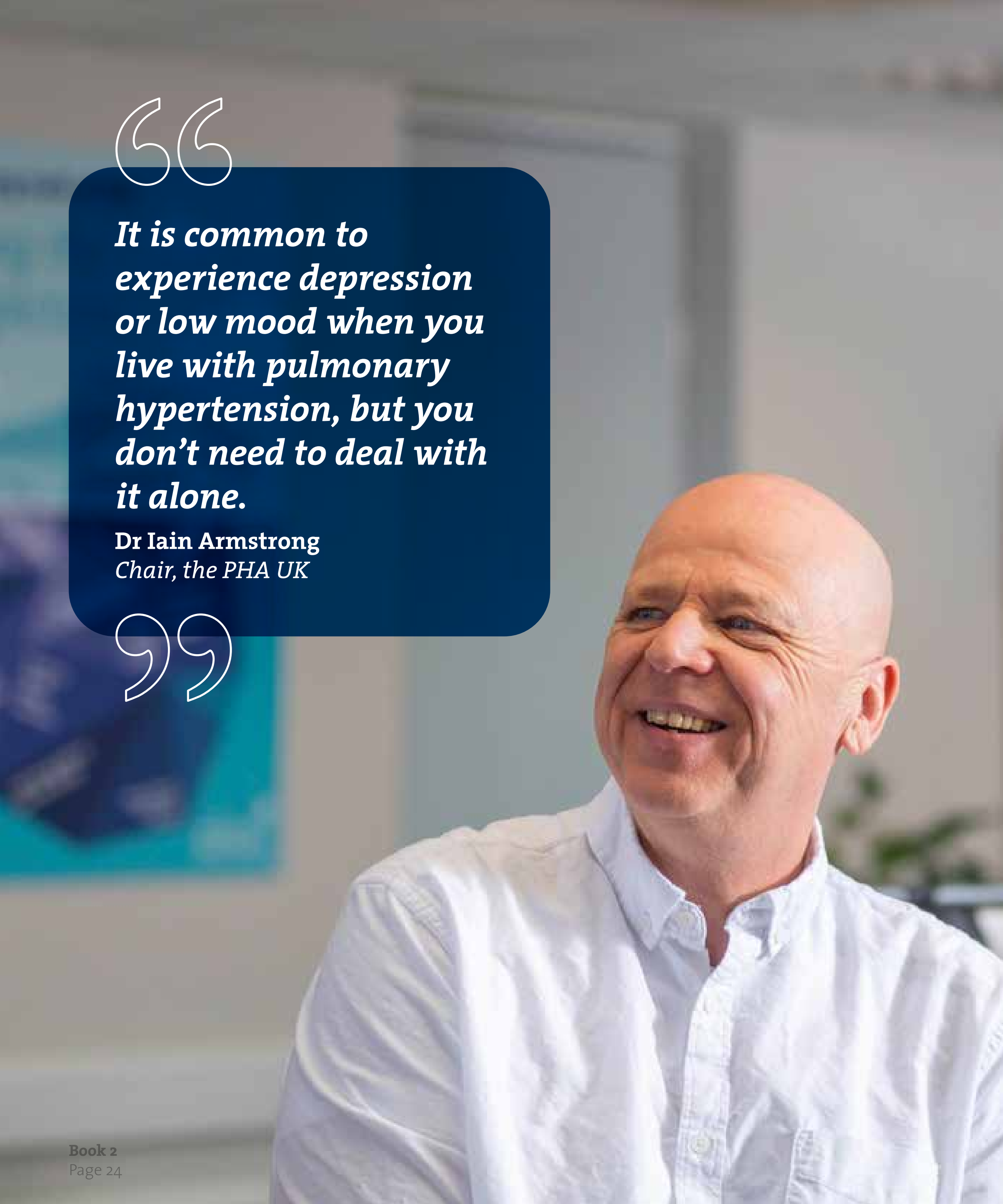

*It is common to experience depression or low mood when you live with pulmonary hypertension, but you don't need to deal with it alone.*

**Dr Iain Armstrong**
*Chair, the PHA UK*

# ***Positive*** activities

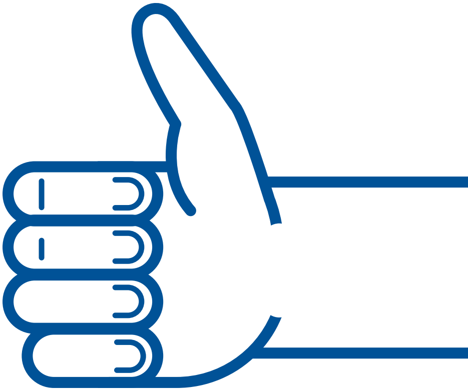

*We know people like different things, but here is a list of possible activities that you could try. You don't need to do everything, so start by picking one. You will probably like some ideas more than others, so try a variety.*

## *Feel free to add your own..!*

- *Have a bath or shower*
- *Collect things*
- *Go on a day trip*
- *Go to the movies*
- *Watch a movie at home with someone*
- *Go to the beach*
- *Go to the countryside*
- *Play a sport*
- *Have a BBQ*
- *Bake something nice*
- *Cook something nice*
- *Listen to some music*
- *Do some light exercise*
- *Go some gardening*
- *Go for a walk*
- *Text a friend*
- *Write to friend*
- *Speak to a friend*
- *Listen to a friend*
- *Arrange to meet a friend*
- *Arrange a date night*
- *Join an online group*
- *Practice mindfulness*
- *Light a candle*
- *Read a book*
- *Do the washing up*
- *Hoover a room*
- *Make yourself a cup of tea*
- *Make yourself a snack*
- *Go shopping*
- *Go to a café*
- *Tidy something in your house such as a kitchen drawer*
- *Play a game*
- *Practice a hobby*
- *Learn a new skill*
- *Make something*
- *Look at old photos or videos*
- *Make your bed*
- *Look after your personal hygiene such as brushing your teeth*
- *Put the washing on*
- *Put the washing away*
- *Make yourself a hot water bottle*
- *Give a loved one a cuddle or say something nice to them*
- *Open up to a friend*

**Add your own...**

+ __________
+ __________
+ __________
+ __________
+ __________
+ __________
+ __________
+ __________

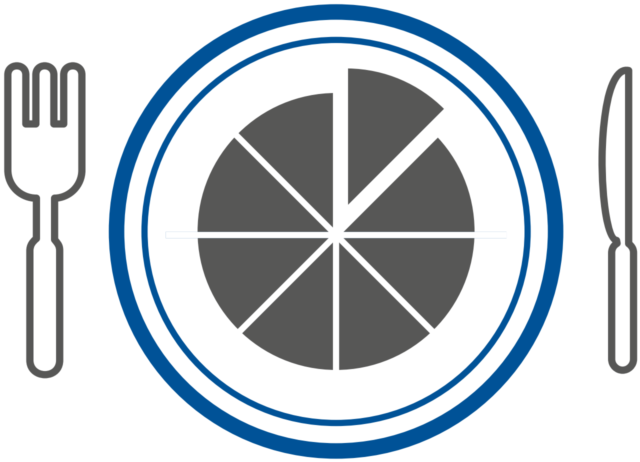

# Breaking activities up

***When thinking about what you want to do, it is important to break up activities into smaller and more achievable goals.***

This is because due to your depression and PH, you may not be doing many activities so building up your level of activity is a good idea. Also, people with depression can be hypervigilant or more aware of their own failure. Their mood can reduce very quickly due to a setback, sometimes more than someone without depression. In other words, if you set yourself a task and you are unable to do it, it is likely to have a big impact on your mood. Another name for this is ***"one step forward and two steps back"***.

As you saw in the video last week describing depression as a big black dog, how you respond to setbacks will get easier over time as you start to recover from depression and engage in more helpful ways of coping. However, it is important to consider where you are now in your journey to recovery.

# Other helpful things to consider...

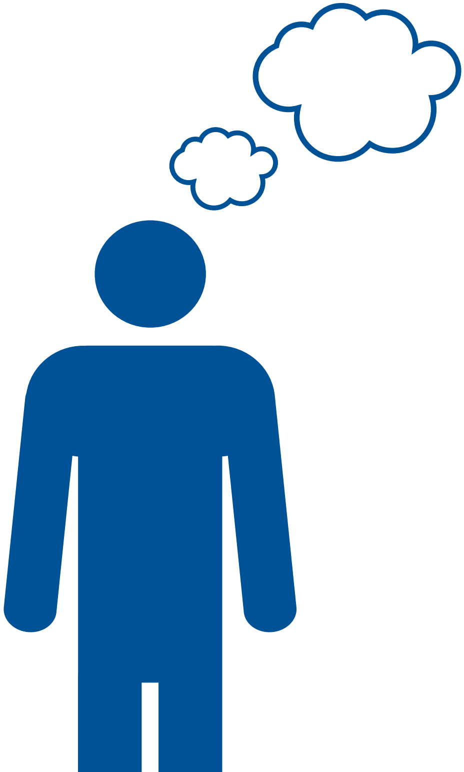

## Get into a routine

*Our bodies love it when we have a routine. Getting into a regular routine is important for our mood, health, sleep and appetite, among other things. You should aim to keep to a usual time to wake up, go to bed, when to eat large meals such as breakfast, lunch and dinner, and get dressed. For example, if you normally wake up at 8am but one morning you feel tired, you should still get up and start your day at 8am. This is because if you sleep in, later that day you will feel less tired meaning you go to bed later and wake up later and so on. Even though this can be hard, like we discussed in the previous book, follow your plan and not your mood.*

## Have enough sleep

*Depression can have a negative impact on the amount and quality of our sleep. Getting enough sleep is very important for our mental and physical health. We all know the difference in how we feel after we have had a good night's sleep and after a bad night's sleep. There are certain habits that we can do to help improve our sleep. These include:*

- *Work out how much sleep you need. The average is between 7-9 hours of sleep per night for an adult.*
- *Avoid screens such as phones, TV and reading devices at least 30 minutes before bedtime.*
- *Limit daytime naps and do not nap for longer than 20 minutes.*
- *Only use your bed for sleep and sex, for example, do not lay in your bed watching movies or TV shows.*

## Keep mentally and physically active

*Keeping active is important for your physical and mental health. Mental exercise is important for mood and confidence. Physical exercise will help to improve your mood, lower your anxiety and boost your confidence. As you read earlier, doing activities will also help to manage fatigue and other symptoms of PH, such as breathlessness. Remember, not all pain is bad, as discomfort can be caused by stretching muscles and nerves that have become tight due to little use. As we discussed in the previous book, when you are tired it can be helpful to rest to recover, but in depression, some rest can actually make you feel worse!*

# Summary

In this second book you learnt about the importance of replacing inactivity with activity. This is one of the most effective ways of managing depression. We explored some of the do's and don'ts of this skill, such as not doing too much but also not doing too little. Breaking up bigger tasks into smaller ones will make it more achievable and give you a sense of accomplishment and confidence. Remember, it is not a race or competition to achieve your goal. Instead, go at your own pace, being aware of any unhelpful patterns that might be feeding your depression.

We recognise that managing depression is not just about doing more things. Next week we will be exploring how thinking patterns can maintain your depression and help you develop more coping skills.

*Please remember, Abbie Stark (Trainee Clinical Psychologist from Cardiff University) may be contacting you this week via phone or email to see how you are getting on with these booklets. She will be asking you a few questions about your experience of the booklets so far.*

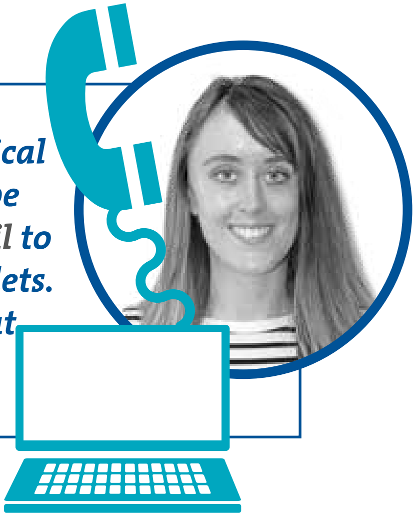

# Exercise:

## Replacing inactivity

Over the next week, your homework is to start completing your activity scheduling. This task is so important that you will be asked to complete a new activity schedule in booklets three and four.

*Some final things to think about over the next week:*

***Small steps can have a large impact on how we feel. Also, small setbacks can have a large impact too, so take things on that are manageable.***

***As we will explore more in booklet four, it is not the case that engaging in ANY activity will make you feel better. It is important to do things that trigger more positive feelings such as mastery, pride, sense of success and achievement, and compassion.***

***"It's not what you do or how fast, but that you do it!" Remember to praise yourself when you do these things.***

***As we saw in the previous book, Catherine made a list of the things she has neglected and needs to do, which made her feel overwhelmed. This shows the importance of breaking down activities. If your kitchen needs cleaning, first start with the dishes and take a break before starting on the work bench, even if you do not feel you need to rest.***

***Don't overthink doing an activity as your negative thoughts might take over and affect your motivation. Follow your activity schedule.***

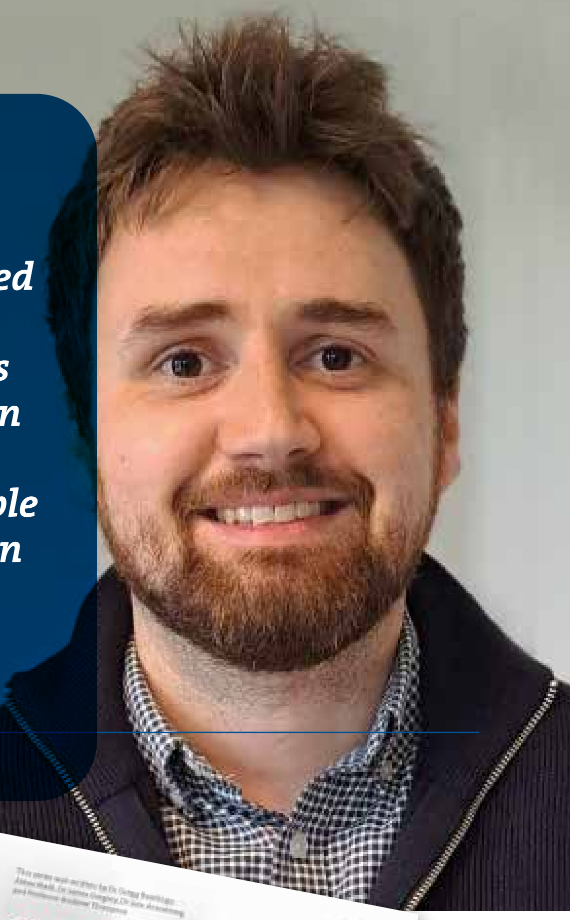

*I hope that you find that these strategies can help you to better manage your experiences associated with depression. By taking part in this project, you are also helping us to learn more about how depression interacts with PH. We can use this information to better support people who are affected by the condition in the future. Thank you.*

**Dr Gregg H. Rawlings**
*Clinical Psychologist*
*Nottingham Trent University*

In the next booklet, we will be looking at how depression can impact on our thoughts, which in turn can then make it more likely that we experience symptoms of depression.

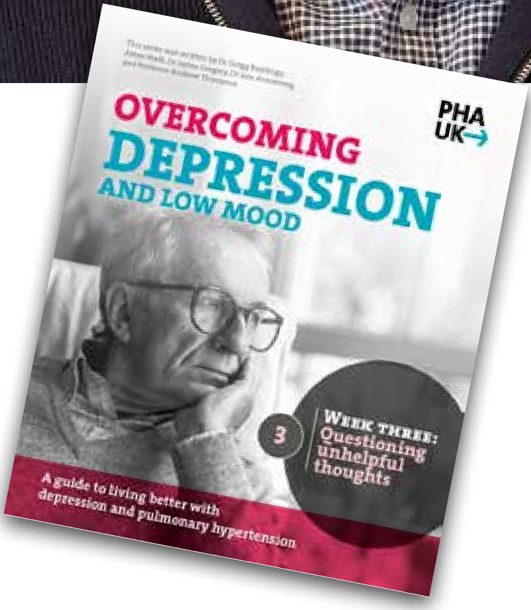

# *Additional resources*

*If you feel that you need additional information or support, please contact your GP or the PHA UK for advice. We have also included a list of other helpful resources:*

**Overcoming Worry & Anxiety**
*(a self-help programme for people with PH)*
www.bit.ly/OvercomingWorryAndAnxiety

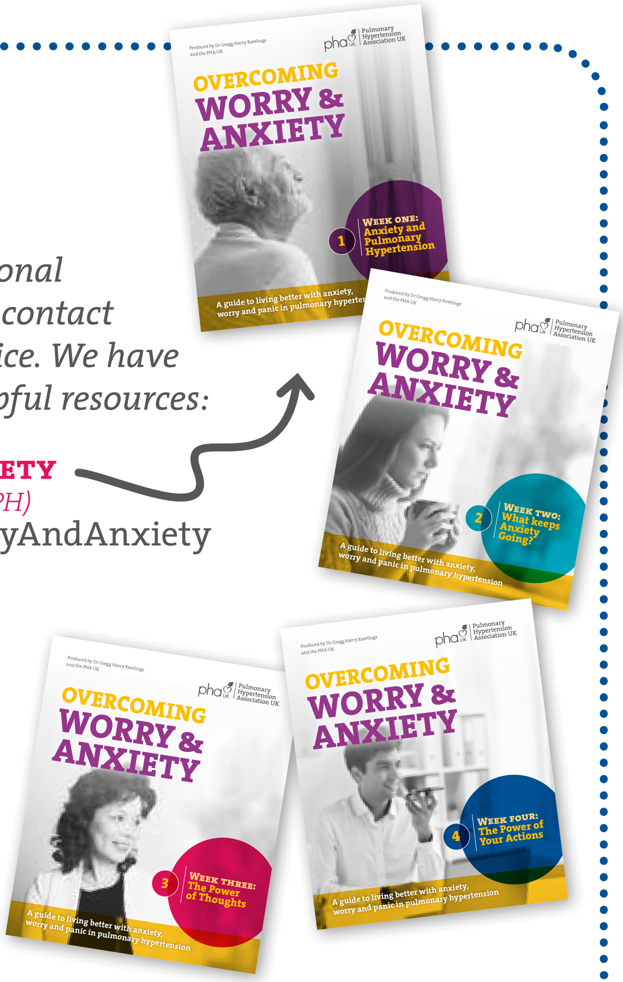


**NHS self-help**
www.bit.ly/NHSSelfHelp

**PHA UK**
www.phauk.org

**Depression UK**
www.depressionuk.org

**Samaritans**
www.samaritans.org

**Mind**
www.mind.org.uk

**! Thoughts of self-harm and suicide can also be common signs of depression. If you start to experience any of these symptoms, please speak to a healthcare professional.**

The current study has been designed by researchers working in the UK and it has received ethical approval from an academic institution in the UK (Cardiff University). Participants should be aware and act in accordance with information and governance associated with their country. If you have any questions, please contact your healthcare professional.

For references, see Book 4.

*It is just as important to treat the mind as it is the body.*

**Wendy Gin-Sing**
*Pulmonary Hypertension Nurse Consultant*

To find out more about the PHA UK, visit **www.phauk.org**


***Pulmonary Hypertension Association UK***

PHA UK Resource Centre, Unit 1, Newton Business Centre, Newton Chambers Road, Thorncliffe Park, Chapeltown, Sheffield, England S35 2PH

**T:** 01709 761450 **E:** office@phauk.org @PULHAUK @PHA_UK @PHA ORG UK



Registered Charity No. 1120756. Copyright © 2023 PHA UK. Version 1.2.
