# Supplementary material for: A randomized controlled trial of self‐help cognitive behavioural therapy for depression in adults with pulmonary hypertension
Source: Br J Health Psychol. 2025 Jun 12;30(3):e12800. doi: 10.1111/bjhp.12800 (PMC12159717; doi:10.1111/bjhp.12800)
Supplement: Supplementary file 6 — Data S6. [file BJHP-30-0-s001.pdf]

This series was written by Dr Gregg Rawlings,
Abbie Stark, Dr James Gregory, Dr Iain Armstrong
and Professor Andrew Thompson

PHA UK

OVERCOMING DEPRESSION AND LOW MOOD

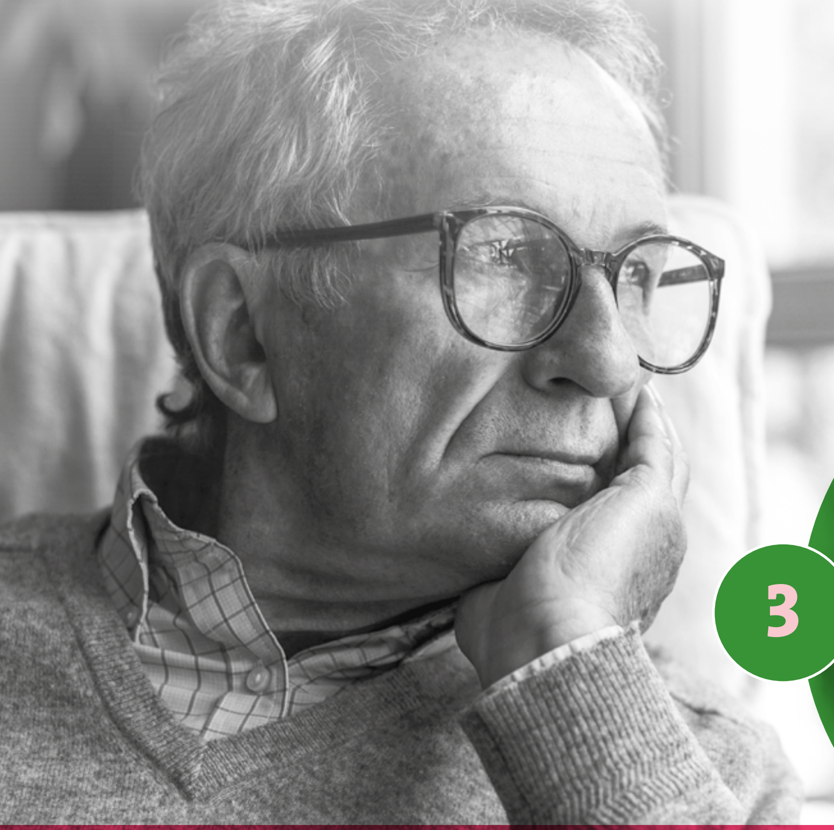

3

Week three: Questioning unhelpful thoughts

A guide to living better with depression and pulmonary hypertension

PHA UK

Pulmonary Hypertension Association UK

Week three: Questioning unhelpful thoughts

Overcoming depression and low mood

Here is a summary of what will be covered in this booklet:

- *We have a tendency to see thoughts as facts, which can actually feed into our depression*
- *Explore the different types of unhelpful thinking styles and understand how 'negative thoughts' can affect people with PH*
- *Develop strategies to help recognise and tackle unhelpful and negative thoughts and thinking styles*

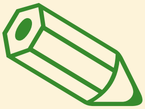

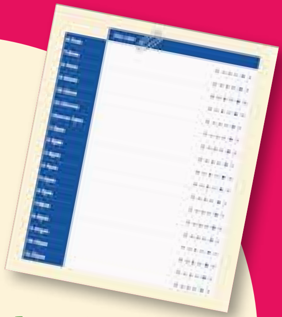

Your week three activity diary starts on page 36, remember to complete it as you work through this booklet.

Questioning **unhelpful** thoughts

This week we will be looking at how depression can impact on our thoughts, which in turn can then make it more likely that we experience symptoms of depression.

You might have noticed that depression can act like a bully as it can make our thoughts really negative. If we listen to and believe this bully, we can understandably be hurt by it. The chance that we end up believing this bully is increased because we often treat thoughts as facts. It's human nature.

We have all been in situations where we have had a negative thought and we are certain that it will come true, or we have the same emotional reaction as if it was true.

It is important to remember that thoughts are not facts.

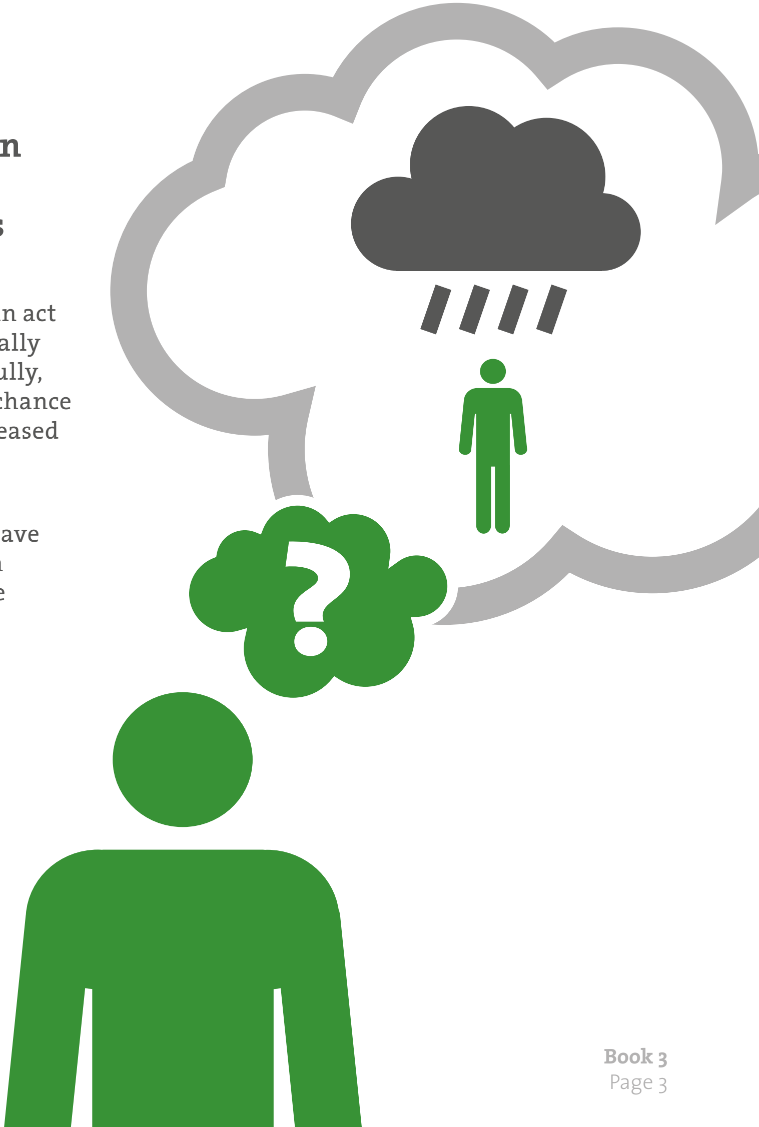

See an example of how we tend to treat thoughts as facts here.
Just type the link below into your web browser.

www.bit.ly/ThoughtsAreNotFacts

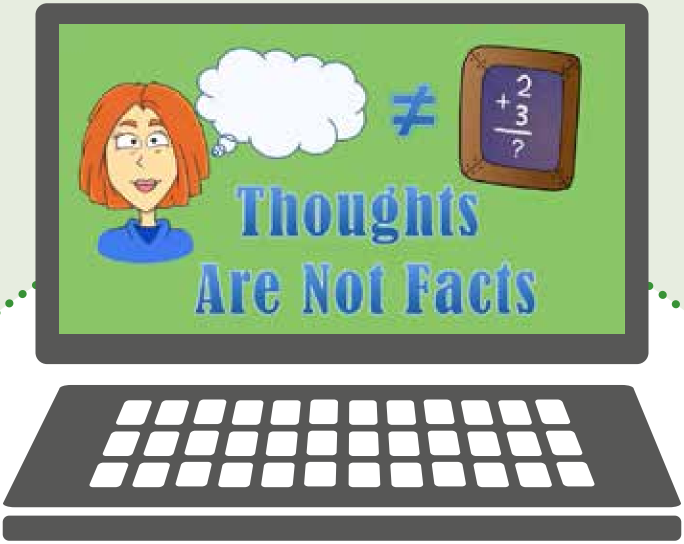

You may want to write down some of your thoughts and reflections from watching the video.

Depression can start off in a small dose; however, the longer someone feels low, the louder the depression bully will become and the more likely we will listen to it. This can result in us getting stuck in unhelpful thinking patterns and behaviours. This means that our low mood becomes even lower and more persistent.

Over time we can stop using ***unhelpful*** ways of coping and start to engage more in ***helpful*** behaviours and thoughts. This can happen without us even realising we are doing it.

This is known as a vicious cycle, and an example of it is shown below:

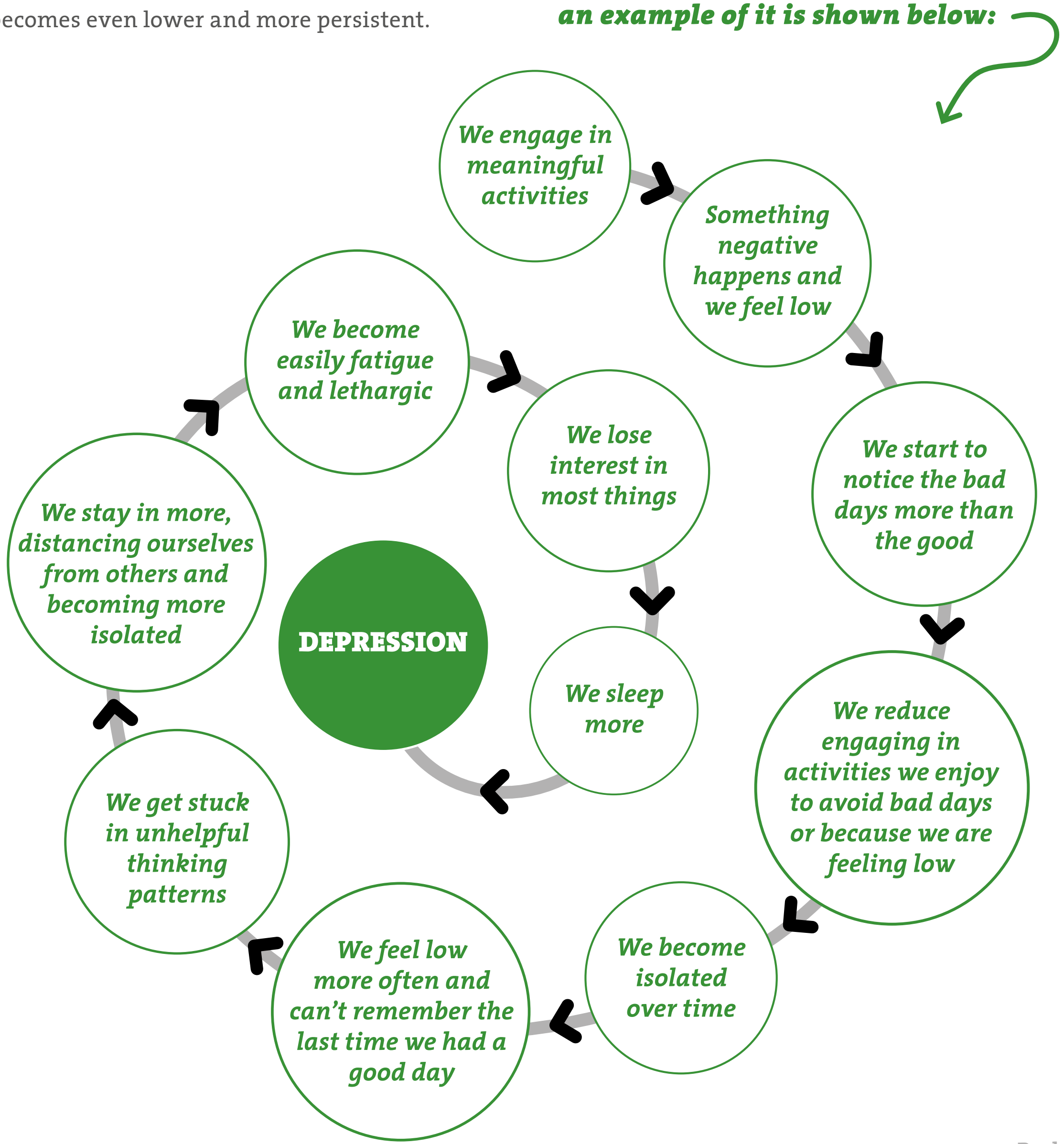

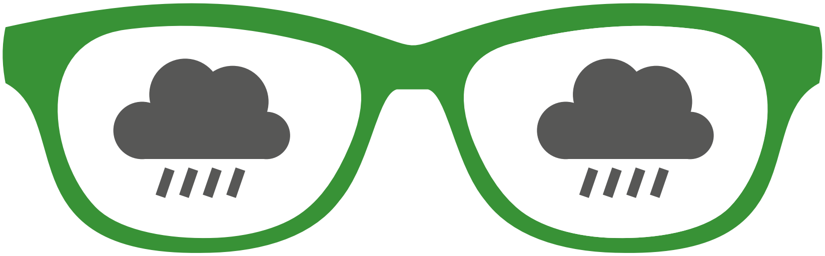

As we discussed in the first week, the way we view, understand and remember events can all be negatively influenced by depression.

For example, we may be more likely to notice things that are consistent or support our negative views. For example, when Ally's partner tries to talk to him about his low mood, Ally sees it as she is trying to interfere or is 'nagging', as opposed to trying to help him. We may also be more likely to interpret information negatively as well as remember negative events, just like Catherine did when she was remembering the day at the theme park where she was unable to join her grandchildren on the rides. Unfortunately, this is how our brains work, but this booklet looks at how we can challenge this.

One way to think of this focus on negativity is to imagine that you put on a pair of glasses which filters everything you see. Unlike 'beer goggles', these glasses make everything look negative. When you look in the mirror with these glasses you see yourself, but in a negative way – such as focusing on the things you dislike about yourself, or you may start to call yourself self-critical words. This may then fuel some of your self-negative thoughts, lowering your mood further.

When you walk down the street with these negative glasses on, instead of thinking about what you want to do or being mindful, you think about what you must do and the pressure that comes along with this. Rather than noticing the nice weather, scenery or people in the street, you look down at the floor and start to worry about how far you can walk before feeling pain. Then, as if out of nowhere, you start to experience pain that you didn't notice before. This is quickly followed by feelings of anger, sadness and anxiety, and you worry how far you can continue to walk .

Using the diagram you completed in week one, which is shown below, you know that this cycle between your thoughts, emotions, physical sensations and behaviours will keep going – driving you deeper into depression if it is not challenged.

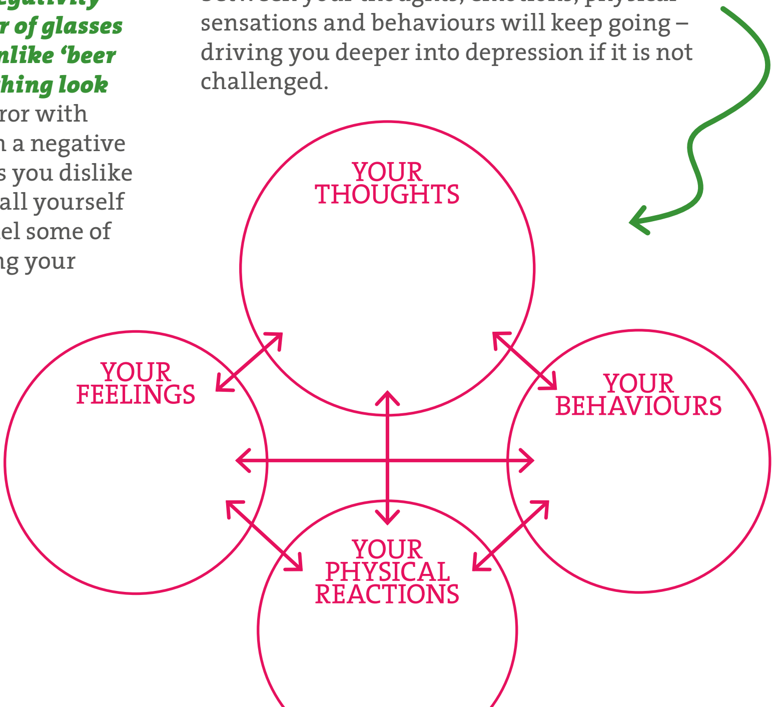

Depression and anxiety in people with PH can be associated with unhelpful thoughts (cognitions) and behaviours, such as judging yourself negatively, using avoidance as a way of coping, or withdrawing from activities that are important to you. This makes a lot of sense and can apply to many people, whether they are living with a long-term condition or not.

Dr Gregg H. Rawlings
Nottingham Trent University
Developer of *A guide to living better with depression and pulmonary hypertension* and *A guide to living better with anxiety, worry and panic in pulmonary hypertension*

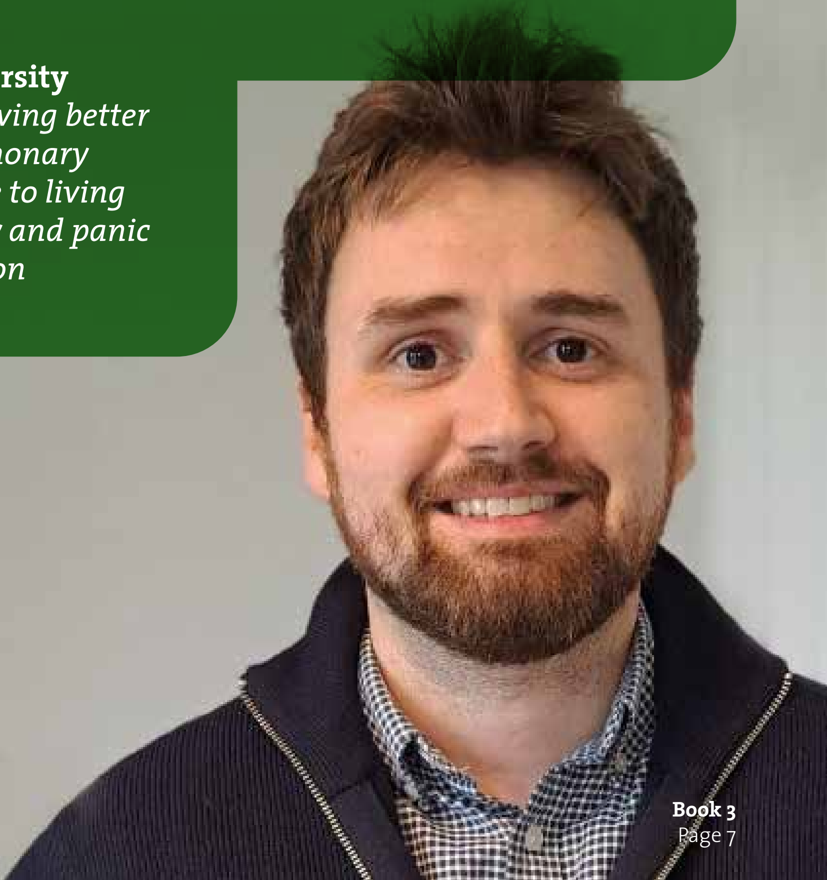

Examples of unhelpful thinking...

In this booklet we are going to explore some of the ways depression affects how we think. Noticing your unhelpful thinking patterns is an important step in learning how to manage your depression.

We all have unhelpful thinking patterns, not just people with PH or people with depression. On the next six pages are common examples of unhelpful thinking patterns people with PH may experience. Please read and tick those that apply to you, and add in any of your own examples.

Predicting the future...

People can jump to a negative conclusion about situations in the future. For example, ***"I will never feel better"***, ***"I will always have these difficulties"***, ***"There is no point"***, ***"I will end up not enjoying myself"***, ***"Everyone will think I am weird"*** and ***"People will ask me questions about my condition, which will just make me upset"***. More helpful thoughts could be: "***I can't be sure what I think will happen will"***, ***"I was wrong before when I thought something bad would happen, I might be wrong this time as well"*** and ***"If that happens, I will just deal with it"***. It is likely to be difficult to change these negative thoughts at first, but with time and practice you will begin to believe the new, more helpful thoughts.

SITUATION
Receiving a letter for an appointment at the hospital

Unhelpful thoughts

THOUGHTS
"It is going to be bad news", "My PH will have got worse", "I won't be able to cope"

EMOTION
Sadness, anxiety, low mood

OUTCOME
You avoid talking about it with others despite your family asking how you are feeling. This makes you feel low, and you find that you are distancing yourself from others, when really you want to feel supported

Helpful thoughts

THOUGHTS
"I don't know what will happen, but every time I have seen my doctor about my PH, I have felt supported", "Just because I think it is bad news, doesn't mean it will be – my thoughts are not facts"

EMOTION
Less scared, not feeling as low as you did when you first opened the letter

OUTCOME
You make a plan for that afternoon as you know activities can help to lift your mood

Does this pattern apply to you? *Oh yes! that's me* ☐ *No, not really* ☐

Any thoughts of your own on similar situations?

There's more space on page 21

Another example of
unhelpful thinking...

Mind reading...

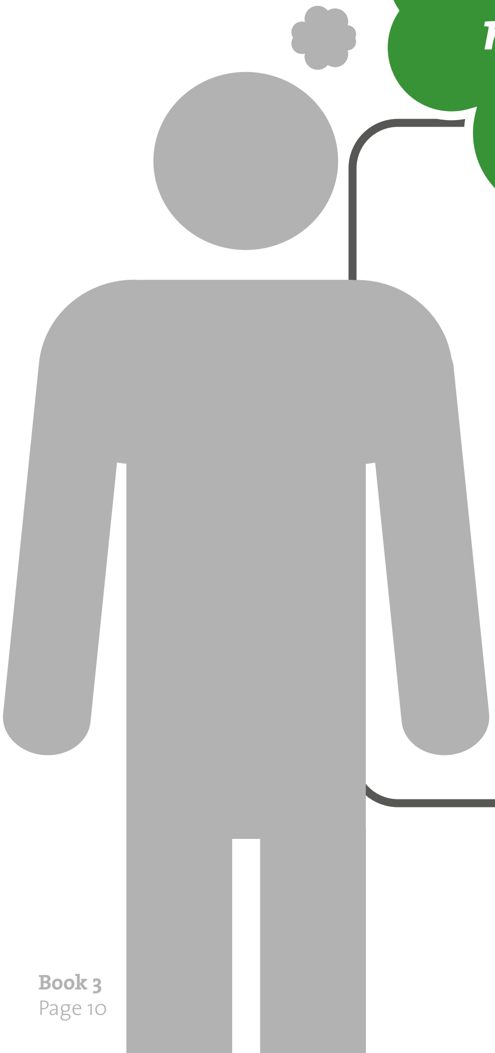

People can worry what other people are thinking. For example, ***"That person is thinking I am pathetic"*** and ***"I bet my friends think I am a burden now because we cannot do the things we used to"***. More helpful thoughts could be ***"I don't actually know what they are thinking"*** and ***"They will have their own things to worry about rather than judging me"***.

Situation
Friends are planning a night away

Unhelpful thoughts

Thoughts
"My friends will think I'm holding them back", "They'll wish I didn't go"

Emotion
Anger, sadness, anxiety

Outcome
In the end you go but stay in your room most of the time. When you do go out, you focus on any signs of PH and are constantly wondering what your friends are thinking about it, which means you don't enjoy yourself.

Helpful thoughts

Thoughts
"My friends like me for me" "If I explain how PH affects me, they will be more understanding"

Emotion
Motivated, slightly excited, slightly anxious.

Outcome
You go and get a sense of achievement. While you experience some PH symptoms, you cope with it like always and your friends say that they were glad you went.

Does this pattern apply to you? *Oh yes! that's me* ☐ *No, not really* ☐

Any thoughts of your own on similar situations?
There's more space on page 21

Another example of
unhelpful thinking...

Over-generalisation...

People view a single negative event as affecting everything. For example, ***"I didn't enjoy going out the last time so I won't enjoy myself this time"***, ***"I couldn't do it last week so what makes you think I could do it now?"*** and ***"I struggled to catch my breath in the supermarket, which means it will always happen now when I go out"***. More helpful thoughts could be: ***"Just because I found it difficult before doesn't mean I will this time and besides, it is important I keep trying"*** and ***"It doesn't really mean anything that I had difficulty, it doesn't mean it will definitely happen again but even if it does, I will manage it like I did before"***.

SITUATION
Going out for a drink to a bar

Unhelpful thoughts

THOUGHTS
"I know my PH will flare up like the last time I went to the bar and I didn't have a good time"

EMOTION
Anger and sadness

OUTCOME
In the end you go out but keep making plans in the hope it will prevent your PH symptoms. For example, "We can't go there because there is no lift or we can't go there because we will not get any seats and I will soon tire'. This means you don't enjoy yourself. You leave after one drink. You think about this experience in the future when asked if you want to go and it makes you want to say no.

Helpful thoughts

THOUGHTS
"That one experience I had where I didn't enjoy being at the bar was a one-off, this time it might be different"

EMOTION
Motivated, slightly excited, slightly anxious

OUTCOME
You go and have a better time than that one-off bad experience.

You realise that if you let the bad experience put you off, you would have missed out.

Does this pattern apply to you? *Oh yes! that's me* ☐ *No, not really* ☐

Any thoughts of your own on similar situations?

There's more space on page 21

Another example of **unhelpful thinking...**

All or nothing thinking...

People can only see the extremes and not the grey areas. For example: ***"I wanted to do that activity but couldn't, so I am failure"***, ***"It didn't work out because I was involved, if someone else was doing it, it would have been brilliant"***, or ***"I feel guilty that my partner has to do everything for me"***.
More helpful thoughts could be: ***"I may struggle with stairs now and again but at least I am able to do it"*** and ***"Although I cannot do some things, I am still able to do a lot and I now have more time to support my partner in other ways"***.

SITUATION
Going out shopping

Unhelpful thoughts

THOUGHTS
"I can't do a full shop to last us a week like I used to be able to"

EMOTION
Sadness, frustration

OUTCOME
You leave the shopping to someone else and feel "useless" at home. You follow your thoughts and feelings rather than your scheduling of activities.

Helpful thoughts

THOUGHTS
"I will go shopping and be mindful not to get too many items so that I am able to carry it"

EMOTION
Motivated, a little anxious, pleased with yourself you're doing something that will give you a sense of accomplishment

OUTCOME
You go shopping and feel a sense of achievement for getting food for tonight's meal. You use your coping skills, such as mindfulness, to help control your anxiety.

Does this pattern apply to you? *Oh yes! that's me* ☐ *No, not really* ☐

Any thoughts of your own on similar situations?
There's more space on page 21

Another example of ***unhelpful thinking…***

"SHOULD" and "MUST" statements…

You can have strict and often unrealistic ideas about what you should or must do and are very judgmental if they are not met. For example: ***"I must be able to keep my house tidy"***, ***"I should be able to support my own family"***, ***"I should be able to do it"***. More helpful thoughts could be: ***"I may not be able to keep my house the way it was before but it is still tidy"***, ***"Although I would like to do it, I need to recognise my limits and not beat myself up over it"***.

Situation
Your partner suggests you go out together on a date on Friday night

Unhelpful thoughts

Thoughts
"I should be able to go out on date, but I know I will feel self-conscious about having to use my medication in public"

Emotion
Frustration, disappointment, anger

Outcome
Say yes but then change your mind last-minute explaining you do not feel well. You experience self-bullying thoughts for the rest of your evening

Helpful thoughts

Thoughts
"I would like to go and although I feel nervous, I know what I am worrying about might not happen" and "This is a positive step in managing my low mood"

Emotion
Slightly anxious but excited

Outcome
You use your coping skills to challenge your negative thoughts but it is becoming a bit too much. You tell your partner your concerns, who listens and reassures you. You go and use your mindfulness skills to manage your initial anxiety. Once you have ordered drinks, you stop listening to your bullying thoughts and listen to your partner instead.

Does this pattern apply to you? *Oh yes! that's me* ☐ *No, not really* ☐

Any thoughts of your own on similar situations?
There's more space on page 21

Another example of **unhelpful thinking...**

Self-criticism...

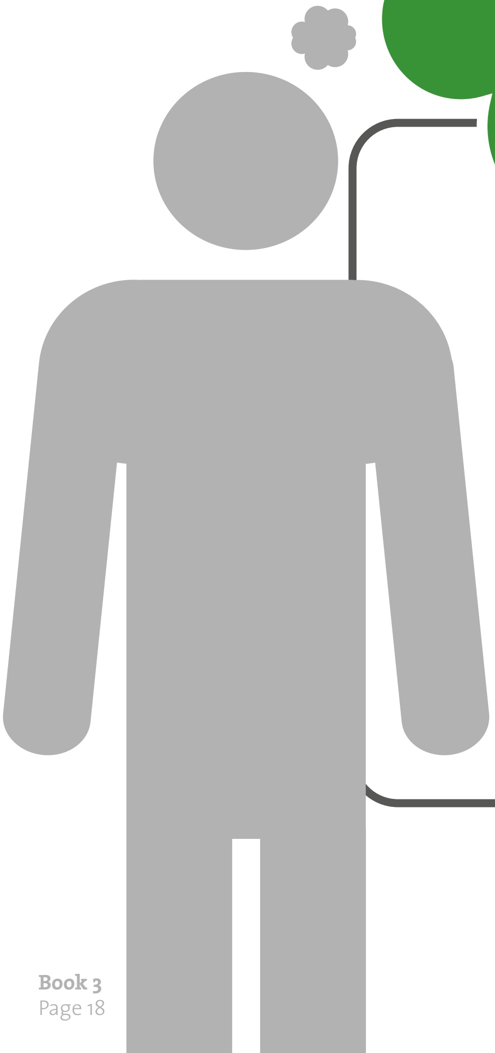

You call yourself critical names or words. People can fall into patterns of self-bullying and have negative thoughts about themselves. For example: ***"I am useless"***, ***"I am boring"***, ***"It is my fault and I deserve to feel this way"*** or ***"Why me, I used to be able to do all those things and now I can't do anything"***. More helpful thoughts could be: ***"Everyone has something different about them"***, ***"No one is perfect"*** and ***"I am putting myself down"***.

SITUATION
PH is preventing you from doing something important in your life

Unhelpful thoughts

THOUGHTS
"Why does it have to be me with PH", "I am so useless", "It is all because of me and it is my fault"

EMOTION
Anger, frustration, shame, guilt and depression

OUTCOME
You bottle your emotions up and while this helps in the short-term, in the long-term it means the emotions become stronger and more negative. You isolate yourself from others for fear they will ask you a question that will cause you to feel upset.

Helpful thoughts

THOUGHTS
"I know it is not my fault", "I have every reason to be annoyed and upset at the situation", "I know beating myself up over it will not help and will just make me feel depressed"

EMOTION
Sadness at the situation, hurt

OUTCOME
You do something to take care of yourself even though you do not feel like it at first, you talk to your partner. While the situation does not change the situation, it changes how you feel as you feel listened to and loved.

Does this pattern apply to you? *Oh yes! that's me* ☐ *No, not really* ☐

Any thoughts of your own on similar situations?
There's more space on page 21

Another example of **unhelpful thinking...**

All or nothing thinking continued...

People can also use all or nothing thinking to understand the behaviour of others. For example: ***"My partner should know I cannot do that, he is always getting things wrong"*** and ***"My doctor said my tests were unclear, she is useless"***. More helpful thoughts could be: ***"My partner gets most things right and he didn't mean to upset me"*** and ***"My doctor has arranged for more tests and explained she will speak to her colleagues about my symptoms"***.

Any thoughts of your own on similar situations?

There's more space opposite

Space to make further notes from pages 9 to 20 about your unhelpful thinking patterns...

Challenging unhelpful thoughts

The first step to managing your unhelpful thinking patterns is to spot the thought. As you may have noticed by now, for some of us this can be tricky.

As we discussed in booklet one, sometimes it can be helpful to think, ***“if my mind was on a TV screen, what would other people see?”***. This might help you to notice what is on your mind. Some people find that they have images in their mind rather than thoughts, therefore drawing an image about what is on your mind might be helpful.

When you have identified the negative thought, the next step is to challenge it. You have already seen examples of this in the previous exercise, showing an unhelpful and helpful reaction to the same situation.

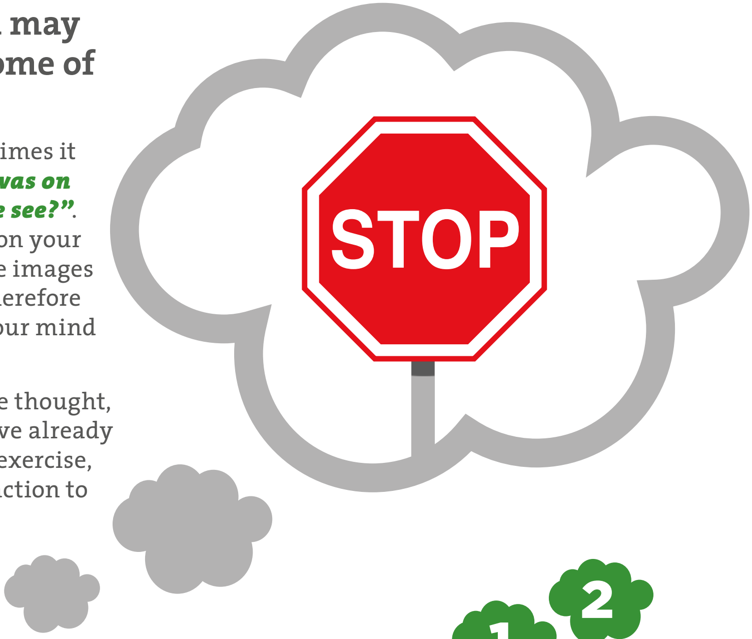

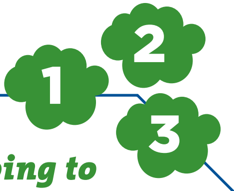

We are now going to look at three ways of managing unhelpful thoughts:

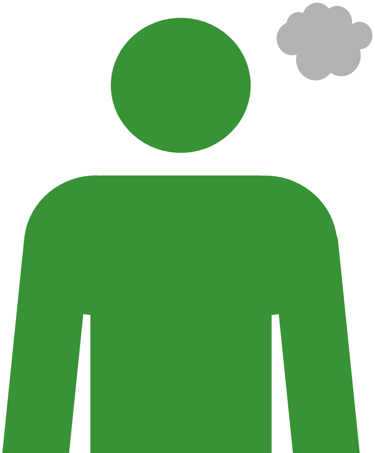

1 Challenging unhelpful thoughts...

When we are challenging our negative thoughts, we should remember to be fair and accurate to ourselves.

However, the **'depression bully'** can make this difficult to do. The bully can sometimes make us be unkind and uncompassionate towards ourselves and be overly judgemental. Question your thoughts and ask yourself ***"am I being fair to myself here?"***. Questioning unhelpful thoughts can help you to identify more helpful alternatives, improve your mood, and help you with other difficulties that you may be experiencing – such as anxiety.

You can continue to question your negative thought by asking yourself some of these questions:

*What is the **evidence** for and against the thought?*

*Have I had any **experiences** that would suggest this thought may not be true?*

*What would I say to a **friend or relative** that I care about who is having this thought?*

*What would a friend or relative who cares for me say to me if I told them I was having this thought? **Would they agree with me?***

*Is there a way to look at the situation **differently** and can I problem solve to address the issue?*

*Am I falling into an unhelpful **thought pattern**? For example, am I jumping to conclusions? Am I being overly self-critical?*

*Is the thought helpful or will it just keep my depression going? This is particularly important as many people hold positive views about repetitive negative thinking. For example, sometimes we think worrying about something is good because **"It helps me problem solve", "It makes me be a better person"** or **"It makes me feel prepared"**. While some of this may be true, endless negative thinking or not being able to stop worrying is not helpful.*

Recognising, challenging, or letting go of your unhelpful thoughts can be difficult at first, but with time it becomes easier as you engage less in them.

For example, Catherine begins to challenge her thought that...

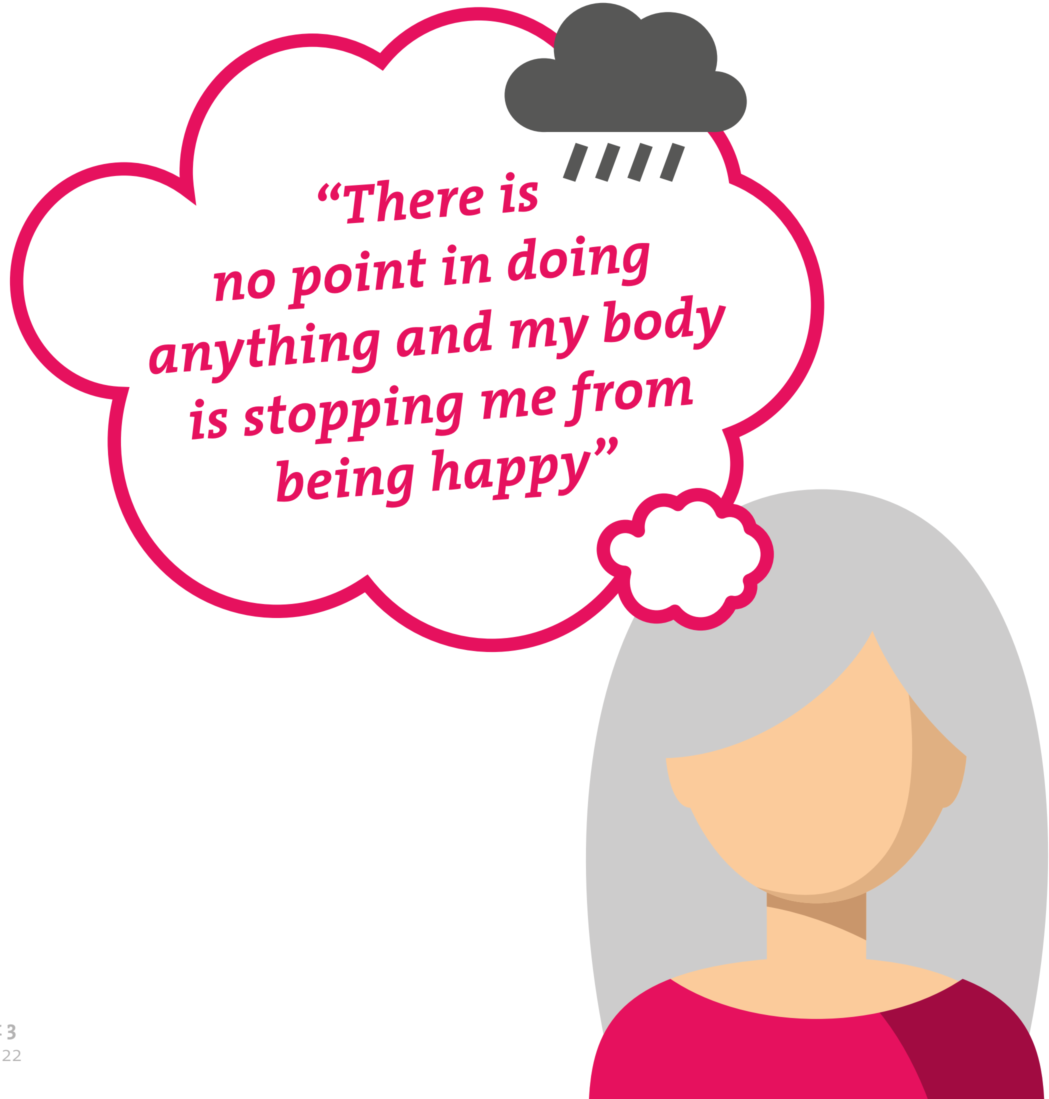

Evidence for this thought	Evidence against
I hate my body and when I look in the mirror it's a constant reminder it is failing me	*It is the only body I have and it's not its fault I got PH, or mine. My body has been there throughout my life, without it I wouldn't have my children or grandchildren and I wouldn't have experienced all the wonderful things I have in the past, or will experience in the future. I need to look after it.*
There is no point in doing anything anymore	*I have had that thought for several months now and where has it got me? I know this is because of my depression and if I listen to it, it just fuels it. Besides looking at my activity schedule, I can see that my mood improves in response to activities so there is a point in doing things.*
I can't play with my grandchildren like I used to and so they do not enjoy it as much	*Although I want to play with them like I used to, the fact is I cannot. My grandchildren understand that and they always tell me that they have fun when they come over.*
I get tired more easily and need to take breaks, which makes me not want to do it	*If I slow down and take things at a gentler pace, I can still do the job. It also means that I can enjoy the job more, rather than seeing it as another thing that needs to be done.*
I keep blaming myself for having PH so it must be my fault	*I know it's not my fault for having PH, I don't want it and I am not having these difficulties on purpose.*
People treat me differently since my PH has got worse and I hate the way that feels	*The change I am talking about is people are showing me that they care and are supportive towards me. Although it can feel odd or difficult, they mean well and sometimes I do need support as I have a serious condition.*
	I know my thoughts are not facts and they are being negatively biased by my depression.
	This is a black and white thought – my emotions are not just "happy or sad" and my body is not just "able and unable".
	I am trying to predict the future; I don't know if I will enjoy an activity or not.
	I am over-generalising, just because I cannot do something that I used to do because of my PH, does not mean I can't do anything.

As a result of challenging this thought, Catherine decides to change her thought to:

"It is true that I cannot do some of the things I used to, but that doesn't mean there is no point in doing anything. It is also true that my body is stopping me from doing things, but I can still be happy and achieve success in my life. I need to work with my body and find a balance between what I can do and cannot, being aware that it will change over time".

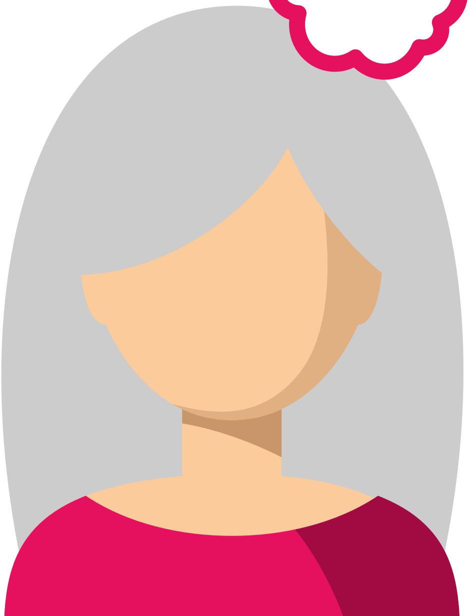

Using the questions on **page 23**, begin to question some of the unhelpful thoughts you identified earlier **(on pages 9 to 21)** by coming up with more helpful thoughts. Make sure to think of responses that you believe in.

It is important to come up with ***realistic*** and more ***compassionate*** alternatives. This means thinking of alternatives that are ***fair*** and ***accurate***, instead of being unfair to yourself or letting the depression bully take over.

Task

2 The two minute rule

As we discussed, rumination, or repetitive negative thinking, is a common symptom of depression. It can also pull people deeper into depression. This exercise is a way of helping you deal with rumination in *two steps*.

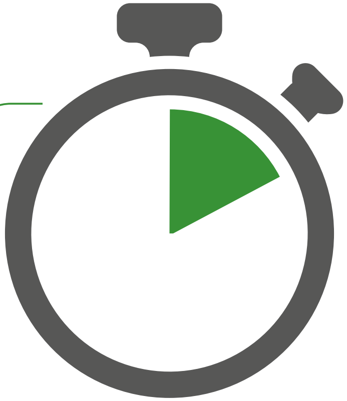

STEP ONE

Timing yourself using a clock, phone or stopwatch, for the next *two minutes* let yourself worry and ruminate without trying to stop yourself.

After two minutes, ask yourself the following three questions:

- ***Have I made any progress towards solving my problem?***
- ***Do I have new insight into understanding my problem?***
- ***Has it made me feel better about my situation?***

If the answer is yes, great!
It sounds as if those two minutes were helpful. However, this is **unlikely** to always be the case as we know rumination can be unhelpful.

If the answer is no...
The chances are that you are ruminating in an **unhelpful** way. Now move onto step two.

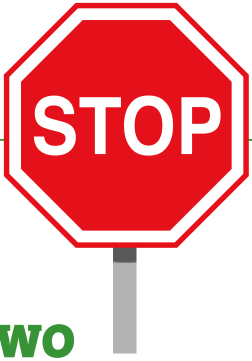

STEP TWO

Use the following strategies to ***interrupt or stop*** your rumination in its tracks.

This might feel difficult at first, but the more time you spend doing it, the better effect it will have at reducing unhelpful thoughts and behaviours and improving your mood.

If you find yourself going around in circles, then try something else on this list.

GO

Gain some distance from the ruminations by saying to yourself, "I am ruminating", "I am doing it again", "My thoughts are not facts", or "I notice that I am having unhelpful thoughts".

GO

Engage in your mindfulness exercises to ground yourself in the here and now (see the activity from booklet one)

GO

Change your behaviour to change your thinking. Look at the lists of activities from booklet two. As you have been experiencing, engaging in activities can help you to focus on something other than your negative thoughts.

3 Problem-solving

Depression can get in the way of us thinking more ***clearly or rationally***, making it difficult for us to problem-solve effectively and challenge negative thoughts. Added to this, people with depression can feel very overwhelmed and are managing different challenges and so need their problem-solving skills.

While you may have been able to problem-solve easier before you experienced symptoms of depression or PH, now it may be harder. There are five steps to follow when problem-solving:

STEP ONE

Write down what the problem is – be as specific as you can.

STEP TWO

Now you see it written down and have gained some distance from it, is it a problem that needs solving or is it a problem that is in your control to solve? You could even try doing something else for a short time before coming back to it. ***If it does need solving, move to step 3.***

STEP THREE

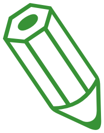

List all of the possible solutions to solving the problem.

Think about what you would have done before experiencing depression, what you hope to do, what someone else would do, or what they would advise. You may even want to get someone else's thoughts on the problem.

STEP FOUR

Chose the best solution to the problem from the list.

STEP FIVE

Now list the steps needed to tackle the problem.

✓

✓

✓

✓

✓

In the next booklet, you will see how Ally uses this to solve his problem of isolating himself from others.

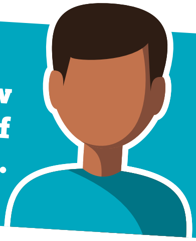

Summary

In this booklet you have explored how changing the way you think can change your mood and how you behave. You have explored some of your unhelpful thinking patterns and started to challenge them using different approaches. With practice, this will become easier, and it will help you break out of your vicious cycles.

As the diagram (from book 1)shows, while Catherine's situation of her grandchildren coming at 10am triggered an unhelpful thought, she has now been able to challenge it and change it to something more positive. This means she has stopped it in its tracks from having a negative impact on her physical sensations, emotions and behaviours.

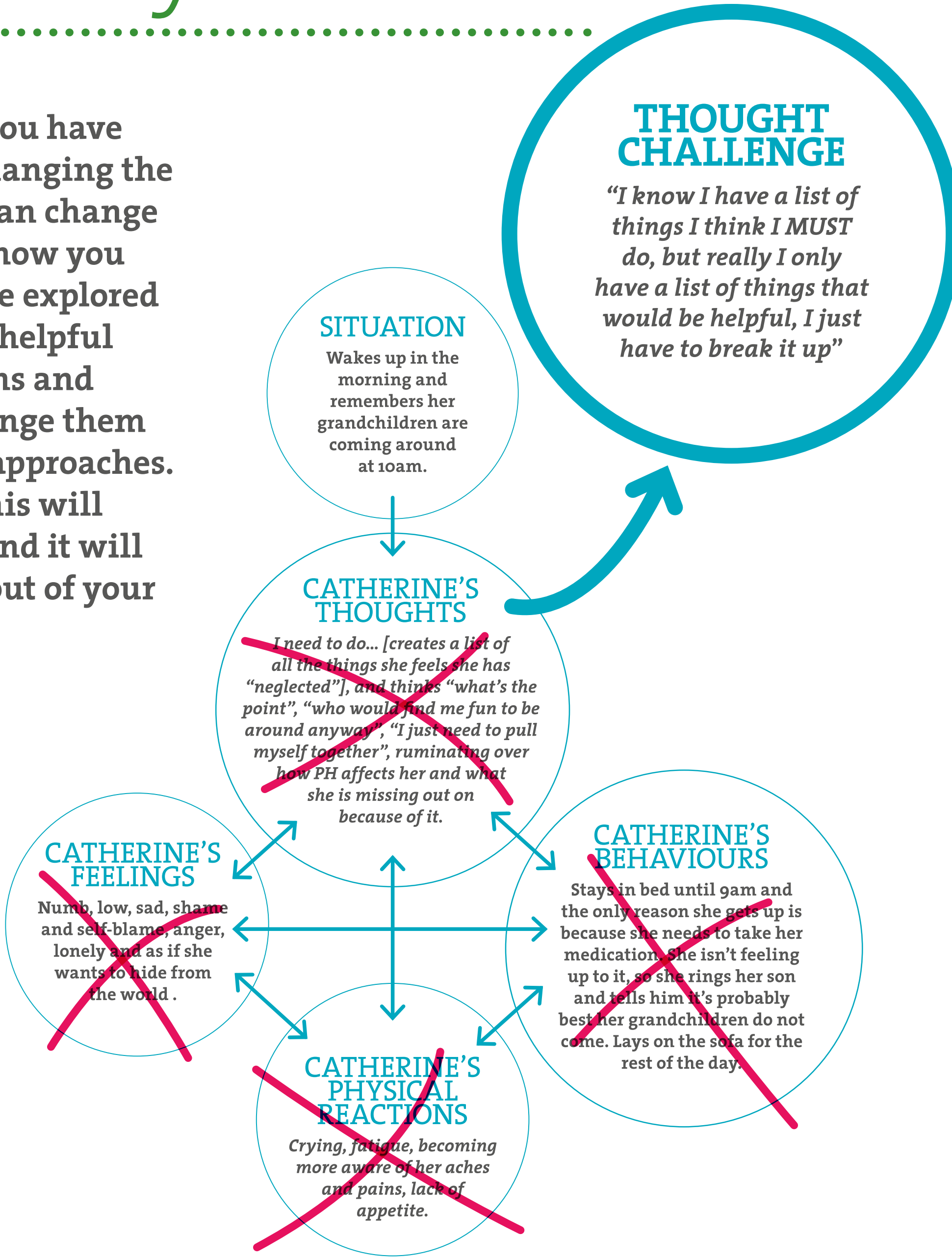

Exercise: Completing a thought diary

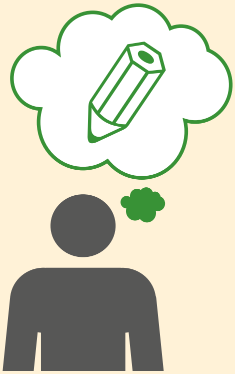

Please use the table (right) to record your thoughts and feelings over the next week. Use the table to also help you to begin challenging your thoughts with more helpful and compassionate thoughts. The more you practice this, the easier it will become.

An example of one of Ally's thoughts has been provided.

**Situation** *Where were you, what was going on?*	**Emotion** *What did you feel?*	**Unhelpful thoughts & thinking patterns** *Be specific*	**Can you use an alternative thought, or more helpful thoughts?**	**Could you use a technique to help manage any discomfort?**	**Did using an alternative thought and/or strategy help with the emotion?**
I was in the pub with my friends, and they were all laughing	I was enjoying myself but then I felt isolated and aware of my body and how I was feeling	"What am I even doing here", "people can see I don't belong here", "I deserve to be miserable at home alone"	My thoughts aren't facts, and besides I can see no one is looking at me. Why should I not be happy, it is just my depression making me think negative things about myself. I am following my schedule rather than my feelings	Thought challenging Mindfulness	Thought challenging did work at first but then I realised it was unhelpful being stuck in my own head. I asked a friend how work was going and I was mindful while listening to him

**Situation** *Where were you, what was going on?*	**Emotion** *What did you feel?*	**Unhelpful thoughts & thinking patterns** *Be specific*	**Can you use an alternative thought, or more helpful thoughts?**	**Could you use a technique to help manage any discomfort?**	**Did using an alternative thought and/ or strategy help with the emotion?**

Task

	Day one
6-7am	P /10 A /10 M /10
7-8am	P /10 A /10 M /10
8-9am	P /10 A /10 M /10
9-10am	P /10 A /10 M /10
10-11am	P /10 A /10 M /10
11-12noon	P /10 A /10 M /10
12noon-1pm	P /10 A /10 M /10
1-2pm	P /10 A /10 M /10
2-3pm	P /10 A /10 M /10
3-4pm	P /10 A /10 M /10
4-5pm	P /10 A /10 M /10
5-6pm	P /10 A /10 M /10
6-7pm	P /10 A /10 M /10
7-8pm	P /10 A /10 M /10
8-9pm	P /10 A /10 M /10
9-10pm	P /10 A /10 M /10
10-11pm	P /10 A /10 M /10
11-12pm	P /10 A /10 M /10

Task	Day two
6-7am	**P** /10 **A** /10 **M** /10
7-8am	**P** /10 **A** /10 **M** /10
8-9am	**P** /10 **A** /10 **M** /10
9-10am	**P** /10 **A** /10 **M** /10
10-11am	**P** /10 **A** /10 **M** /10
11-12noon	**P** /10 **A** /10 **M** /10
12noon-1pm	**P** /10 **A** /10 **M** /10
1-2pm	**P** /10 **A** /10 **M** /10
2-3pm	**P** /10 **A** /10 **M** /10
3-4pm	**P** /10 **A** /10 **M** /10
4-5pm	**P** /10 **A** /10 **M** /10
5-6pm	**P** /10 **A** /10 **M** /10
6-7pm	**P** /10 **A** /10 **M** /10
7-8pm	**P** /10 **A** /10 **M** /10
8-9pm	**P** /10 **A** /10 **M** /10
9-10pm	**P** /10 **A** /10 **M** /10
10-11pm	**P** /10 **A** /10 **M** /10
11-12pm	**P** /10 **A** /10 **M** /10

Task

Day three

Time		
6-7am		P /10 A /10 M /10
7-8am		P /10 A /10 M /10
8-9am		P /10 A /10 M /10
9-10am		P /10 A /10 M /10
10-11am		P /10 A /10 M /10
11-12noon		P /10 A /10 M /10
12noon-1pm		P /10 A /10 M /10
1-2pm		P /10 A /10 M /10
2-3pm		P /10 A /10 M /10
3-4pm		P /10 A /10 M /10
4-5pm		P /10 A /10 M /10
5-6pm		P /10 A /10 M /10
6-7pm		P /10 A /10 M /10
7-8pm		P /10 A /10 M /10
8-9pm		P /10 A /10 M /10
9-10pm		P /10 A /10 M /10
10-11pm		P /10 A /10 M /10
11-12pm		P /10 A /10 M /10

Task	Day four
6-7am	**P** /10 **A** /10 **M** /10
7-8am	**P** /10 **A** /10 **M** /10
8-9am	**P** /10 **A** /10 **M** /10
9-10am	**P** /10 **A** /10 **M** /10
10-11am	**P** /10 **A** /10 **M** /10
11-12noon	**P** /10 **A** /10 **M** /10
12noon-1pm	**P** /10 **A** /10 **M** /10
1-2pm	**P** /10 **A** /10 **M** /10
2-3pm	**P** /10 **A** /10 **M** /10
3-4pm	**P** /10 **A** /10 **M** /10
4-5pm	**P** /10 **A** /10 **M** /10
5-6pm	**P** /10 **A** /10 **M** /10
6-7pm	**P** /10 **A** /10 **M** /10
7-8pm	**P** /10 **A** /10 **M** /10
8-9pm	**P** /10 **A** /10 **M** /10
9-10pm	**P** /10 **A** /10 **M** /10
10-11pm	**P** /10 **A** /10 **M** /10
11-12pm	**P** /10 **A** /10 **M** /10

Task

Day five

6-7am		P /10 A /10 M /10
7-8am		P /10 A /10 M /10
8-9am		P /10 A /10 M /10
9-10am		P /10 A /10 M /10
10-11am		P /10 A /10 M /10
11-12noon		P /10 A /10 M /10
12noon-1pm		P /10 A /10 M /10
1-2pm		P /10 A /10 M /10
2-3pm		P /10 A /10 M /10
3-4pm		P /10 A /10 M /10
4-5pm		P /10 A /10 M /10
5-6pm		P /10 A /10 M /10
6-7pm		P /10 A /10 M /10
7-8pm		P /10 A /10 M /10
8-9pm		P /10 A /10 M /10
9-10pm		P /10 A /10 M /10
10-11pm		P /10 A /10 M /10
11-12pm		P /10 A /10 M /10

Task

Day six

6-7am	P /10 A /10 M /10
7-8am	P /10 A /10 M /10
8-9am	P /10 A /10 M /10
9-10am	P /10 A /10 M /10
10-11am	P /10 A /10 M /10
11-12noon	P /10 A /10 M /10
12noon-1pm	P /10 A /10 M /10
1-2pm	P /10 A /10 M /10
2-3pm	P /10 A /10 M /10
3-4pm	P /10 A /10 M /10
4-5pm	P /10 A /10 M /10
5-6pm	P /10 A /10 M /10
6-7pm	P /10 A /10 M /10
7-8pm	P /10 A /10 M /10
8-9pm	P /10 A /10 M /10
9-10pm	P /10 A /10 M /10
10-11pm	P /10 A /10 M /10
11-12pm	P /10 A /10 M /10

Task

Day seven

6-7am	P /10 A /10 M /10
7-8am	P /10 A /10 M /10
8-9am	P /10 A /10 M /10
9-10am	P /10 A /10 M /10
10-11am	P /10 A /10 M /10
11-12noon	P /10 A /10 M /10
12noon-1pm	P /10 A /10 M /10
1-2pm	P /10 A /10 M /10
2-3pm	P /10 A /10 M /10
3-4pm	P /10 A /10 M /10
4-5pm	P /10 A /10 M /10
5-6pm	P /10 A /10 M /10
6-7pm	P /10 A /10 M /10
7-8pm	P /10 A /10 M /10
8-9pm	P /10 A /10 M /10
9-10pm	P /10 A /10 M /10
10-11pm	P /10 A /10 M /10
11-12pm	P /10 A /10 M /10

Additional resources

If you feel that you need additional information or support, please contact your GP or the PHA UK for advice. We have also included a list of other helpful resources:

OVERCOMING WORRY & ANXIETY
(a self-help programme for people with PH)
www.bit.ly/OvercomingWorryAndAnxiety

NHS SELF-HELP
www.bit.ly/NHSSelfHelp

PHA UK
www.phauk.org

DEPRESSION UK
www.depressionuk.org

SAMARITANS
www.samaritans.org

MIND
www.mind.org.uk

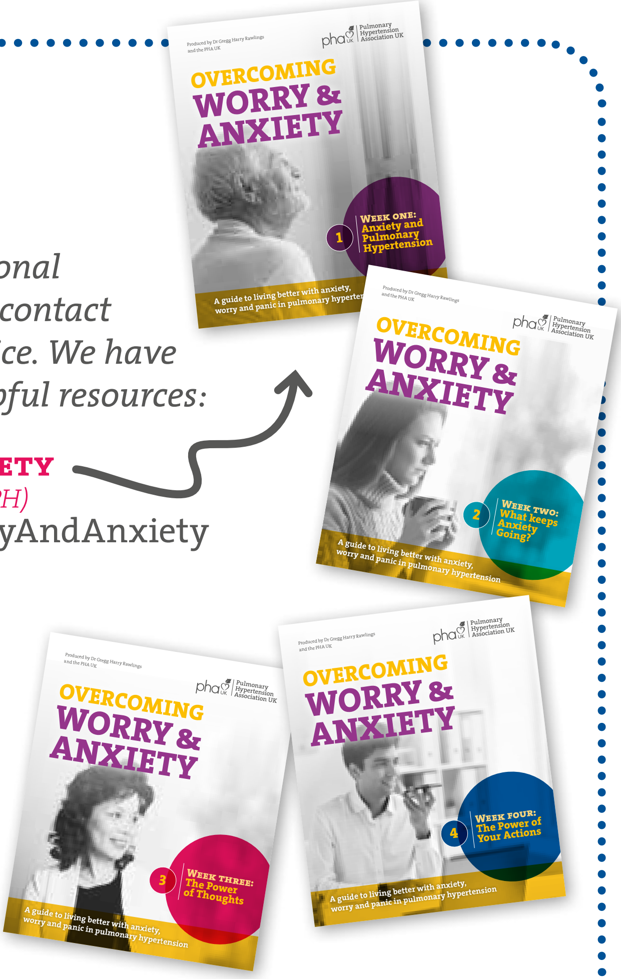

Thoughts of self-harm and suicide can also be common signs of depression. If you start to experience any of these symptoms, please speak to a healthcare professional.

The current study has been designed by researchers working in the UK and it has received ethical approval from an academic institution in the UK (Cardiff University). Participants should be aware and act in accordance with information and governance associated with their country. If you have any questions, please contact your healthcare professional.

For references, see Book 4.

Pulmonary Hypertension Association UK

Nottingham Trent University

Bwrdd Iechyd Prifysgol Caerdydd a'r Fro
Cardiff and Vale University Health Board

No-one ever likes to admit when they are stuck in a bad place, but recognising and acknowledging the problem is the first step to finding a way out.

Dr Helen Knight
GP

To find out more about the PHA UK, visit **www.phauk.org**

Pulmonary Hypertension Association UK

PHA UK Resource Centre, Unit 1, Newton Business Centre, Newton Chambers Road,
Thorncliffe Park, Chapeltown, Sheffield, England S35 2PH

T: 01709 761450 **E:** office@phauk.org @PULHAUK @PHA_UK @PHA ORG UK

Registered Charity No. 1120756. Copyright © 2023 PHA UK. Version 1.2.
