# Supplementary material for: A randomized controlled trial of self‐help cognitive behavioural therapy for depression in adults with pulmonary hypertension
Source: Br J Health Psychol. 2025 Jun 12;30(3):e12800. doi: 10.1111/bjhp.12800 (PMC12159717; doi:10.1111/bjhp.12800)
Supplement: Supplementary file 7 — Data S7. [file BJHP-30-0-s002.pdf]

This series was written by Dr Gregg Rawlings, Abbie Stark, Dr James Gregory, Dr Iain Armstrong and Professor Andrew Thompson

PHA UK

OVERCOMING DEPRESSION AND LOW MOOD

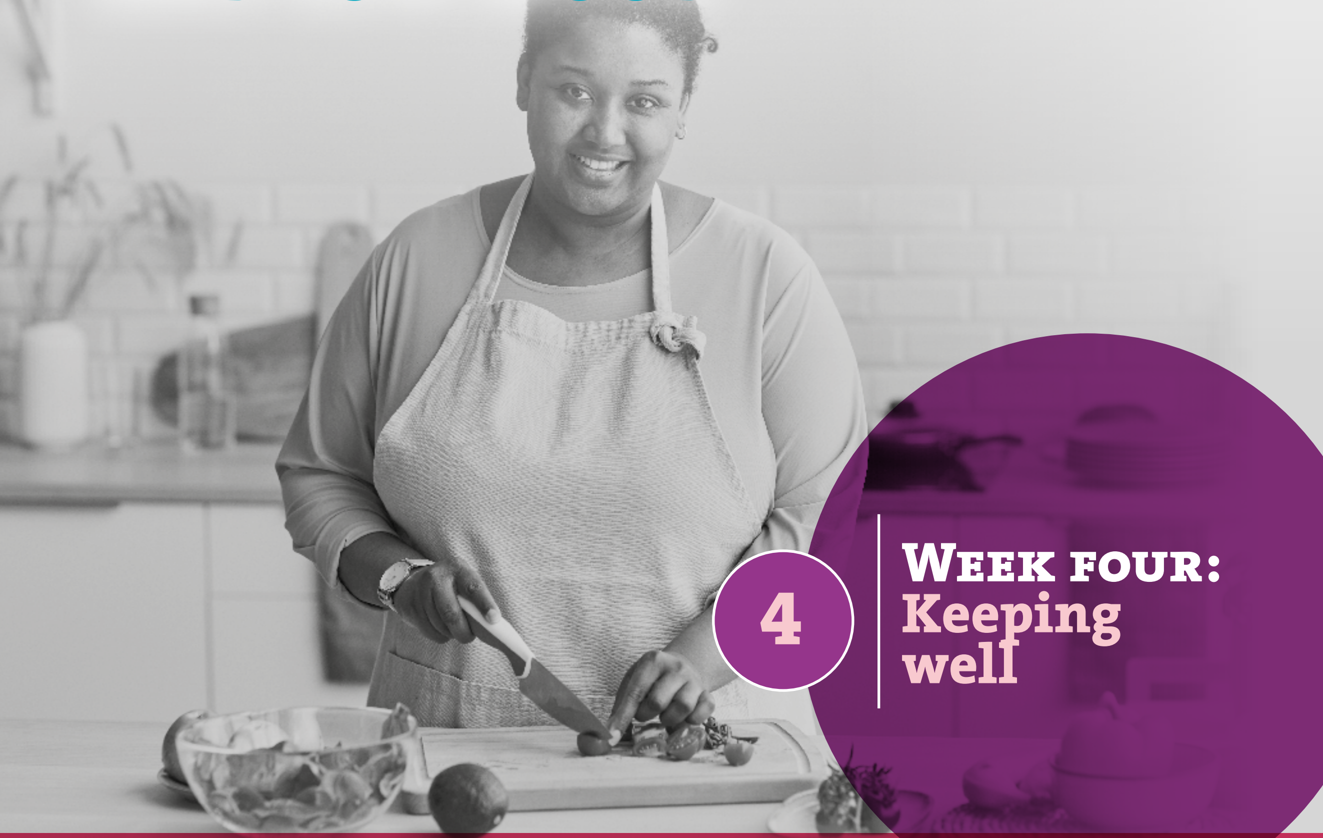

4

Week four: Keeping well

A guide to living better with depression and pulmonary hypertension

PHA UK | Pulmonary Hypertension Association UK

Week three: Keeping well

Overcoming depression and low mood

Here is a summary of what will be covered in this booklet:

- *Strategies for keeping well, such as maintaining a social network, healthy lifestyle and doing meaningful activities*
- *Recognising and managing setbacks*
- *How to continue using the skills in these booklets now and in the future*

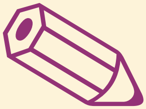

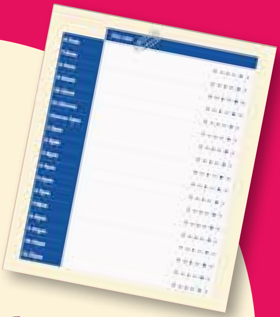

Your week four activity diary starts on page 24, remember to complete it as you work through this booklet.

Keeping **well**

In this final booklet, we will explore six more strategies that will help you to better understand depression and develop coping strategies. This booklet focuses on keeping well; the idea being that you will need to continue using the skills you have learnt beyond week four.

As we discussed in the first week, once people have experienced depression, they are more likely to experience it again in the future. The more strategies you have, the less susceptible you may be to developing depression again. Also, if you do start to experience some of the signs of depression, you are in a great position to know what to do to help yourself.

It may be helpful to think of this as a toolkit.

At the start of these booklets you will have had some helpful and unhelpful ways of coping with depression, but over time as you have engaged with this series, you will have got rid of those unhelpful strategies (or reduced them) and added more helpful ones into your toolkit.

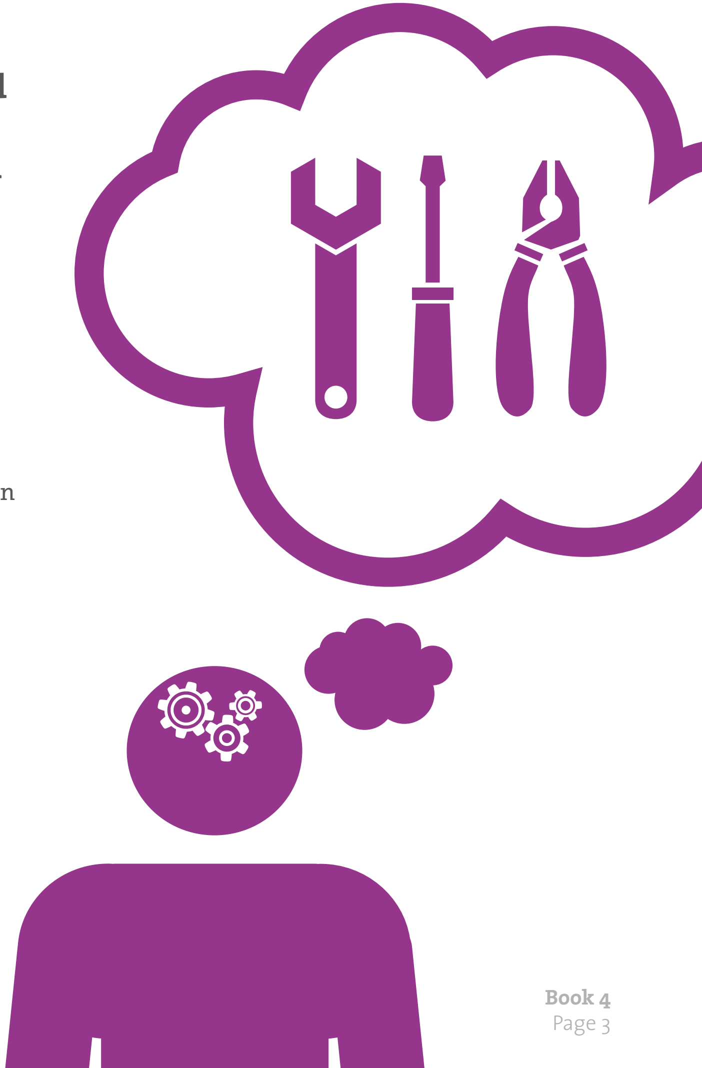

Engaging in ***meaningful activities***

As you have been finding out, engaging in pleasurable and achievable activities can help improve your mood and energy levels, which helps combat your depression. However, it is important to engage in activities that you value in your life, and not just any activity.

The exercise on the right aims to help you recognise what is important to you. What do you want your life to stand for, what do you want to be known for, and what do you want to be guided by? Values are different to goals. Goals can be achieved and ticked off whereas values are something that you strive for.

For example, you cannot '***complete***' being a caring partner, it is something you need to keep working towards. ***There is no correct or wrong value***, and we will all prioritise our values differently. For example, some of us may have prioritised our careers, while others may have focused on another value.

STEP 1

Look at the list of different values and write a few sentences about what it means to you and why it is important.

STEP 2

Then, rate the value in terms of importance to you from 0-10, with 0 being not important at all, and 10 being the most important.

STEP 3

Rate each value in terms of how successfully you have lived this value in the last month from 0-10, with 0 being not successfully at all, and 10 representing you living it extremely successfully.

STEP 4

Look back at your scores, what do you notice? Are you living your life in line with your values? Do you rate a value high in importance but low on success or vice versa? Is there a mismatch between where you put your time and energy and what you actually value in life?

Using the information from this exercise as a compass to guide you, list five activities on page 6 that you plan to do to move closer towards your values. Remember to follow the dos and don'ts from week two and be aware of all or nothing thoughts. Values are not something you either do or don't do.

Exercise:
What's important to you?

Value	*What does it mean to you?*<br>*If it is important to you, why is that?*	*Importance 0-10*	*Success 0-10*
Family			
Parenting			
Social life			
Career			
Education			
Leisure time			
Spirituality			
Local environment			
Health and wellbeing			

Exercise:

5 steps to move closer to your values

Activity three

Activity one

Activity four

Activity two

Activity five

Depression is a fundamental part of many people's lives, yet there appears to be an ongoing negative stigma associated with this condition.

We need to acknowledge and talk about the impact depression has on a person physically, emotionally, psychologically, and spiritually.

Sian Richardson
PHA UK volunteer and former Clinical Nurse Specialist in PH

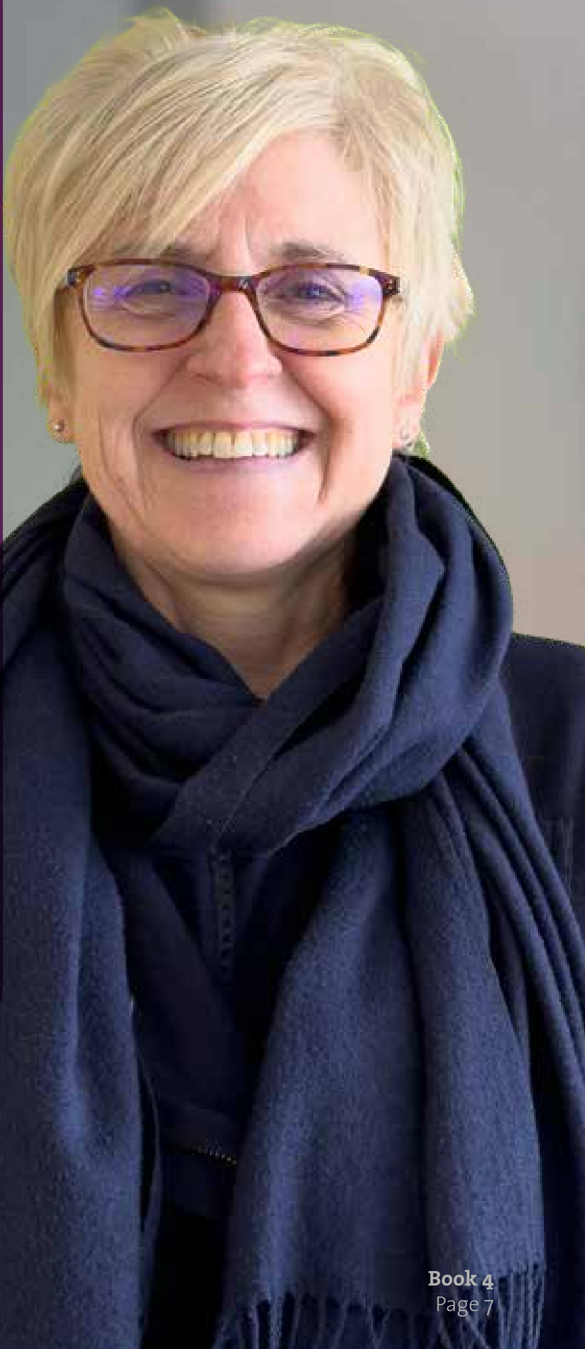

Anxiety
& depression

As we discussed in week one, depression and anxiety are often related. This means, if someone is experiencing symptoms of depression, then there is a good chance they also have anxiety.

And if someone has anxiety, then they may also experience depression. As you have gained a better understanding of your own depression over the last few weeks, you may have also noticed anxiety can be a consequence of your low mood, and it may maintain your depression.

For example, you may have experienced a difficult event causing you anxiety. To manage that anxiety, you avoided situations, people, or your feelings, which can contribute to symptoms of depression. When you do find yourself in certain situations that trigger your anxiety, you may experience self-critical thoughts such as ***"I am useless"***, ***"Everyone is thinking how odd I am"*** and ***"Others are better than me"***. We have seen how such thoughts can feed into our depression.

Anxiety can include symptoms of:

Feeling nervous, anxious or on edge

Not being able to control your worrying

Worrying too much

Having trouble feeling relaxed

Feeling restless

Becoming annoyed easily

Feeling dread, as if something terrible might happen

If you are having any difficulties with your anxiety, there is help available. Please speak to your doctor who should be able to help.

You may also find another series similar to this, but focusing on anxiety in PH, to be helpful. You can access it at ***www.bit.ly/OvercomingWorryAndAnxiety***

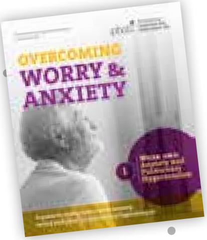

Anger
& depression

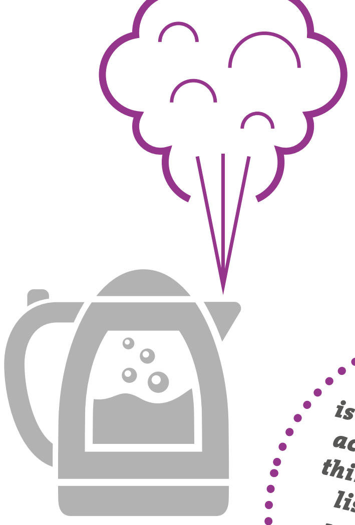

As we explored in the first booklet, anger can be a common sign of depression. For example, being frustrated more often by people, or being quick to anger. People experience anger in different ways, for example:

- Bottling it up, which can help in the short-term but in the long-term can affect their emotional and physical health.
- Turning their anger in on themselves, for example, being angry at themselves and self-bullying.
- Venting it verbally such as shouting and swearing.
- Venting it physically, such as engaging in physical activity often to the point where they push themselves too far and cause injury.

The first step to managing anger is understanding where it comes from.

To do this, we can look at icebergs. The thing about icebergs is that we only see 10% of it, as 90% is underwater. It's the same with anger. People only see the physical signs of anger and not what is underneath the surface. But actually, when we look closer, there are often other emotions causing the anger.

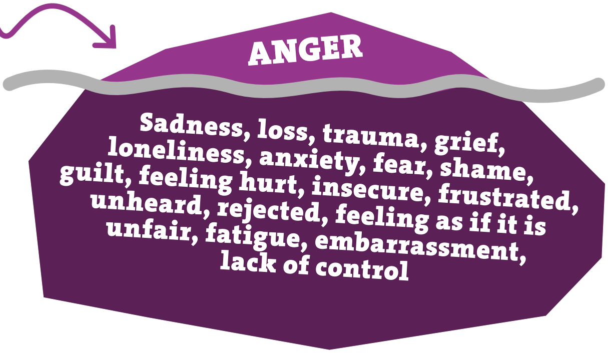

If your anger is high, do some calming activities. This could be things like mindfulness, listening to soothing music, spending time with a pet, and going outside in nature.

Be honest with yourself about how you are feeling and what would help.

When talking to someone about how you feel, be assertive. This means be open, honest and direct.

When you have calmed down, make sure to reflect on the event and what you learnt.

Listen to the other person's point of view.

Be willing to step back from conversations if you are not finding them helpful or your anger is building.

Make sure you stand up for your own rights as well as respecting other people's.

It may even be helpful to share this section on anger with others to help them understand.

Cognitive Behavioural Therapy (CBT) is one of the most effective therapies for depression. From working in the NHS, I have seen first-hand how helpful CBT can be for lots of people and how it can make a big difference to quality of life.

Abbie Stark
Trainee Clinical Psychologist

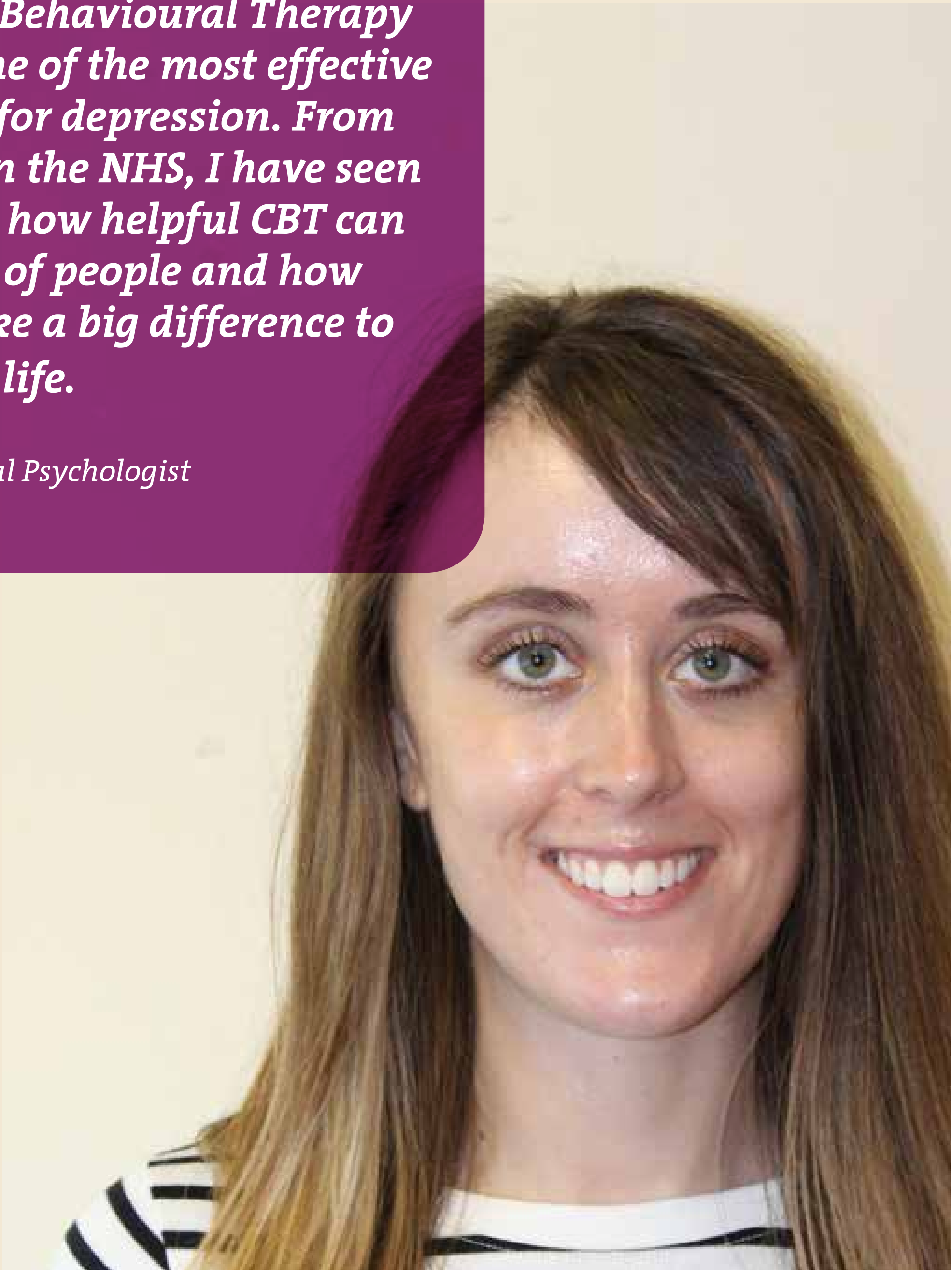

Maintaing a healthy lifestyle

The word 'healthy' is another subjective term, meaning what is healthy for one person, may not be healthy for another – as we are all unique and have our circumstances.

However, there are some things that we know are important factors for maintaining a healthy lifestyle for everyone. You have already learnt about some of them in booklet two, such as the importance of good sleep and keeping mentally and physically active.

Our diet can also be related to our mood. Our body uses what we eat and drink as fuel. Therefore, a well-balanced diet will mean your body can run on better fuel, which will make you mentally and physical stronger.

Here are some suggestions on how to achieve this:

Eat regularly, aiming for three meals a day with only light snacks in-between.

Eating breakfast can increase your mood, making you happier and less irritable.

Limit eating foods that are high in fat or sugar, and those that are highly processed.

Do not 'stress eat' or eat because you are bored – engage in other ways of coping such as exercise, talking to people, hobbies or household tasks.

Looking after your physical appearance and hygiene is also important.

The goal of this is not to meet the expectations of other people or to pretend everything is fine. Quite often when people have depression, they have low motivation to keep up with chores or wash their own clothes or look after their personal hygiene. Remember it is not that they don't want to, or that they are lazy – although they might be saying this to themselves. It can be helpful to make a list of essential activities for you to do, even if you are feeling really low. Even if it takes a lot longer for you to do, or you need to rest afterwards, make sure you tick off your list.

For example:

- ✓ ***Brush my teeth***
- ✓ ***Wash my face and certain parts of my body***
- ✓ ***Comb my hair***
- ✓ ***Make my bed***
- ✓ ***Open a window***
- ✓ ***Clean the dishes***

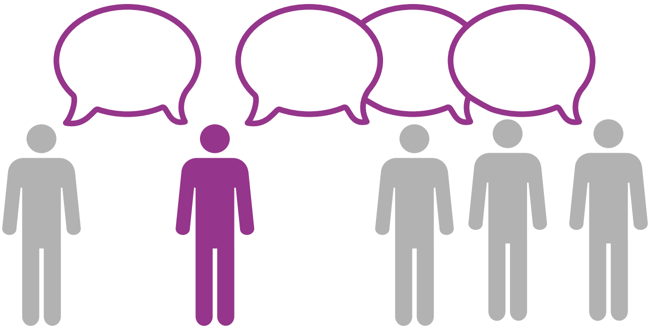

Maintaining your social network

As we explored in booklet one, feeling connected to other people is important. When people are depressed, it is common that they isolate themselves from others.

This can be for different reasons. For example, they feel ***vulnerable*** or ***embarrassed*** and want to protect themselves. Social situations can feel ***exhausting*** or ***too much***, or they may not see the point because of negative thoughts such as ***"Who would want to be friends with me"?***

However, it is important to keep up your social networks as they can be a positive resource in helping you to recover from depression and preventing your depression becoming worse. Also, when you are feeling better, it may be more difficult to develop relationships with others, resulting in you feeling lonely and isolated – which may then increase your risk of experiencing depression again.

We know feeling isolated is related to depression and anxiety in people with PH.

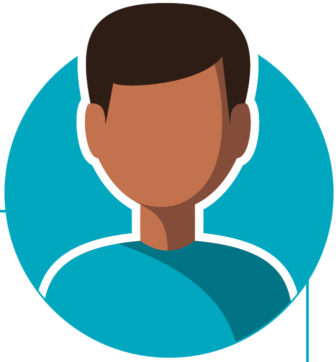

For example, Ally has noticed that his feelings about social interactions are ***not 'all or nothing'***, for example, ***"I am either sociable or not sociable"*** and instead they are on a ***continuum*** with grey areas. He gained this knowledge from booklet three on negative thinking patterns. He also realised while at first, he might not feel up to social interactions, after speaking to someone his mood can lift, even if it was a little. He plotted the different types of interactions on a line and used it to guide when he is feeling low and motivated.

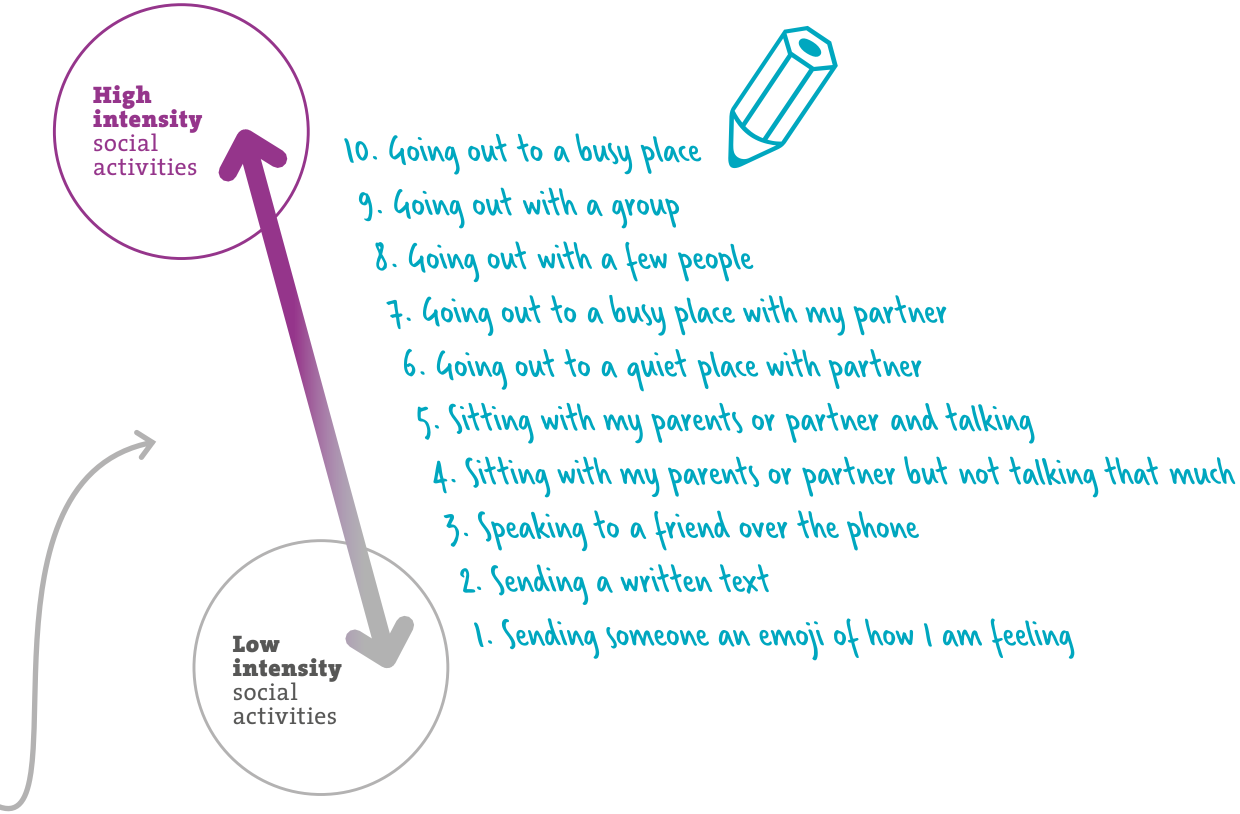

Sometimes he felt able to do more intense social activities (for Ally, this was items 7-10), but even when he was feeling unmotivated, he would make sure to do activities 1-3 with his partner each day.

Over time, he began to feel close with his partner again as they both felt more able to open up and be honest with each other. They realised they were both using strategies to cope that helped in the short-term, but not in the long-term. His relationships with his friends also improved because the longer he spent with them, the more he felt they understood each other.

There can be some common challenges people with PH experience due to the condition. People with PH may be asked questions about their condition, how it impacts their life, why they need to take medication, or why they cannot do something. This may be caused by the lack of knowledge and awareness of PH by others. This can leave people with PH feeling embarrassed, angry, and anxious as they may not know what to say.

Ally noticed this and so decided to use his problem-solving skills to think of a solution. He created a few responses which he practiced in the mirror alone. This ranged from a brief response such as, ***"I have a condition that affects my breathing"*** or ***"I have a condition that affects my breathing and the medication helps me"***, as well as strategies to help move the conversation on quickly , such as ***"anyway, how are you, I hear you have been away on holiday?"***

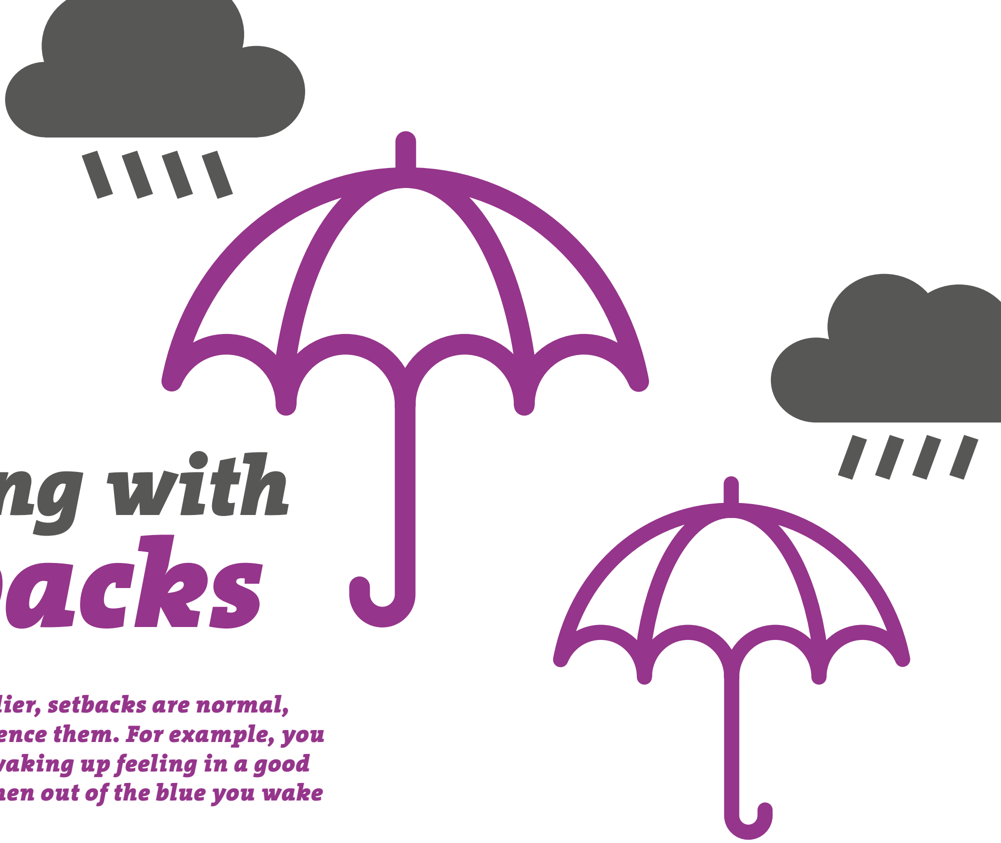

Dealing with **setbacks**

As we discussed earlier, setbacks are normal, and you will experience them. For example, you may go a few days waking up feeling in a good or 'OK' mood, and then out of the blue you wake up feeling low.

We also know that PH can be very unpredictable and so some of your symptoms may flare up, affecting what you had planned.

In this case it is helpful to make a plan.

Here is Catherine's setback plan for when she has a 'bad' day:

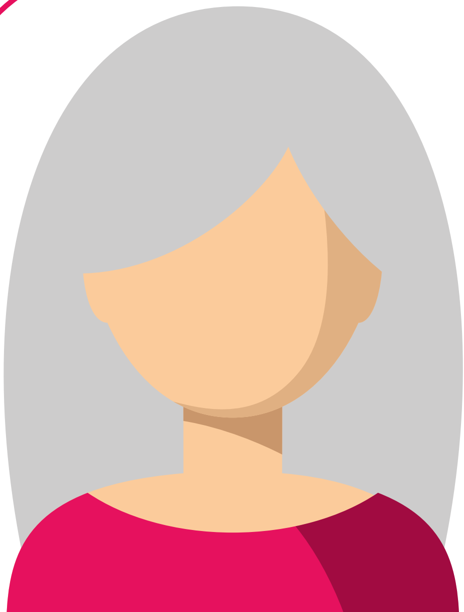

Situation	What I may want to do	What I should do	What I need from others
Waking up in the morning and feeling unmotivated	Stay in bed	Follow my schedule and get up. If I need to rest due to my PH, I can do it downstairs as I know my bedroom is for sleeping	Maybe a text from a friend cheering me on
Going for a walk and needing to rest	Say 'forget it, I want to go home'	Sit and be mindful while I rest, take in my surroundings being in the here and now	Sit with me and be patient
Talking about how my PH symptoms have got worse or talking about what I am not able to do that is important to me	Avoid the conversation and pretend it is not happening	Talking about how I am feeling, recognising that I am upset and rather than self-bullying, be kind to myself	Recognise it is hard but must be spoken about in a compassionate way
Someone making a joke about me being unfit because I am out of breath	Reply in an angry tone of voice or go home and avoid situations where it might happen again	Ignore the person but realise that I am upset and should do something that is self-care, or explain to them that I have a medical condition that affects my breathing	People to be understanding and think before they speak. I would also like to share how I am feeling with a friend

Over the page, identify your own situations and develop your own plans

Exercise:

setback plan

Can you identify any situations that may feed your depression? And what is your plan to help manage it?

Situation	What I may want to do	What I should do	What I need from others

Final **thoughts**

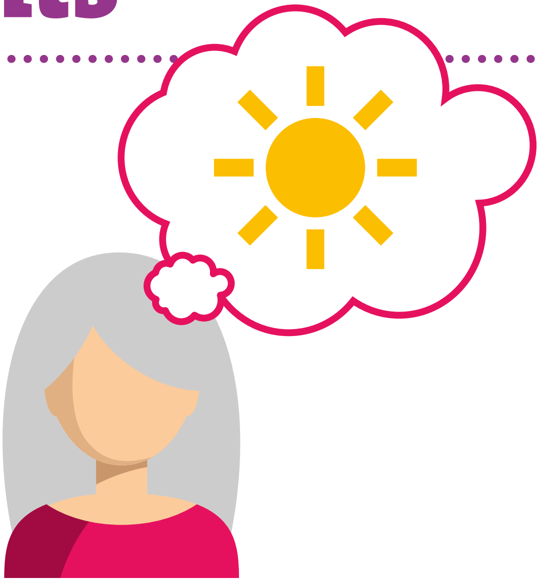

Depression is the most common mental health difficulty, with many people with PH experiencing it. It is important to remember that you are not alone. Many of the signs of depression are normal reactions to difficult and upsetting experiences. However, when they become too intense, go on for too long or stop you from living your life, this is when it becomes unhelpful and is not a normal response.

The knowledge and skills you have been practicing over the past four weeks has been shown to help people with depression.

However, in order for them to be useful, ***you need to keep practicing them beyond week four.*** As we discussed in booklet one, mental health is like physical health – as soon as you stop doing helpful activities, your health may begin to suffer. It's therefore important to find things that work for yourself.

When you notice your mood might be slipping into depression, try to act towards yourself in the way that you would towards someone else that you care for. Recognise it might be part of the PH journey and allow yourself to self-care and use some of the techniques in our guides to support yourself.

Look at Catherine's diagram, on the next page, showing her original difficulty that you read about in the first week. As you can see, she now has a range of different skills to cope better with her depression.

You may wish to revisit your own diagram from week one and think about the strategies you have learned for coping better.

✓ *Changing thoughts and behaviour to change emotions*

✓ *Understanding her emotions*

✓ *Talking to others about her emotions*

SITUATION

Wakes up in the morning and remembers her grandchildren are coming around at 10am.

CATHERINE'S THOUGHTS

I need to do... [creates a list of all the things she feels she has "neglected"], and thinks "what's the point", "who would find me fun to be around anyway", "I just need to pull myself together", ruminating over how PH affects her and what she is missing out on because of it.

CATHERINE'S FEELINGS

Numb, low, sad, shame and self-blame, anger, lonely and as if she wants to hide from the world .

CATHERINE'S BEHAVIOURS

Stays in bed until 9am and the only reason she gets up is because she needs to take her medication. She isn't feeling up to it, so she rings her son and tells him it's probably best her grandchildren do not come. Lays on the sofa for the rest of the day.

CATHERINE'S PHYSICAL REACTIONS

Crying, fatigue, becoming more aware of her aches and pains, lack of appetite.

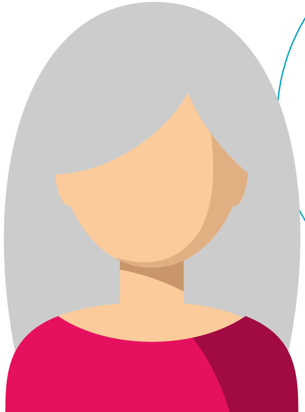

✓ Thought-challenging methods

✓ 2-minute rumination rule

✓ Problem-solving

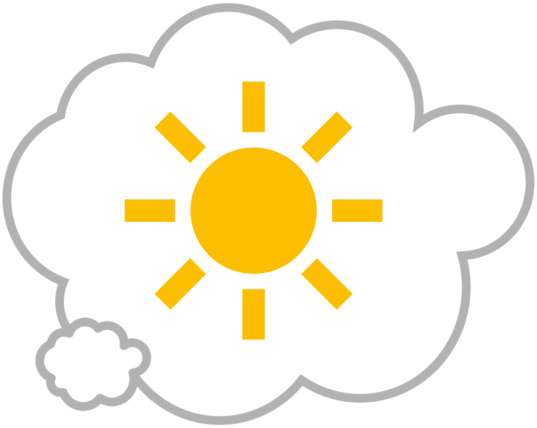

✓ Activity scheduling

✓ Engaging in meaningful activities

✓ Keeping mentally and physically active

✓ Maintaining a healthy lifestyle

✓ Maintaining social network

✓ Mindfulness

✓ Understanding what her symptoms are

Exercise:

A letter to yourself

An important part of ending Cognitive Behavioural Therapy (CBT) is reflecting on what you have learnt and discussing your hopes and fears for the future. One way of doing this is in a letter.

Often therapists write to their clients summarising their work together. The letter can also be a reminder for the person after therapy when they are finding events difficult and need to be reminded of certain skills, or just to hear words of encouragement.

For the activity this week, I want you to spend at least 20 minutes writing a letter to the version of you before you started this series on depression and PH.

You may want to write about how depression was affecting your life and how it interacted with PH. You could write about the knowledge and skills you have learnt since then, what has been helpful, and what things you know now that are not helpful. You may want to write about your hopes for the future, understanding that you may experience difficult times and how you could cope in a helpful way.

If you prefer, you could write your letter on additional paper or type it on your computer. You could get creative and write the letter to you before you started to read this series on depression, or to another person with PH who is experiencing symptoms of depression.

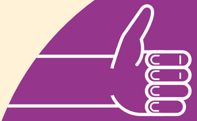

Remember to be aware of negative thinking such as self-criticism. Be compassionate and kind in your letter, and perhaps offer words of wisdom and hope.

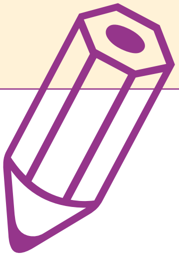

Dear me,

If you feel able, please send your letter with your unique ID number to confidential@phauk.org where it will be read by myself, Abbie Stark and a member of PHA UK. We want you to know that you are being heard and listened to.

You will also be asked if you are happy for your letter to be used for research as it will help us to better understand how depression effects people with PH. If we publish this research, we will anonymise your writing, which means people will not know the letter is yours.

Dr Gregg H. Rawlings
Clinical Psychologist
Nottingham Trent University

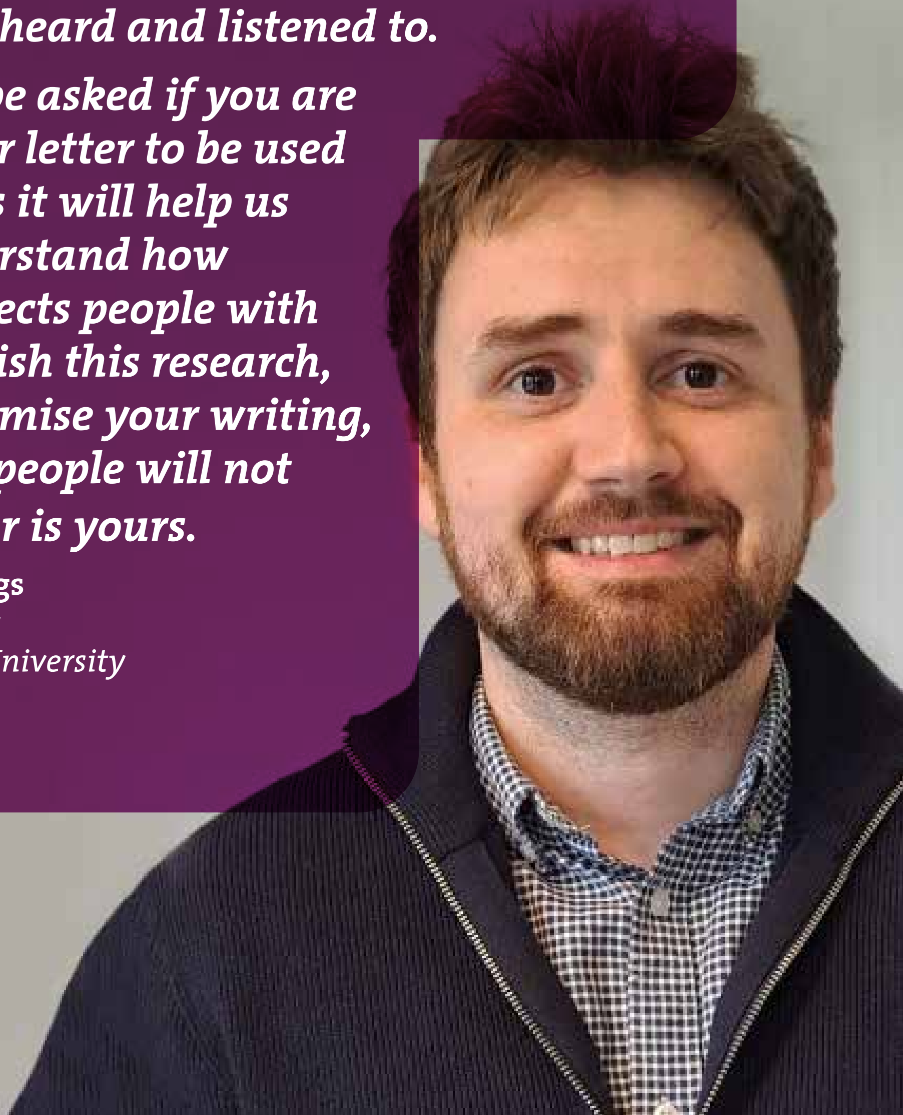

Summary

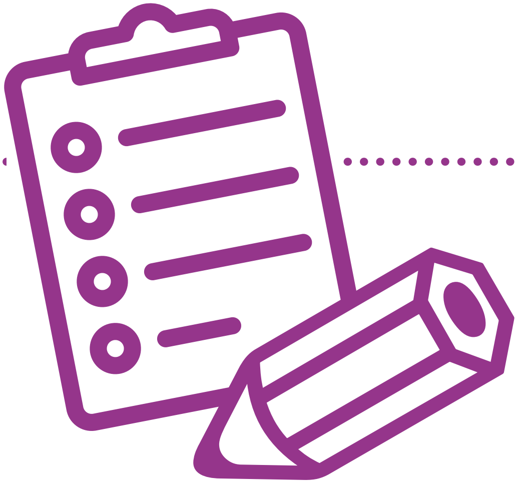

Firstly, we want to thank you for taking the brave steps towards learning to manage your depression.

Depression by its very nature can stop people from reaching out for help, keeping them stuck in a loop of low mood, negative thoughts and unhelpful behaviours. We also want to thank you for how hard you worked throughout the series.

It is important to remember that you did not ask for, or deserve, depression or PH.

We know people with PH are more likely to experience challenges in their life than people without a long-term health condition. We hope you have learnt a range of techniques, including the importance of being kind and compassionate to yourself.

You will be contacted soon via email to complete a series of questionnaires. You will be contacted again one month later to complete the questionnaires again. Following this, you will be sent a final questionnaire to provide your feedback about taking part in this study and the self-help materials.

We want to thank you for how hard you have worked throughout these booklets, and we hope you have found them helpful. We wish you all the best as you continue your journey on managing depression.

Task

	Day one
**6-7am**	**P** /10 **A** /10 **M** /10
**7-8am**	**P** /10 **A** /10 **M** /10
**8-9am**	**P** /10 **A** /10 **M** /10
**9-10am**	**P** /10 **A** /10 **M** /10
**10-11am**	**P** /10 **A** /10 **M** /10
**11-12noon**	**P** /10 **A** /10 **M** /10
**12noon-1pm**	**P** /10 **A** /10 **M** /10
**1-2pm**	**P** /10 **A** /10 **M** /10
**2-3pm**	**P** /10 **A** /10 **M** /10
**3-4pm**	**P** /10 **A** /10 **M** /10
**4-5pm**	**P** /10 **A** /10 **M** /10
**5-6pm**	**P** /10 **A** /10 **M** /10
**6-7pm**	**P** /10 **A** /10 **M** /10
**7-8pm**	**P** /10 **A** /10 **M** /10
**8-9pm**	**P** /10 **A** /10 **M** /10
**9-10pm**	**P** /10 **A** /10 **M** /10
**10-11pm**	**P** /10 **A** /10 **M** /10
**11-12pm**	**P** /10 **A** /10 **M** /10

Task	Day two
6-7am	P /10 A /10 M /10
7-8am	P /10 A /10 M /10
8-9am	P /10 A /10 M /10
9-10am	P /10 A /10 M /10
10-11am	P /10 A /10 M /10
11-12noon	P /10 A /10 M /10
12noon-1pm	P /10 A /10 M /10
1-2pm	P /10 A /10 M /10
2-3pm	P /10 A /10 M /10
3-4pm	P /10 A /10 M /10
4-5pm	P /10 A /10 M /10
5-6pm	P /10 A /10 M /10
6-7pm	P /10 A /10 M /10
7-8pm	P /10 A /10 M /10
8-9pm	P /10 A /10 M /10
9-10pm	P /10 A /10 M /10
10-11pm	P /10 A /10 M /10
11-12pm	P /10 A /10 M /10

Task

Day three

6-7am	P /10 A /10 M /10
7-8am	P /10 A /10 M /10
8-9am	P /10 A /10 M /10
9-10am	P /10 A /10 M /10
10-11am	P /10 A /10 M /10
11-12noon	P /10 A /10 M /10
12noon-1pm	P /10 A /10 M /10
1-2pm	P /10 A /10 M /10
2-3pm	P /10 A /10 M /10
3-4pm	P /10 A /10 M /10
4-5pm	P /10 A /10 M /10
5-6pm	P /10 A /10 M /10
6-7pm	P /10 A /10 M /10
7-8pm	P /10 A /10 M /10
8-9pm	P /10 A /10 M /10
9-10pm	P /10 A /10 M /10
10-11pm	P /10 A /10 M /10
11-12pm	P /10 A /10 M /10

Task

Day four

6-7am		**P** /10 **A** /10 **M** /10
7-8am		**P** /10 **A** /10 **M** /10
8-9am		**P** /10 **A** /10 **M** /10
9-10am		**P** /10 **A** /10 **M** /10
10-11am		**P** /10 **A** /10 **M** /10
11-12noon		**P** /10 **A** /10 **M** /10
12noon-1pm		**P** /10 **A** /10 **M** /10
1-2pm		**P** /10 **A** /10 **M** /10
2-3pm		**P** /10 **A** /10 **M** /10
3-4pm		**P** /10 **A** /10 **M** /10
4-5pm		**P** /10 **A** /10 **M** /10
5-6pm		**P** /10 **A** /10 **M** /10
6-7pm		**P** /10 **A** /10 **M** /10
7-8pm		**P** /10 **A** /10 **M** /10
8-9pm		**P** /10 **A** /10 **M** /10
9-10pm		**P** /10 **A** /10 **M** /10
10-11pm		**P** /10 **A** /10 **M** /10
11-12pm		**P** /10 **A** /10 **M** /10

Task

Time	Day five
6-7am	P /10 A /10 M /10
7-8am	P /10 A /10 M /10
8-9am	P /10 A /10 M /10
9-10am	P /10 A /10 M /10
10-11am	P /10 A /10 M /10
11-12noon	P /10 A /10 M /10
12noon-1pm	P /10 A /10 M /10
1-2pm	P /10 A /10 M /10
2-3pm	P /10 A /10 M /10
3-4pm	P /10 A /10 M /10
4-5pm	P /10 A /10 M /10
5-6pm	P /10 A /10 M /10
6-7pm	P /10 A /10 M /10
7-8pm	P /10 A /10 M /10
8-9pm	P /10 A /10 M /10
9-10pm	P /10 A /10 M /10
10-11pm	P /10 A /10 M /10
11-12pm	P /10 A /10 M /10

Task	Day six
6-7am	**P** /10 **A** /10 **M** /10
7-8am	**P** /10 **A** /10 **M** /10
8-9am	**P** /10 **A** /10 **M** /10
9-10am	**P** /10 **A** /10 **M** /10
10-11am	**P** /10 **A** /10 **M** /10
11-12noon	**P** /10 **A** /10 **M** /10
12noon-1pm	**P** /10 **A** /10 **M** /10
1-2pm	**P** /10 **A** /10 **M** /10
2-3pm	**P** /10 **A** /10 **M** /10
3-4pm	**P** /10 **A** /10 **M** /10
4-5pm	**P** /10 **A** /10 **M** /10
5-6pm	**P** /10 **A** /10 **M** /10
6-7pm	**P** /10 **A** /10 **M** /10
7-8pm	**P** /10 **A** /10 **M** /10
8-9pm	**P** /10 **A** /10 **M** /10
9-10pm	**P** /10 **A** /10 **M** /10
10-11pm	**P** /10 **A** /10 **M** /10
11-12pm	**P** /10 **A** /10 **M** /10

Task	Day seven
6-7am	**P** /10 **A** /10 **M** /10
7-8am	**P** /10 **A** /10 **M** /10
8-9am	**P** /10 **A** /10 **M** /10
9-10am	**P** /10 **A** /10 **M** /10
10-11am	**P** /10 **A** /10 **M** /10
11-12noon	**P** /10 **A** /10 **M** /10
12noon-1pm	**P** /10 **A** /10 **M** /10
1-2pm	**P** /10 **A** /10 **M** /10
2-3pm	**P** /10 **A** /10 **M** /10
3-4pm	**P** /10 **A** /10 **M** /10
4-5pm	**P** /10 **A** /10 **M** /10
5-6pm	**P** /10 **A** /10 **M** /10
6-7pm	**P** /10 **A** /10 **M** /10
7-8pm	**P** /10 **A** /10 **M** /10
8-9pm	**P** /10 **A** /10 **M** /10
9-10pm	**P** /10 **A** /10 **M** /10
10-11pm	**P** /10 **A** /10 **M** /10
11-12pm	**P** /10 **A** /10 **M** /10

Additional resources

If you feel that you need additional information or support, please contact your GP or the PHA UK for advice. We have also included a list of other helpful resources:

OVERCOMING WORRY & ANXIETY
(a self-help programme for people with PH)
www.bit.ly/OvercomingWorryAndAnxiety

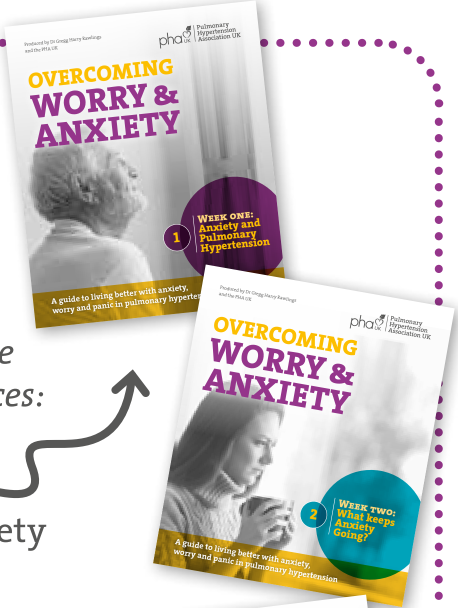

NHS SELF-HELP
www.bit.ly/NHSSelfHelp

PHA UK
www.phauk.org

DEPRESSION UK
www.depressionuk.org

SAMARITANS
www.samaritans.org

MIND
www.mind.org.uk

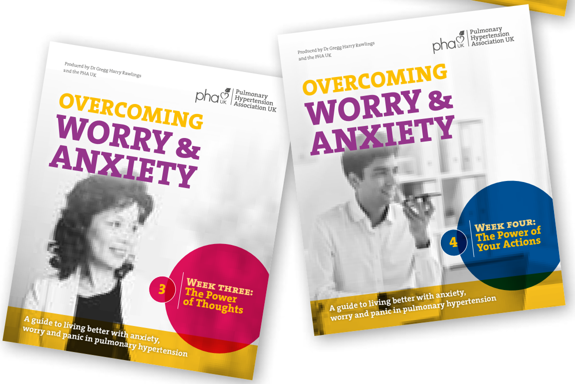

!

Thoughts of self-harm and suicide can also be common signs of depression. If you start to experience any of these symptoms, please speak to a healthcare professional.

The current study has been designed by researchers working in the UK and it has received ethical approval from an academic institution in the UK (Cardiff University). Participants should be aware and act in accordance with information and governance associated with their country. If you have any questions, please contact your healthcare professional.

References

The contents of this booklet have been adapted to make the information specific to pulmonary hypertension for the purpose of this series on anxiety from the following sources:

Anonymous. (date unknown).
Pacing for pain and fatigue [leaflet].
Psychology Tools Limited. Retrieved from: https://www.psychologytools.com/resources/pacing-for-pain-and-fatigue

Centre for Clinical Interventions (2019).
Depression.
Retrieved from: https://www.cci.health.wa.gov.au/Resources/Looking-After-Yourself/Depression

Rawlings, G.H, Thompson, A.R., Armstrong, I., & Beail, N.(2020).
Overcoming worry and anxiety: A guide to living better with anxiety, worry and pulmonary hypertension

PHA UK Pulmonary Hypertension Association UK

NTU Nottingham Trent University

GIG CYMRU NHS WALES | Bwrdd Iechyd Prifysgol Caerdydd a'r Fro | Cardiff and Vale University Health Board

To find out more about the PHA UK, visit **www.phauk.org**

Pulmonary Hypertension Association UK

PHA UK Resource Centre, Unit 1, Newton Business Centre, Newton Chambers Road, Thorncliffe Park, Chapeltown, Sheffield, England S35 2PH

T: 01709 761450 **E:** office@phauk.org @PULHAUK @PHA_UK @PHA ORG UK

Registered Charity No. 1120756. Copyright © 2023 PHA UK. Version 1.2.
